# Ligand-dependent stereoselective Suzuki–Miyaura cross-coupling reactions of β-enamido triflates

**DOI:** 10.3762/bjoc.17.179

**Published:** 2021-10-29

**Authors:** Tomáš Chvojka, Athanasios Markos, Svatava Voltrová, Radek Pohl, Petr Beier

**Affiliations:** 1 Institute of Organic Chemistry and Biochemistry of the Czech Academy of Sciences, Flemingovo nám. 2, 166 10 Prague, Czech Republic; 2 Department of Organic Chemistry, Faculty of Science, Charles University, Hlavova 2030/8, CZ-128 43 Prague 2, Czech Republic

**Keywords:** enamides, isomerization, Suzuki–Miyaura coupling, vinyl triflates

## Abstract

The stereoselective Suzuki–Miyaura cross-coupling of (*Z*)-β-enamido triflates is demonstrated. Depending on the nature of the ligand in the palladium catalyst, either retention or inversion of the configuration during the synthesis of β,β-diaryl-substituted enamides is observed. Thus, the method provides synthetic access to both isomers of the target enamides from (*Z*)-β-enamido triflates.

## Introduction

Enamides are substrates of high value in organic synthesis [1–2]. Their multifacial reactivity has been explored in asymmetric alkylations [3], hydroalkynylations [4], trifluoromethylcyanations [5], heterocycle synthesis [6], asymmetric acylations [7], hydroborations [8], hydrogenations [9], etc. They are also important pharmacophores, which display a range of cytotoxic, antifungal, or antibiotic properties [10–12]. Modern stereoselective syntheses leading to highly substituted enamides include cross-coupling of vinyl (pseudo)halides or organoboron compounds [13], hydroamidation of alkynes [14–16], ynamide functionalization [17–19], or isomerization of *N*-allyl amides [20], but still possess drawbacks, especially for stereoselective synthesis of tri- and tetrasubstituted enamides.

**Scheme 1:**
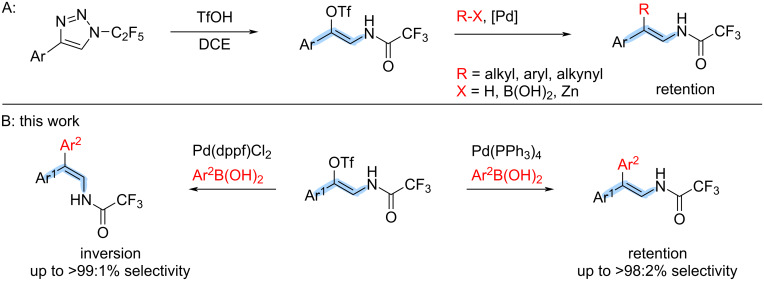
A: Synthesis of (*Z*)-β-enamido triflates and subsequent stereoselective cross-coupling reactions. B: Ligand-controlled stereoselective synthesis of β,β-diaryl-substituted enamides.

Recently, we have reported a triflic acid-mediated reaction of *N*-fluoroalkyl-1,2,3-triazoles leading to (*Z*)-β-enamido triflates [21] and Lewis acid-mediated reaction to (*Z*)-β-enamido fluorides [22] and halovinyl imidoyl halides [23]. In addition, Li and co-workers extended the scope of accessible (*Z*)-β-enamido triflates by denitrogenative reaction of *N*1-*H*-1,2,3-triazoles in the presence of acyl halides and sodium triflate [24]. These enamido triflates and halides were found to undergo cross-coupling reactions with retention of configuration on the double bond and served as valuable starting materials for the synthesis of functionalized β,β-disubstituted enamides (Scheme 1A) [21,23].

In the last decade, only a few reports describing isomerization of the double bond of vinyl (pseudo)halides during the Suzuki coupling have been published [25–29]. Typically, inversion of configuration occurs on substrates containing a double bond in conjugation with an electron-withdrawing group, such as the carbonyl group in enones [27,30]. We hypothesized that (*Z*)-β-enamido triflates could, during the Suzuki cross-coupling, undergo isomerization of the double bond, similarly to enones, and thus serve as starting materials to either (*E*) or (*Z*)-isomers of enamides depending on the conditions used. Here we present a study of the effect of ligand on the stereoselective outcome of the Suzuki cross-coupling reaction of various (*Z*)-β-enamido triflates (Scheme 1B).

## Results and Discussion

We initiated our study by examining the Suzuki cross-coupling of vinyl triflate **1a** and 3-nitrophenylboronic acid. First, the influence of the catalyst on the stereoselective outcome of the reaction was studied (Table 1). The use of Pd(PPh_3_)_4_ resulted in the formation of isomeric products **2aa** and **3aa** in a 93:7 ratio (Table 1, entry 1). Other catalysts led to significant loss of stereoselectivity on the double bond (Table 1, entries 2–9). When Pd(dppf)Cl_2_ was employed, isomeric product **3aa** was preferentially formed (**2aa**/**3aa**, 29:71, Table 1, entry 6). Although other catalysts, such as Pd(acac)_2_, Pd(dba)_2_, and Pd(OAc)_2_(PPh_3_)_2_ showed good selectivity to inversion product **3aa**, the products were formed in low yields (Table 1, entries 7–9). Further screening of the solvent and the base led to the identification of Pd(PPh_3_)_4_ (10 mol %), arylboronic acid (1 equiv), K_3_PO_4_ (2 equiv) in THF/H_2_O 1:1 as ideal conditions providing enamides **2aa** and **3aa** in a 93:7 ratio in 90% ^19^F NMR yield (Table S1 in Supporting Information File 1).

**Table 1:** The effect of different catalysts on product yields and ratios.

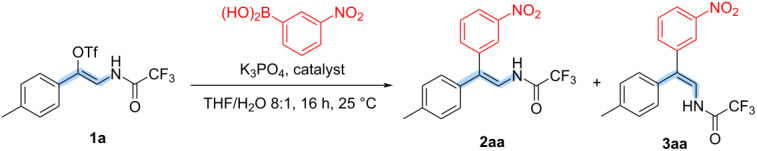			

Entry	Catalyst (10 mol %)	Yield (%)^a^	**2aa/3aa**

**1**	**Pd(PPh** **_3_** **)** **_4_**	**70**	**93:7**
2	PdCl_2_(PPh_3_)_2_	80	81:19
3	2-(2'-di-*tert*-butylphosphine)biphenylpalladium(II) acetate	42	83:17
4	Pd(*t*-Bu_3_P)_2_	46	48:52
5	Pd(dtbpf)Cl_2_	66	33:67
**6**	**Pd(dppf)Cl** **_2_**	**70**	**29:71**
7	Pd(acac)_2_	34	24:76
8	Pd(dba)_2_	23	22:78
9	Pd(OAc)_2_(PPh_3_)_2_	11	18:82

^a^Combined ^19^F NMR yield of **2aa** and **3aa** using PhCF_3_ as an internal standard.

**Scheme 2:**
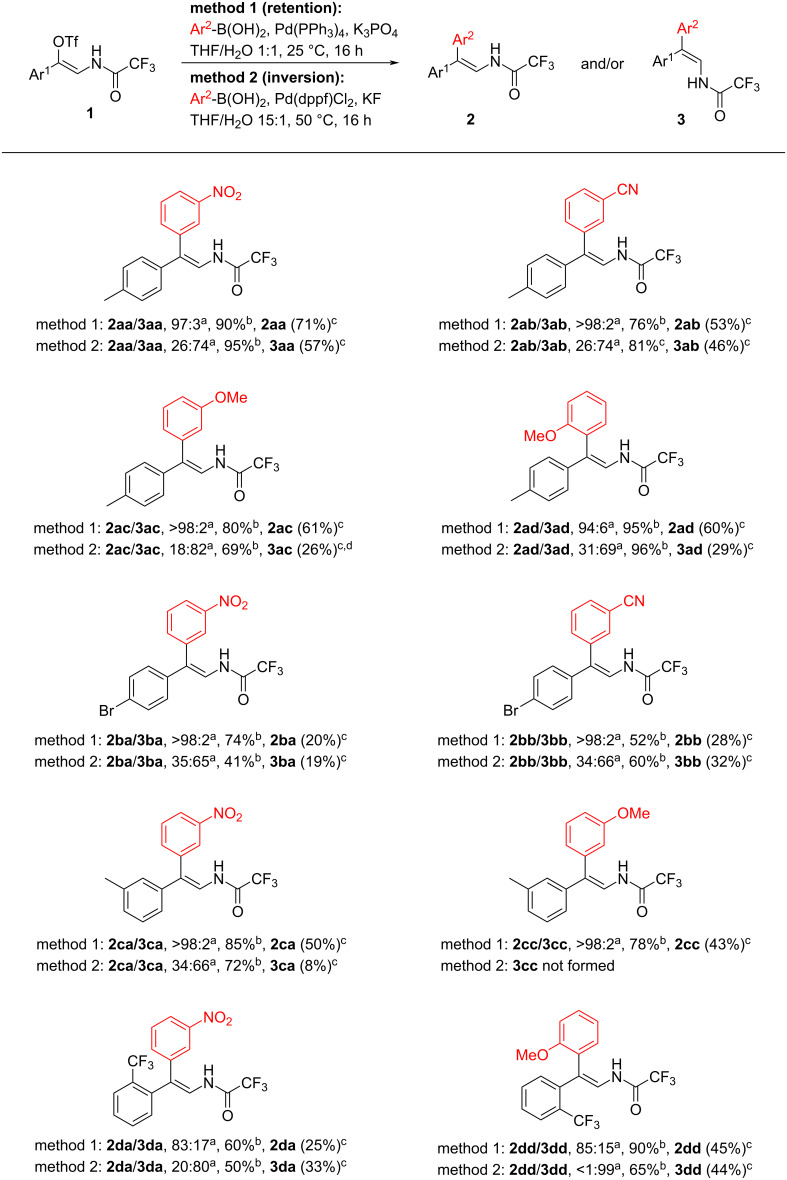
Substrate scope of the Suzuki coupling leading to enamides **2** and **3**. ^a^Ratio determined by ^19^F NMR; ^b^yield determined by ^19^F NMR using PhCF_3_ as an internal standard; ^c^isolated yield; ^d^isolated as a mixture of isomers *E*/*Z* = 64:36.

The optimized conditions were applied to the scope study. Substituted phenylboronic acids and vinyl triflates led to the formation of enamides **2** with high stereoselectivity and in good to high NMR yields (Scheme 2). However, isolated yields were found to be lower due to the decomposition of the formed enamides **2** during column chromatography on silica gel. A moderate loss of stereochemistry was observed only in cases of bulky *ortho*-substitution of either arylboronic acid or vinyl triflate (**2ad**, **2da**, and **2dd**). Alkylboronic acids were found to be unreactive even after prolonged reaction time (**1a** with *n*-hexylboronic acid, 60 h, rt).

Next, conditions for the formation of isomeric products were optimized. Screening the reaction conditions identified Pd(dppf)Cl_2_ (10 mol %), arylboronic acid (1.2 equiv), KF (2 equiv) in THF/H_2_O 15:1 at 50 °C as a system affording the best obtained ratio and yield in favor to product **3aa** (Table S2 in Supporting Information File 1). Subsequently the optimized conditions for preferential formation of **3** were used in the scope study and in all cases, isomeric products **3** formed preferentially (Scheme 2). The highest selectivity in favor to enamide **3** was noted in the Suzuki coupling of vinyl triflate **1d** bearing a 2-trifluoromethylphenyl group which gave enamides **3da** (**2da**/**3da**, 20:80) and **3dd** (**2dd**/**3dd**, <1:99). It is worth mentioning that the reaction of vinyl triflate **1c** with 3-methoxyphenylboronic acid led to full decomposition of the starting material. The stereochemistry of the double bond in compounds **2** and **3** was determined by 2D ROESY NMR analysis showing interaction between the alkenyl hydrogen and *ortho*-hydrogens on the aryl rings.

**Scheme 3:**
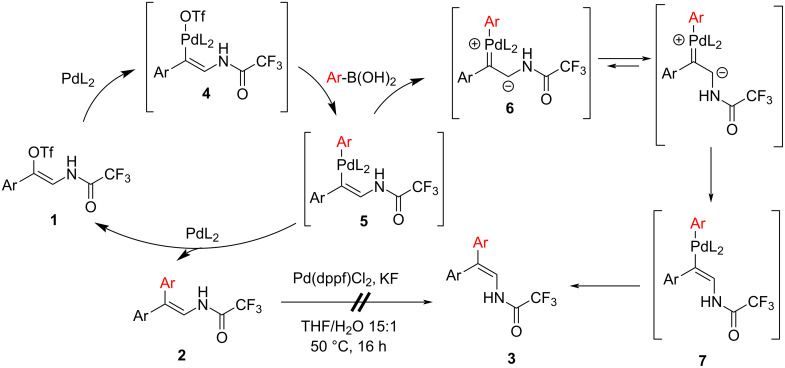
Proposed mechanisms for the formed Suzuki coupling retention products **2** and inversion products **3**.

Based on previously proposed mechanisms of isomerization in Suzuki cross-coupling reactions, we suggest the following explanation for the observed isomerization [25,29] (Scheme 3). In the first step, vinyl triflate undergoes oxidative addition to give complex **4**, which subsequently transmetalates with arylboronic acid to form palladium complex **5**. In the case of Pd(PPh_3_)_4_, reductive elimination occurs to give enamide **2**. However, using catalysts with very bulky ligands, such as Pd(dppf)Cl_2_ causes the tautomerization of complex **5** [30] to zwitterionic carbene **6** which can now isomerize through the C–C bond rotation to the thermodynamically more stable palladium complex **7**, followed by reductive elimination to enamide **3**. A possible isomerization of enamides **2** or **3** in the presence of a catalyst was ruled out because the treatment of **2ca** under conditions leading to inversion of the configuration did not affect the ratio between the resulting enamides.

## Conclusion

In conclusion, the stereoselective outcome of the Suzuki cross-coupling of vinyl triflates **1** with arylboronic acids was found to be catalyst dependent. The use of Pd(PPh_3_)_4_ led to the selective formation of enamides **2** with retention of configuration of the double bond. Reactions with other catalysts provided significant losses of stereoselectivity on the double bond. When Pd(dppf)Cl_2_ was used, enamides **3** with inversion of the configuration of the double bond were formed preferably. Both conditions were applied to a range of arylboronic acids and (*Z*)-β-enamido triflates.

## Supporting Information

File 1 Experimental part, optimization, compound characterization, and copies of NMR spectra.
