# Supplementary material for: Ligand-dependent stereoselective Suzuki–Miyaura cross-coupling reactions of β-enamido triflates
Source: Beilstein J Org Chem. 2021 Oct 29;17:2657–62. doi: 10.3762/bjoc.17.179 (PMC8561141; doi:10.3762/bjoc.17.179)
Supplement: File 1 — Experimental part, optimization, compound characterization, and copies of NMR spectra. [file Beilstein_J_Org_Chem-17-2657-s001.pdf]

BEILSTEIN JOURNAL OF ORGANIC CHEMISTRY

Supporting Information

for

Ligand-dependent stereoselective Suzuki–Miyaura cross-coupling reactions of β-enamido triflates

Tomáš Chvojka, Athanasios Markos, Svatava Voltrová, Radek Pohl and Petr Beier

Beilstein J. Org. Chem. **2021,** *17,* 2657–2662. doi:10.3762/bjoc.17.179

Experimental part, optimization, compound characterization, and copies of NMR spectra

License and Terms: This is a supporting information file under the terms of the Creative Commons Attribution License (https://creativecommons.org/licenses/by/4.0). Please note that the reuse, redistribution and reproduction in particular requires that the author(s) and source are credited and that individual graphics may be subject to special legal provisions.
The license is subject to the *Beilstein Journal of Organic Chemistry* terms and conditions: (https://www.beilstein-journals.org/bjoc/terms)

General information

All commercially available chemicals were used as received unless stated otherwise. Flash column chromatography was performed using silica gel 60 (0.040–0.063 mm). Automated flash column chromatography was performed on Teledyne ISCO CombiFlash Rf+ Lumen Automated Flash Chromatography System with UV–vis detection. ^{1}H, ^{13}C, and ^{19}F NMR spectra were measured on Bruker Avance III 400 MHz, Bruker Avance III 400 MHz Prodigy or Bruker Avance III 500 MHz spectrometers, at ambient temperature using 5 mm diameter NMR tubes. ^{1}H,^{1}H ROESY NMR spectra were recorded on a Bruker Avance III 500 MHz spectrometer. ^{13}C NMR spectra were proton decoupled. The chemical shift values (*δ*) are reported in ppm relative to internal Me_4Si (0 ppm for ^{1}H and ^{13}C NMR) or residual solvents and internal $CFCl_3$ (0 ppm for ^{19}F NMR). Coupling constants (*J*) are reported in hertz. Structural elucidation was aided by additional ^{1}H,^{1}H ROESY NMR. High resolution mass spectra (HRMS) were recorded on a Waters Micromass AutoSpec Ultima or Agilent 7890A GC coupled with Waters GCT Premier orthogonal acceleration time-of-flight detector using electron impact (EI) or chemical ionization (CI), on an LTQ Orbitrap XL using electrospray ionization (ESI), and on Q-Tof micro (Waters) quadrupole orthogonal acceleration time-of-flight tandem mass spectrometer using atmospheric-pressure chemical ionization (APCI). Reactions requiring heating were performed using a heating block.

Preparation of starting triazoles

Starting triazoles were prepared according to previously published procedures [1,2]. The previously unreported 1-(perfluoroethyl)-4-(*m*-tolyl)-1*H*-1,2,3-triazole was prepared in an analogous way and is characterized here.

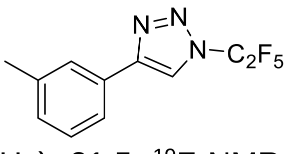

1-(Perfluoroethyl)-4-(*m*-tolyl)-1*H*-1,2,3-triazole: Yield 72%; white solid; ^{1}H NMR (400 MHz, $CDCl_3$) δ 8.16 (s, 1H), 7.72 (s, 1H), 7.66 (d, *J* = 7.7 Hz, 1H), 7.35 (t, *J* = 7.7 Hz, 1H), 7.23 (d, *J* = 7.7 Hz, 1H); ^{13}C NMR (101 MHz, $CDCl_3$) δ 149.0, 139.0, 130.3, 129.1, 128.5, 127.0, 123.4, 117.8, 117.2 (qt, *J* = 287.5, 41.3 Hz), 110.4 (tq, *J* = 270.7, 43.3 Hz), 21.5; ^{19}F NMR (376 MHz, $CDCl_3$) δ -84.4 (s, 3F), -99.2 (s, 2F); HRMS (ESI) *m/z*: $[M+H]^+$ Calcd for $C_{11}H_9F_5N_3$ 278.0711; found 278.0710.

Preparation of vinyl triflates **1a–d**

Vinyl triflates were prepared according to a previously published procedure [3]. Previously unreported vinyl triflates were prepared in an analogous way and are characterized here.

(*Z*)-1-(4-Bromophenyl)-2-(2,2,2-trifluoroacetamido)vinyl trifluoromethanesulfonate (**1b**):

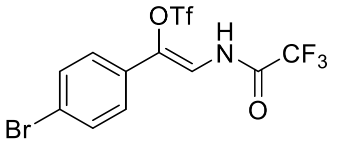

Yield 60%; white solid; ^{1}H NMR (401 MHz, $CDCl_3$) δ 8.27 (d, *J* = 10.8 Hz, 1H), 7.62–7.54 (m, 2H), 7.38–7.33 (m, 2H), 7.38–7.30 (m, 3H); ^{13}C NMR (101 MHz, $CDCl_3$) δ 154.5 (q, *J* = 39.8 Hz), 135.5, 132.6, 129.5, 126. 7, 124.9, 118.5 (q, *J* = 320.5 Hz), 115.3 (q, *J* = 287.2 Hz), 113.2; ^{19}F NMR (377 MHz, $CDCl_3$) δ -73.8 (s, 3F), -76.2 (s, 3F); HRMS (ESI) *m/z*: $[M-H]^-$ Calcd for $C_{11}H_5BrF_6NO_4S$ 439.9032; found 439.9031.

(*Z*)-1-(*m*-Tolyl)-2-(2,2,2-trifluoroacetamido)vinyl trifluoromethanesulfonate (**1c**): Yield

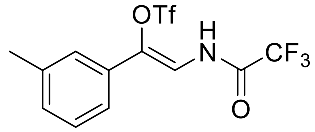

68%; white solid; ^{1}H NMR (401 MHz, $CDCl_3$) δ 8.25 (d, *J* = 10.7 Hz, 1H), 7.39–7.19 (m, 5H, signal overlapped with solvent), 2.40 (s, 3H); ^{13}C NMR (101 MHz, $CDCl_3$) δ 154.4 (q, *J* = 39.3 Hz), 139.2, 136.9, 131.4, 130.4, 129.2, 125.8, 122.5, 112.4, 118.5 (q, *J* = 328.8 Hz), 115.4 (q, *J* = 295.6 Hz), 21.6; ^{19}F NMR (377 MHz, $CDCl_3$) δ -73.9 (s, 3F), -76.3 (s, 3F); HRMS (ESI) *m/z*: $[M-H]^-$ Calcd for $C_{12}H_8F_6NO_4S$ 376.0084; found 376.0083.

(*Z*)-2-(2,2,2-Trifluoroacetamido)-1-(2-(trifluoromethyl)phenyl)vinyl trifluoromethanesulfo-

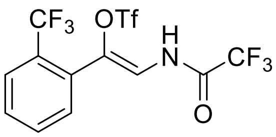

nate (**1d**): Yield 48%; white solid; 1H NMR (401 MHz, $CDCl_3$) δ 8.19 (d, *J* = 10.8 Hz, 1H), 7.81–7.77 (m, 1H), 7.66–7.63 (m, 2H), 7.59–7.56 (m, 1H), 7.10 (d, *J* = 10.1 Hz, 1H); ^{13}C NMR (101 MHz, $CDCl_3$) δ 154.6 (q, *J* = 39.6 Hz), 133. 6, 132.8, 132.4, 131.5, 129.9 (q, *J* = 31.4 Hz), 128.1 (q, *J* = 2.1 Hz), 127.3 (q, *J* = 5.1 Hz), 123.4 (q, *J* = 273.7 Hz),118.3 (q, *J* = 320.6 Hz), 116.8; 115.3 (q, *J* = 287.2 Hz); ^{19}F NMR (377 MHz, $CDCl_3$) δ -60.3 (q, *J* = 2.3 Hz, 3F), -74.4 (q, *J* = 2.2 Hz, 3F), -76.2 (s, 3F); HRMS (ESI) *m/z*: $[M-H]^-$ Calcd for $C_{12}H_5F_9NO_4S$ 429.9801; found 429.9800.

Table S1: Optimization of conditions for the retention of configuration of the double bond.

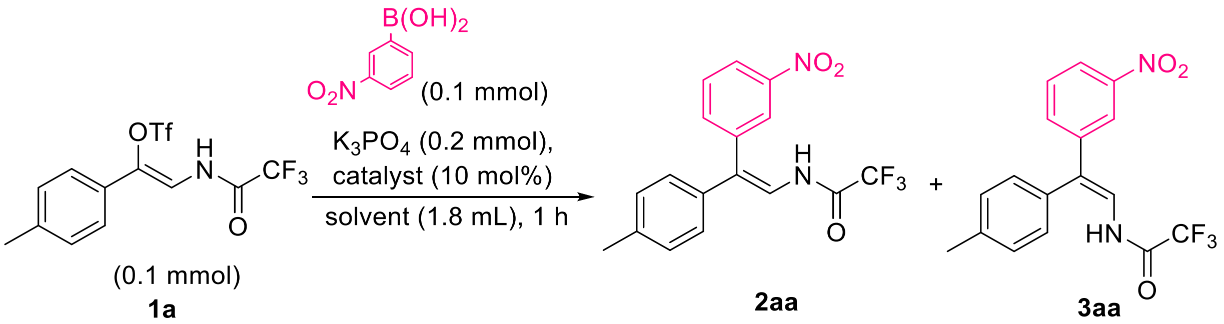

Entry	Catalyst	Solvent	**1**[a] (%)	**2aa**[a] (%)	**3aa**[a] (%)
1	$Pd(dppf)Cl_2$	DCE	30	n.d.	n.d.
2	$Pd(dppf)Cl_2$	DMF	29	n.d.	n.d.
3	$Pd(dppf)Cl_2$	THF/H_2O (1:1)	n.d.	43	54
4	$PdCl_2(PPh_3)_2$	DCE	51	7	12
5	$PdCl_2(PPh_3)_2$	DMF	39	n.d.	n.d.
6	$PdCl_2(PPh_3)_2$	THF/H_2O (1:1)	n.d.	88	12
7	$Pd(PPh_3)_4$	DCE	48%	3	n.d.
8	$Pd(PPh_3)_4$	DMF	27%	n.d.	n.d.
9	$Pd(PPh_3)_4$	THF/H_2O (1:1)[b]	n.d	**87**	3

[a] ^{19}F NMR yield using $PhCF_3$ as an internal standard. [b] Reaction time 16 h.

Table S2: Optimization of conditions for the inversion of the configuration of the double bond.

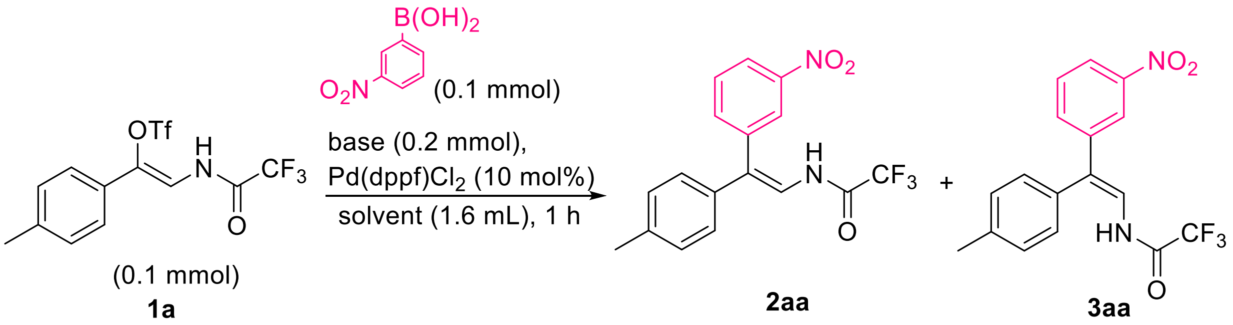

Base	Solvent	**1**[a] (%)	**2a**[a] (%)	**2b**[a] (%)
K_3PO_4	THF/H_2O (1:1)	3%	43%	54%
K_3PO_4	THF/H_2O (8:1)	n.d	20%	50%
K_3PO_4	THF/H_2O (15:1)[b,c]	n.d	24%	54%
K_3PO_4	THF[d]	n.d	50%	50%
K_3PO_4	$MeCN/H_2O$ (15:1)[b,c]	n.d	29%	34%
Na_2CO_3	THF/H_2O (8:1)[d]	12%	13%	23%
AcONa	THF/H_2O (8:1)[d]	n.d	18%	45%
KF	THF/H_2O (8:1)[d]	n.d	17%	51%
KF	THF/H_2O (15:1)[b,c]	n.d	25%	**70%**
KF	THF/H_2O (15:1)[b,c,e]	n.d	28%	72%
NaF	THF/H_2O (15:1)[b,c,e]	>90%	n.d	n.d
KF[f]	THF/H_2O (15:1)[b,c,e]	n.d	27%	67%
KF	THF/H_2O (2:1)[b,c,e]	n.d	25%	67%
KF	THF/H_2O (4:1)[b,c,e]	n.d	27%	73%
KF	THF/H_2O (7:1)[b,c,e]	n.d	28%	71%
KF	THF	29%	37%	27%
KF	1,4-dioxane[b,c]	73%	8%	15%
KF	$MeCN/H_2O$ (15:1)[b,c]	8%	8%	3%

[a] ^{19}F NMR yield using $PhCF_3$ as an internal standard. [b] Reaction temperature 50 °C. [c] Using 0.12 mmol of $Pd(dppf)Cl_2$. [d] Reaction time 16 h. [e] The reaction was performed in 3.2 mL of the solvent. [f] Using 0.4 mmol of KF.

General procedure for the synthesis of enamides **2**

Vinyl triflate **1** (0.2 mmol), boronic acid (0.2 mmol), and K_3PO_4 (85 mg; 0.4 mmol) were suspended in THF (1 mL) and water (1 mL). The reaction mixture was cooled to −30 °C. The tube was evacuated and back-filled with argon 3 times, heated to room temperature and tetrakis(triphenylphosphine)palladium (23 mg; 0.02 mmol) was added. The reaction mixture was stirred at 25 °C for 16 h. Et_2O and brine were added and the organic phase was separated, dried over $MgSO_4$, filtered, evaporated on silica gel, and the product was isolated by flash chromatography (cyclohexane/EtOAc from 100:0 to 50:50).

General procedure for the synthesis of enamides **3**

Vinyl triflate **1** (0.2 mmol), boronic acid (0.24 mmol), and KF (23 mg; 0.4 mmol) were suspended in THF (1.5 mL) and water (0.1 mL). The reaction mixture was cooled to −30 °C. The tube was evacuated and back-filled with argon 3 times, heated to room temperature and Pd(dppf)Cl_2 (15 mg; 0.02 mmol) was added. The reaction mixture was stirred at 50 °C for 16 h. Et_2O and brine were added and the organic phase was separated, dried over $MgSO_4$, filtered, evaporated on silica gel, and the product was isolated by flash chromatography(cyclohexane/EtOAc from 100:0 to 50:50).

Characterization of enamides **2** and **3**

((*Z*)-2,2,2-Trifluoro-*N*-(2-(3-nitrophenyl)-2-(*p*-tolyl)vinyl)acetamide (**2aa**): Isolated as *Z*/*E* = 96:4 mixture. Crude ratio (**2aa**/**3aa**, 97:3); ^{19}F NMR yield: 90%; Isolated yield: 71%; yellow solid; ^{1}H NMR (400 MHz, $CDCl_3$) δ 8.30 (ddd, *J* = 8.2, 2.3, 1.2 Hz, 1H), 8.16–8.10 (m, 1H), 7.74–7.62 (m, 3H), 7.38 (d, *J* = 10.8 Hz, 1H), 7.17–7.05 (m, 4H), 2.36 (s, 3H); ^{13}C NMR (101 MHz, $CDCl_3$) δ 154.4 (q, *J* = 38.3 Hz), 149.3, 138.8, 138.3, 135.6, 135.1, 130.8, 129.8, 127.8, 127.2, 124.8, 123.8, 117.6, 115.6 (q, *J* = 287.2 Hz), 21.3; ^{19}F NMR (376 MHz, $CDCl_3$) δ -76.2 (s, 3F); HRMS (ESI) *m/z*: $[M-H]^-$ Calcd for $C_{17}H_{12}F_3N_2O_3$ 349.0806; found 349.0803.

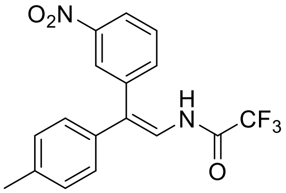

(*E*)-2,2,2-Trifluoro-*N*-(2-(3-nitrophenyl)-2-(*p*-tolyl)vinyl)acetamide (**3aa**): Crude ratio (**2aa**/**3aa**, 26:74); ^{19}F NMR yield: 95%; Isolated yield: 57%; yellow solid; ^{1}H NMR (500 MHz, $CDCl_3$) δ 8.13 (dd, *J* = 8.0, 2.1 Hz, 1H), 8.06 (t, *J* = 2.0 Hz, 1H), 7.93 (d, *J* = 10.4 Hz, 1H), 7.60 (d, *J* = 7.8 Hz, 1H), 7.49 (t, *J* = 8.0 Hz, 1H), 7.43 (d, *J* = 11.0 Hz, 1H), 7.34 (d, *J* = 7.8 Hz, 2H), 7.14 (d, *J* = 8.0 Hz, 2H), 2.44 (s, 3H); ^{13}C NMR (101 MHz, $CDCl_3$) δ 154.3 (q, *J* = 38.5 Hz), 148.6, 141.1, 139.7, 132.9, 131.7, 130.9, 129.6, 129.2, 128.0, 122.6, 122.0, 118.6, 115.6 (q, *J* = 287.3 Hz), 21.4; ^{19}F NMR (376 MHz, $CDCl_3$) δ -76.2 (s, 3F); HRMS (EI) *m/z*: $[M]^+$ Calcd for $C_{17}H_{13}F_3N_2O_3$ 350.0878; found 350.0875.

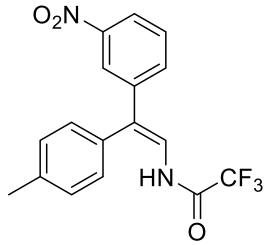

(*Z*)-*N*-(2-(3-Cyanophenyl)-2-(*p*-tolyl)vinyl)-2,2,2-trifluoroacetamide (**2ab**): Crude ratio (**2ab**/**3ab**, > 98:2); ^{19}F NMR yield: 76%; Isolated yield: 53%; yellow solid; ^{1}H NMR (401 MHz, $CDCl_3$) δ 7.74 (dt, *J* = 7.8, 1.4 Hz, 1H), 7.67–7.61 (m, 2H), 7.56 (dq, *J* = 4.6, 1.4 Hz, 2H), 7.36 (d, *J* = 11.0 Hz, 1H), 7.17–7.11 (m, 2H), 7.08–7.03 (m, 2H), 2.36 (s, 3H); ^{13}C NMR (101 MHz, $CDCl_3$) δ 154.3 (q, *J* = 38.5 Hz), 138.8, 137.9, 135.2, 133.9, 133.3, 132.4, 130.6, 129.7, 127.9, 127.2, 118.0, 117.4, 115.6 (q, *J* = 287.3 Hz), 114.3, 21.3; ^{19}F NMR (377 MHz, $CDCl_3$) δ -76.1 (s, 3F); HRMS (ESI) *m/z*: $[M-H]^-$ Calcd for $C_{18}H_{12}F_3N_2O$ 329.0907; found 329.0902.

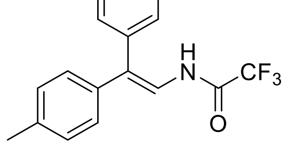

(*E*)-*N*-(2-(3-Cyanophenyl)-2-(*p*-tolyl)vinyl)-2,2,2-trifluoroacetamide (**3ab**): Crude ratio (**2ab**/**3ab**, 26:74); ^{19}F NMR yield: 81%; Isolated yield: 46%; pink solid; ^{1}H NMR (401 MHz, $CDCl_3$) δ 7.91 (d, *J* = 11.0 Hz, 1H), 7.59–7.51 (m, 2H), 7.47–7.30 (m, 5H), 7.15–7.07 (m, 2H), 2.44 (s, 3H); ^{13}C NMR (101 MHz, $CDCl_3$) δ 154.4 (q, *J* = 38.4 Hz), 140.6, 139.7, 131.8, 131.3, 131.1, 131.0, 130.9, 129.6, 129.3, 128.0, 118.6, 118.3, 115.6 (q, *J* = 287.3 Hz), 113.0, 21.5; ^{19}F NMR (377 MHz, $CDCl_3$) δ -76.2 (s, 3F); HRMS (EI) *m/z*: $[M]^+$ Calcd for $C_{18}H_{13}F_3N_2O$ 330.0980; found 330.0982.

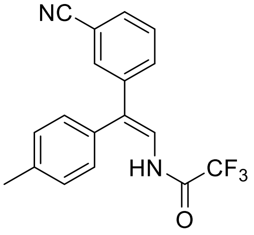

(*Z*)-2,2,2-Trifluoro-*N*-(2-(3-methoxyphenyl)-2-(*p*-tolyl)vinyl)acetamide (**2ac**): Crude ratio (**2ac**/**3ac**, > 98:2); ^{19}F NMR yield: 80%; Isolated yield: 61%; yellow solid; ^{1}H NMR (400 MHz, $CDCl_3$) δ 7.88 (d, *J* = 10.1 Hz, 1H), 7.41 (dd, *J* = 8.6, 7.4 Hz, 1H), 7.32 (d, *J* = 11.0 Hz, 1H), 7.17–7.10 (m, 4H), 6.97 (ddd, *J* = 8.4, 2.6, 1.0 Hz, 1H), 6.84 (ddd, *J* = 7.5, 1.6, 1.0 Hz, 1H), 6.75 (dd, *J* = 2.6, 1.5 Hz, 1H), 3.81 (s, 3H), 2.35 (s, 3H); ^{13}C NMR (101 MHz, $CDCl_3$) δ 160.6, 154.0 (q, *J* = 38.0 Hz), 138.1, 137.6, 135.9, 130.8, 130.0, 129.4, 127.2, 121.6, 116.1, 114.9, 115.7 (q, *J* = 287.3 Hz), 114.6, 55.5, 21.3; ^{19}F NMR (376 MHz, $CDCl_3$) δ -76.3 (s, 3F); HRMS (ESI) *m/z*: $[M+H]^+$ Calcd for $C_{18}H_{17}F_3NO_2$ 336.1206; found 336.1208.

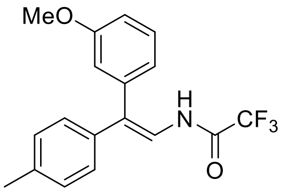

(*E*)-2,2,2-Trifluoro-*N*-(2-(3-methoxyphenyl)-2-(*p*-tolyl)vinyl)acetamide (**3ac**): Isolated as *Z*/*E* = 36:64 mixture. Crude ratio (**2ac**/**3ac**, 18:82); ^{19}F NMR yield: 69%; Isolated yield: 26%; yellow solid; ^{1}H NMR (401 MHz, $CDCl_3$) δ 8.76 – 7.84 (m, 1H **2ac**, + 1H **3ac**), 7.45 (dd, *J* = 8.4, 7.5 Hz, 1H, **2ac**), 7.34 (d, *J* = 11.0 Hz, 1H, **3ac**), 7.32 (d, *J* = 11.0 Hz, 1H, **2ac**), 7.33 – 7.26 (m, 2H, **3ac**), 7.23 (t, *J* = 8.0 Hz, 1H **3ac**), 7.18 – 7.10 (m, 4H **2ac**, + 2H **3ac**), 6.97 (ddd, *J* = 8.4, 2.6, 1.0 Hz, 1H, **2ac**), 6.87 – 6.72 (m, 1H **2ac** + 2H **3ac**), 6.75 – 6.75 (m, 1H **2ac**, + 1H **3ac**), 3.81 (s, 3H, **2ac**), 3.78 (s, 3H, **3ac**) 2.42 (s, 3H, **3ac**), 2.35 (s, 3H, **2ac**); ^{13}C NMR (101 MHz, $CDCl_3$) δ 160.6, 159.8, 154.1 (q, *J* = 38.2 Hz), 154.0 (q, *J* = 38.2 Hz), 140.6, 138.9, 138.1, 137.6, 135.9, 132.9, 130.4, 130.0, 129.6, 129.4 (2C), 127.1, 121.6, 119.9, 116.8, 116.0, 115.7 (q, *J* = 287.3 Hz), 114.9, 114.5, 113.3, 113.2, 55.5, 55.4, 21.4, 21.3; ^{19}F NMR (377 MHz, $CDCl_3$) δ -76.25 (s, 3F, **2ac**), 76.24 (s, 3F, **3ac**); HRMS (ESI) *m/z*: $[M+H]^+$ Calcd for $C_{18}H_{17}F_3NO_2$ 336.1206; found 336.1208.

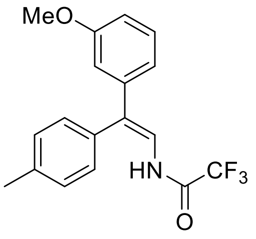

(*Z*)-2,2,2-Trifluoro-*N*-(2-(2-methoxyphenyl)-2-(*p*-tolyl)vinyl)acetamide (**2ad**): Crude ratio (**2ad**/**3ad**, 94:6); ^{19}F NMR yield: 95%; Isolated yield: 60%; yellow solid; ^{1}H NMR (400 MHz, $CDCl_3$) δ 7.89 (d, *J* = 10.6 Hz, 1H), 7.42 (ddd, *J* = 8.3, 7.2, 2.0 Hz, 1H), 7.30 (d, *J* = 10.6 Hz, 1H), 7.16–7.01 (m, 7H), 3.82 (s, 3H), 2.34 (s, 3H); ^{13}C NMR (101 MHz, $CDCl_3$) δ 156.5, 154.4 (q, *J* = 38.1 Hz), 137.7, 136.6, 132.0, 130.5, 129.3, 126.9, 126.5, 124.6, 121.7, 117.1, 115.9 (q, *J* = 287.4 Hz),112.2, 55.9, 21.3; ^{19}F NMR (376 MHz, $CDCl_3$) δ -76.4 (s, 3F); HRMS (ESI) *m/z*: $[M+H]^+$ Calcd for $C_{18}H_{17}F_3NO_2$ 336.1206; found 336.1208.

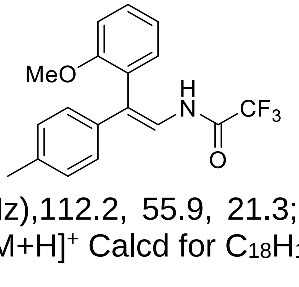

(*E*)-2,2,2-Trifluoro-*N*-(2-(2-methoxyphenyl)-2-(*p*-tolyl)vinyl)acetamide (**3ad**): Crude ratio (**2ad**/**3ad**, 31:69); ^{19}F NMR yield: 96%; Isolated yield: 29%; red solid; ^{1}H NMR (401 MHz, $CDCl_3$) δ 8.00 (d, *J* = 11.1 Hz, 1H), 7.36 (d, *J* = 11.1 Hz, 1H), 7.31–7.22 (m, 3H), 7.15–7.10 (m, 2H), 7.05 (dd, *J* = 7.8, 1.8 Hz, 1H), 6.92–6.86 (m, 2H), 3.71 (s, 3H), 2.39 (s, 3H); ^{13}C NMR (101 MHz, $CDCl_3$) δ 157.6, 154.0 (q, *J* = 37.8 Hz), 138.1, 134.4, 131.3, 130.1, 129.2, 128.7, 128.4, 127.0, 120.7, 119.1, 115.8 (q, *J* = 287.5 Hz), 111.5, 55.7, 21.4; ^{19}F NMR (377 MHz, $CDCl_3$) δ -76.3 (s, 3F); HRMS (EI) *m/z*: $[M]^+$ Calcd for $C_{18}H_{16}F_3NO_2$ 335.1133; found 335.1137.

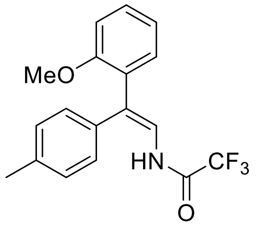

(*Z*)-*N*-(2-(4-Bromophenyl)-2-(3-nitrophenyl)vinyl)-2,2,2-trifluoroacetamide (**2ba**): Crude ratio (**2ba**/**3ba**, > 98:2); ^{19}F NMR yield: 74%; Isolated yield: 20%; yellow solid; ^{1}H NMR (401 MHz, $CDCl_3$) δ 8.31 (ddd, *J* = 8.2, 2.3, 1.1 Hz, 1H), 8.12 (t, *J* = 1.9 Hz, 1H), 7.76–7.61 (m, 3H), 7.49–7.45 (m, 2H), 7.41 (d, *J* = 11.1 Hz, 1H), 7.08–7.04 (m, 2H); ^{13}C NMR (101 MHz, $CDCl_3$) δ 154.5 (q, *J* = 38.7 Hz), 149.3, 137.6, 137.0, 135.5, 132.3, 131.1, 128.8, 126.7, 124.7, 124.1, 122.9, 118.7, 115.5 (q, *J* = 287.4 Hz); ^{19}F NMR (377 MHz, $CDCl_3$) δ -76.1 (s, 3F); HRMS (ESI) *m/z*: $[M-H]^-$ Calcd for $C_{16}H_9BrF_3N_2O_3$ 412.9754; found 412.97461.

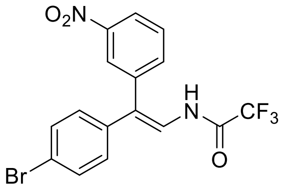

(*E*)-*N*-(2-(4-Bromophenyl)-2-(3-nitrophenyl)vinyl)-2,2,2-trifluoroacetamide (**3ba**): Crude ratio (**2ba**/**3ba**, 35:65); ^{19}F NMR yield: 41%; Isolated yield: 19%; yellow solid; ^{1}H NMR (401 MHz, $CDCl_3$) δ 8.16 (ddd, *J* = 7.9, 2.2, 1.3 Hz, 1H), 8.05 (t, *J* = 2.0 Hz, 1H), 7.81 (d, *J* = 11.1 Hz, 1H), 7.74–7.65 (m, 2H), 7.60–7.42 (m, 3H), 7.20–7.09 (m, 2H); ^{13}C NMR (101 MHz, $CDCl_3$) δ 154.5 (q, *J* = 38.7 Hz), 148.8, 140.4, 133.7, 133.57, 132.9, 131.1, 129.9, 126.7, 124.0, 123.0, 122.0, 119.3, 115.5 (q, *J* = 287.4 Hz); ^{19}F NMR (377 MHz, $CDCl_3$) δ -76.1 (s, 3F); HRMS (ESI) *m/z*: $[M-H]^-$ Calcd for $C_{16}H_9BrF_3N_2O_3$ 412.9754; found 412.9755.

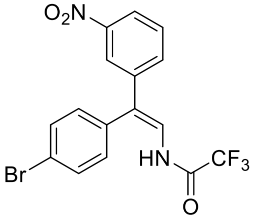

(*Z*)-*N*-(2-(4-Bromophenyl)-2-(3-cyanophenyl)vinyl)-2,2,2-trifluoroacetamide (**2bb**): Crude ratio (**2bb**/**3bb**, > 98:2); ^{19}F NMR yield: 52%; Isolated yield: 28%; yellow solid; ^{1}H NMR (401 MHz, $CDCl_3$) δ 7.75 (dt, *J* = 7.8, 1.4 Hz, 1H), 7.72–7.62 (m, 1H), 7.54 (dq, *J* = 5.1, 1.6 Hz, 2H), 7.49–7.43 (m, 2H), 7.38 (d, *J* = 10.9 Hz, 1H), 7.08–7.01 (m, 2H); ^{13}C NMR (101 MHz, $CDCl_3$) δ 154.4 (q, *J* = 38.6 Hz), 137.3, 137.0, 133.9, 133.2, 132.7, 132.2, 130.9, 128.8, 126.7, 122.8, 118.5, 117.9, 115.5 (q, *J* = 287.3 Hz), 114.5; ^{19}F NMR (377 MHz, $CDCl_3$) δ -76.1 (s, 3F); HRMS (APCI) *m/z*: $[M]^+$ Calcd for $C_{17}H_{10}BrF_3N_2O$ 393.99231; found 393.99182.

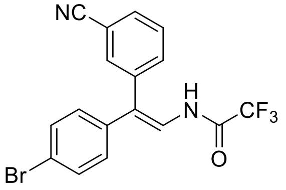

(*E*)-*N*-(2-(4-Bromophenyl)-2-(3-cyanophenyl)vinyl)-2,2,2-trifluoroacetamide (**3bb**): Crude ratio (**2bb**/**3bb**, 34:66); ^{19}F NMR yield: 60%; Isolated yield: 32%; white solid; ^{1}H NMR (401 MHz, $CDCl_3$) δ 7.79 (d, *J* = 10.9 Hz, 1H), 7.70–7.66 (m, 2H), 7.58 (dt, *J* = 7.4, 1.5 Hz, 1H), 7.52–7.37 (m, 4H), 7.14–7.11 (m, 2H); ^{13}C NMR (101 MHz, $CDCl_3$) δ 154.5 (d, *J* = 38.8 Hz), 139.9, 133.8, 133.5, 131.6, 131.1, 131.1, 130.9, 129.8, 126.7, 123.9, 118.9, 118.5, 115.5 (q, *J* = 287.2 Hz), 113.3; ^{19}F NMR (377 MHz, $CDCl_3$) δ -76.1 (s, 3F); HRMS (ESI) *m/z*: $[M-H]^-$ Calcd for $C_{17}H_9BrF_3N_2O$ 392.9856; found 392.9851.

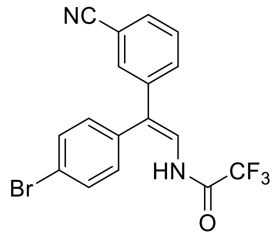

(*Z*)-2,2,2-Trifluoro-*N*-(2-(3-nitrophenyl)-2-(*m*-tolyl)vinyl)acetamide (**2ca**): Crude ratio (**2ca**/**3ca**, > 98:2); ^{19}F NMR yield: 85%; Isolated yield: 50%; orange solid; ^{1}H NMR (400 MHz, Chloroform-*d*) δ 8.30 (ddd, *J* = 8.1, 2.3, 1.2 Hz, 1H), 8.13 (dd, *J* = 2.1, 1.4 Hz, 1H), 7.76 – 7.67 (m, 2H), 7.65 (dt, *J* = 7.6, 1.3 Hz, 1H), 7.40 (d, *J* = 11.0 Hz, 1H), 7.23 (t, *J* = 7.6 Hz, 1H), 7.19 – 7.11 (m, 1H), 7.05 – 6.99 (m, 1H), 7.00 – 6.92 (m, 1H), 2.33 (s, 3H); ^{13}C NMR (101 MHz, $CDCl_3$) δ 154.4 (q, *J* = 38.4 Hz), 149.2, 138.8, 138.2, 138.0, 135.7, 130.8, 129.5, 128.9, 128.0, 127.9, 124.7, 124.6, 123.8, 118.1, 115.5 (q, *J* = 287.3 Hz), 21.5; ^{19}F NMR (377 MHz, $CDCl_3$) δ -76.1 (s, 3F); HRMS (APCI) *m/z*: $[M+H]^+$ Calcd for $C_{17}H_{14}F_3N_2O_3$ 351.0951; found 351.09485.

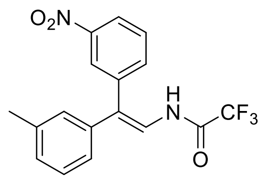

(*E*)-2,2,2-Trifluoro-*N*-(2-(3-nitrophenyl)-2-(*m*-tolyl)vinyl)acetamide (**3ca**): Crude ratio (**2ca**/**3ca**, 34:66); ^{19}F NMR yield: 72%; Isolated yield: 8%; yellow solid; ^{1}H NMR (401 MHz, $CDCl_3$) δ 8.14 (ddd, *J* = 8.2, 2.3, 1.1 Hz, 1H), 8.07 (t, *J* = 2.0 Hz, 1H), 7.91 (d, *J* = 11.0 Hz, 1H), 7.59 (ddd, *J* = 7.8, 1.8, 1.1 Hz, 1H), 7.52–7.41 (m, 3H), 7.30 (ddt, *J* = 7.7, 1.9, 1.0 Hz, 1H), 7.08–7.02 (m, 2H), 2.41 (s, 3H); ^{13}C NMR (101 MHz, $CDCl_3$) δ 154.4 (q, *J* = 38.4 Hz), 148.7, 141.0, 140.3, 134.8, 132.9, 130.4, 130.1, 129.9, 129.7, 128.1, 126.3, 122.7, 122.0, 118.7, 115.6 (q, *J* = 287.4 Hz), 21.6; ^{19}F NMR (377 MHz, $CDCl_3$) δ -76.3 (s, 3F); HRMS (ESI) *m/z*: $[M+H]^+$ Calcd for $C_{17}H_{12}F_3N_2O_3$ 349.08055; found 349.07998.

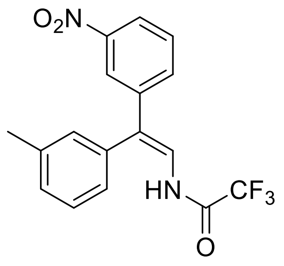

(*Z*)-2,2,2-Trifluoro-*N*-(2-(3-methoxyphenyl)-2-(*m*-tolyl)vinyl)acetamide (**2cc**): Crude ratio (**2cc**/**3cc**, > 98:2); ^{19}F NMR yield: 78%; Isolated yield: 43%; yellow solid; ^{1}H NMR (401 MHz, $CDCl_3$) δ 7.90 (d, *J* = 10.6 Hz, 1H), 7.42 (dd, *J* = 8.4, 7.3 Hz, 1H), 7.34 (d, *J* = 10.6 Hz, 1H), 7.23–7.17 (m, 1H), 7.13–7.08 (m, 2H), 7.03 (dtd, *J* = 7.6, 1.3, 0.7 Hz, 1H), 6.98 (ddd, *J* = 8.3, 2.6, 1.0 Hz, 1H), 6.85 (ddd, *J* = 7.6, 1.6, 1.0 Hz, 1H), 6.76 (dd, *J* = 2.7, 1.5 Hz, 1H), 3.81 (s, 3H), 2.33 (d, *J* = 0.6 Hz, 3H); ^{13}C NMR (101 MHz, $CDCl_3$) δ 160.6, 154.1 (q, *J* = 38.1 Hz), 138.7, 138.4, 137.5, 130.8, 130.2, 129.0, 128.6, 127.8, 124.6, 121.6, 116.6, 115.7 (q, *J* = 287.3 Hz),114.9, 114.6, 55.5, 21.6; ^{19}F NMR (377 MHz, $CDCl_3$) δ -76.3 (s, 3F); HRMS (ESI) *m/z*: $[M-H]^-$ Calcd for $C_{18}H_{15}F_3NO_2$ 334.10604; found 334.10548.

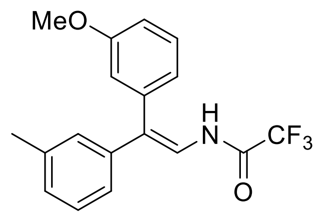

(E)-2,2,2-Trifluoro-N-(2-(3-nitrophenyl)-2-(2-(trifluoromethyl)phenyl)vinyl)acetamide

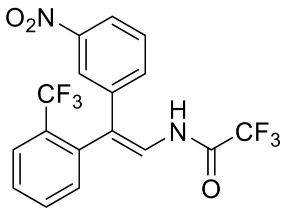

(**2da**): Crude ratio (**2da**/**3da**, 73:17); ^{19}F NMR yield: 60%; Isolated yield: 25%; orange solid; ^{1}H NMR (401 MHz, $CDCl_3$) δ 8.22 (dt, J = 7.3, 2.2 Hz, 1H), 8.06–8.00 (m, 2H), 7.74 (dd, J = 7.8, 1.5 Hz, 1H), 7.68–7.49 (m, 4H), 7.38–7.35 (m, 1H), 7.11 (d, J = 11.1 Hz, 1H); ^{13}C NMR (101 MHz, $CDCl_3$) δ 154.7 (q, J = 38.8 Hz), 149.1, 138.2, 137.0 (q, J = 1.9 Hz), 134.4, 132.8, 132.3, 130.7, 129.5 (q, J = 30.1 Hz), 129.1, 127.2 (q, J = 5.3 Hz), 124.4, 123.9 (q, J = 273.9 Hz), 123.6, 121.4 (q, J = 2.0 Hz), 115.5 (q, J = 287.5 Hz); ^{19}F NMR (377 MHz, $CDCl_3$) δ -58.2 (s, 3F), -76.1s (s, 3F); HRMS (ESI) m/z: $[M-H]^-$ Calcd for $C_{17}H_9F_6N_2O_3$ 403.0523; found 403.0514.

(Z)-2,2,2-Trifluoro-N-(2-(3-nitrophenyl)-2-(2-(trifluoromethyl)phenyl)vinyl)acetamide

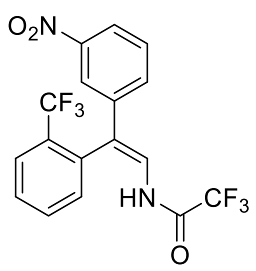

(**3da**): Crude ratio (**2da**/**3da**, 20:80); ^{19}F NMR yield: 50%; Isolated yield: 33%; yellow solid; ^{1}H NMR (401 MHz, $CDCl_3$) δ 8.12 (ddd, J = 7.8, 2.2, 1.4 Hz, 1H), 7.97 (t, J = 2.0 Hz, 1H), 7.95–7.89 (m, 1H), 7.80 (tdd, J = 7.5, 1.4, 0.7 Hz, 1H), 7.70 (tt, J = 7.8, 1.1 Hz, 1H), 7.64 (d, J = 11.1 Hz, 1H), 7.57–7.46 (m, 2H), 7.43–7.37 (m, 1H), 7.22 (d, J = 11.3 Hz, 1H); ^{13}C NMR (101 MHz, $CDCl_3$) δ 154.3 (q, J = 38.8 Hz), 148.7, 139.6, 133.7, 132.2, 132.2 (d, J = 2.1 Hz), 131.6, 130.3, 130.1 (q, J = 30.8 Hz), 129.8, 128.0 (q, J = 5.0 Hz), 124.8, 123.5 (q, J = 274.0 Hz),122.7, 120.8, 120.3, 115.4 (q, J = 287.2 Hz); ^{19}F NMR (377 MHz, $CDCl_3$) δ -61.2 (s, 3F), -76.4 (s, 3F); HRMS (ESI) m/z: $[M-H]^-$ Calcd for $C_{17}H_9F_6N_2O_3$ 403.0523; found 403.0513.

(Z)-2,2,2-Trifluoro-N-(2-(2-methoxyphenyl)-2-(2-(trifluoromethyl)phenyl)vinyl)acetamide

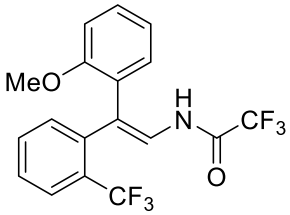

(**2dd**): Crude ratio (**2dd**/**3dd**, 85:15); ^{19}F NMR yield: 90%; Isolated yield: 45%; white solid; ^{1}H NMR (400 MHz, $CDCl_3$) δ 8.82 (d, J = 9.2 Hz, 1H), 7.68–7.64 (m, 1H), 7.55 (tdd, J = 7.6, 1.4, 0.7 Hz, 1H), 7.47–7.39 (m, 2H), 7.34 (ddd, J = 8.3, 6.8, 2.1 Hz, 1H), 7.08 (dd, J = 8.4, 1.2 Hz, 1H), 6.96–6.87 (m, 3H), 3.98 (s, 3H); ^{13}C NMR (101 MHz, Chloroform-d) δ 155.0, 154.4 (q, J = 37.4 Hz), 139.7 (q, J = 2.2 Hz), 133.0, 131.8, 131.6, 130.1, 129.2 (q, J = 30.1 Hz), 128.1, 126.9 (q, J = 5.1 Hz), 125.6, 124.05 (q, J = 274.1 Hz), 123.6, 121.6, 120.9, 116.0 (q, J = 287.6 Hz), 112.3, 56.1; ^{19}F NMR (376 MHz, $CDCl_3$) δ -59.0 (s, 3F), -76.5 (s, 3F); HRMS (APCI) m/z: $[M+H]^+$ Calcd for $C_{18}H_{14}F_6NO_2$ 390.0923; found 390.09216.

(E)-2,2,2-Trifluoro-N-(2-(2-methoxyphenyl)-2-(2-(trifluoromethyl)phenyl)vinyl)acetamide

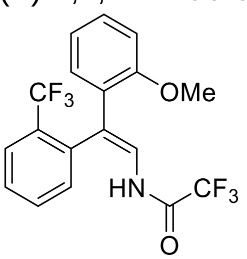

(**3dd**): Crude ratio (**2dd**/**3dd**, < 1:99); ^{19}F NMR yield: 65%; Isolated yield: 44%; yellow solid; ^{1}H NMR (401 MHz, $CDCl_3$) δ 7.88 (d, J = 11.3 Hz, 1H), 7.83 (d, J = 8.6 Hz, 1H), 7.73–7.55 (m, 2H), 7.38 (d, J = 7.1 Hz, 1H), 7.25–7.15 (m, 2H), 6.93 (dd, J = 8.3, 1.1 Hz, 1H), 6.86–6.75 (m, 2H), 3.82 (s, 3H); ^{13}C NMR (101 MHz, $CDCl_3$) δ 157.4, 153.8 (q, J = 38.0 Hz), 134.9 (q, J = 1.9 Hz), 132.9, 132.6, 130.6, 129.8 (q,

J = 30.7 Hz), 129.1, 128.9, 127.5 (q, *J* = 5.1 Hz), 126.4, 124.0, 123.7 (q, *J* = 274.1 Hz), 120.6, 115.6 (q, *J* = 287.3 Hz), 111.5, 55.6; ^{19}F NMR (377 MHz, $CDCl_3$) δ -61.1 (s, 3F), -76.5 (s, 3F); HRMS (ESI) *m/z*: $[M-H]^-$ Calcd for $C_{18}H_{12}F_6NO_2$ 388.0778; found 388.0771.

Configuration stability of **2ca**

Enamide **2ca** (33 mg; 0.09 mmol) and KF (10.9 mg; 0.19 mmol) were suspended in THF (0.7 mL) and water (0.035 mL). The reaction mixture was cooled to −30 °C. The tube was evacuated and back-filled with argon 3 times, heated to room temperature and Pd(dppf)Cl_2 (6.9 mg; 9 μmol) was added. The reaction mixture was stirred at 50 °C for 16 h. Finally, the crude mixture was analyzed by ^{19}F NMR and ^{1}H NMR spectroscopy, which did not show the formation of isomeric product **3ca**.

References

(1) Blastik, Z. E.; Voltrová, S.; Matoušek, V.; Jurásek, B.; Manley, D. W.; Klepetářová, B.; Beier, P. *Angew. Chemie Int. Ed.* **2017**, *56* (1), 346–349. doi:10.1002/anie.201609715

(2) Markos, A.; Janecký, L.; Klepetářová, B.; Pohl, R.; Beier, P. *Org. Lett.* **2021**. doi:10.1021/acs.orglett.1c01183

(3) Markos, A.; Voltrová, S.; Motornov, V.; Tichý, D.; Klepetářová, B.; Beier, P. *Chem. Eur. J.* **2019**, *25* (32), 7640–7644. doi:doi:10.1002/chem.201901632

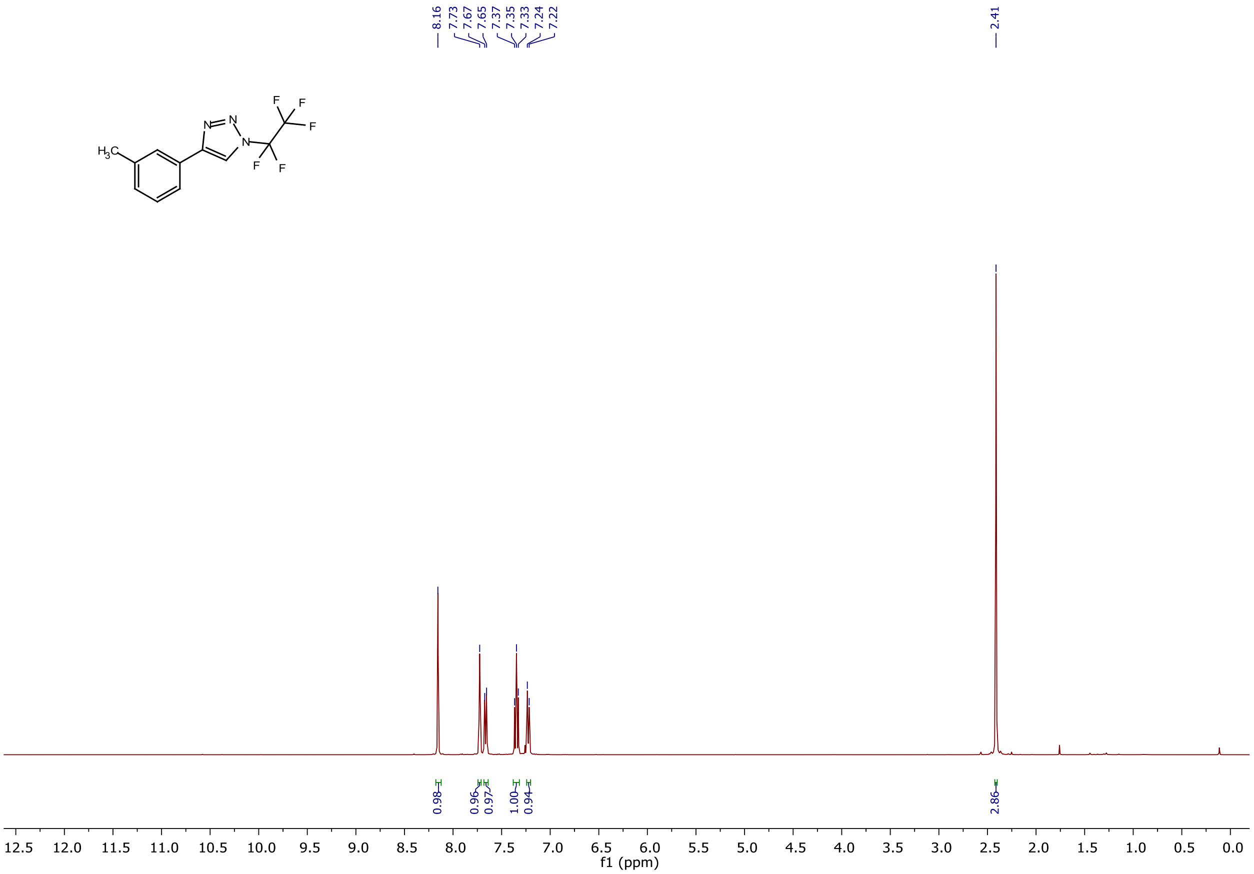

Figure SS1. ^{1}H NMR spectrum of 1-(perfluoroethyl)-4-(*m*-tolyl)-1*H*-1,2,3-triazole ($CDCl_3$, 400 MHz)

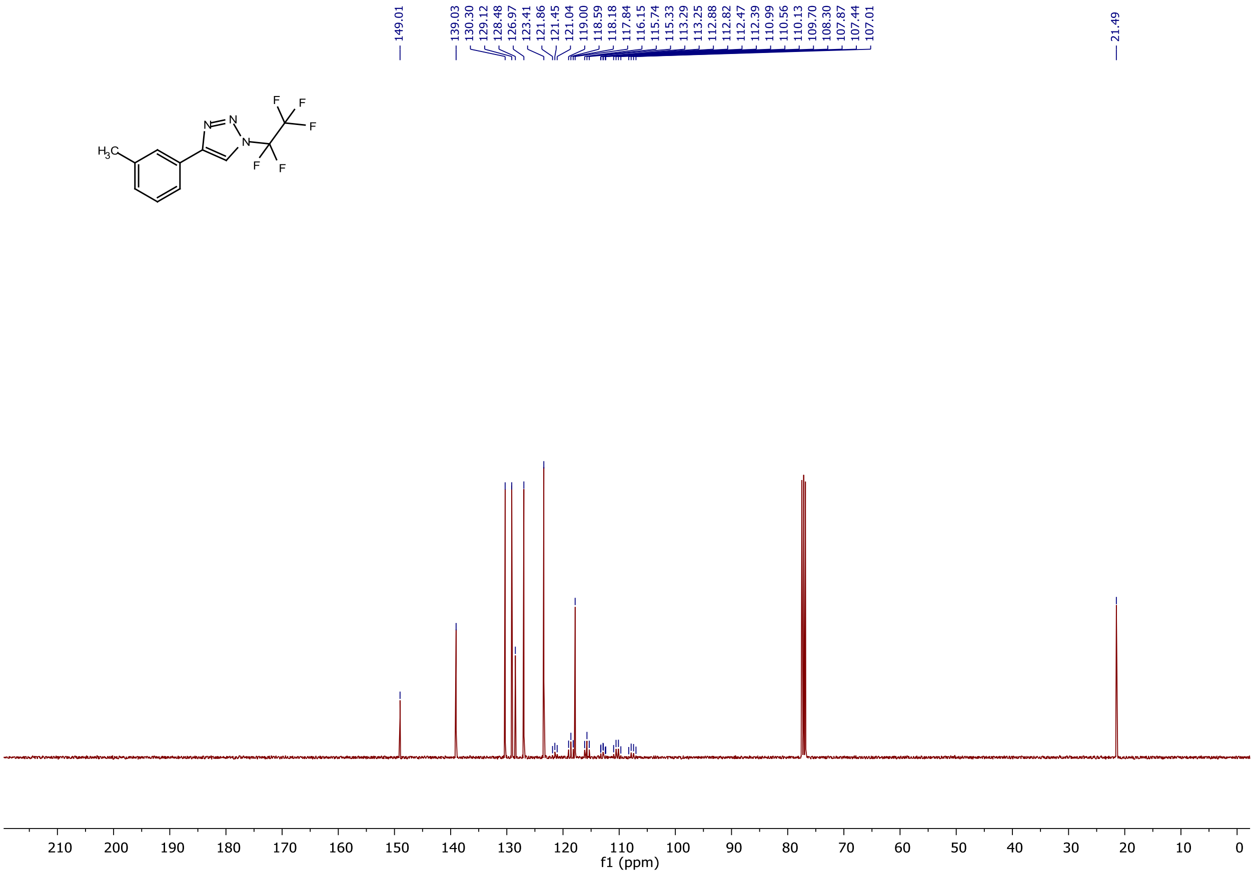

Figure SS2. ^{13}C NMR spectrum of 1-(perfluoroethyl)-4-(*m*-tolyl)-1*H*-1,2,3-triazole ($CDCl_3$, 101 MHz)

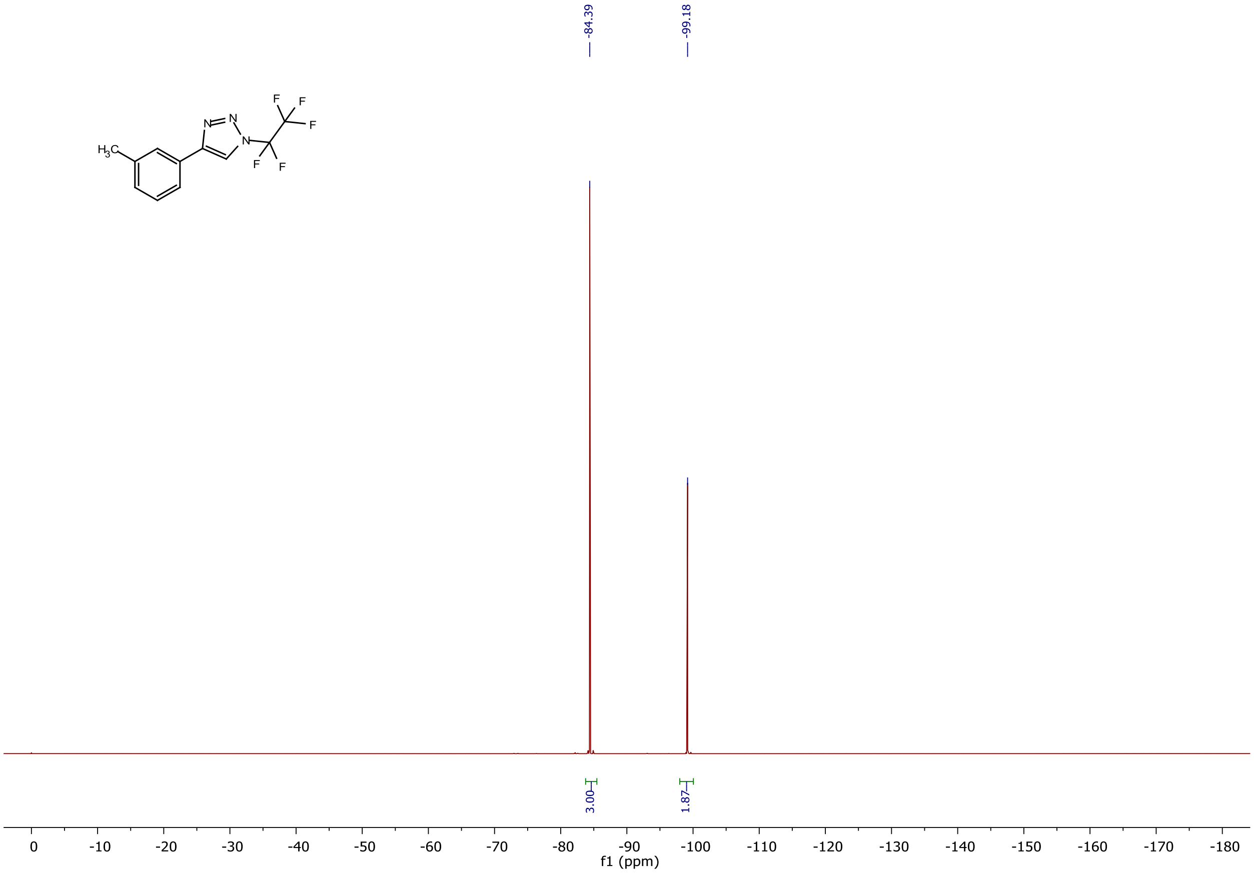

Figure SS3. ^{19}F NMR spectrum of 1-(perfluoroethyl)-4-(*m*-tolyl)-1*H*-1,2,3-triazole ($CDCl_3$, 377 MHz)

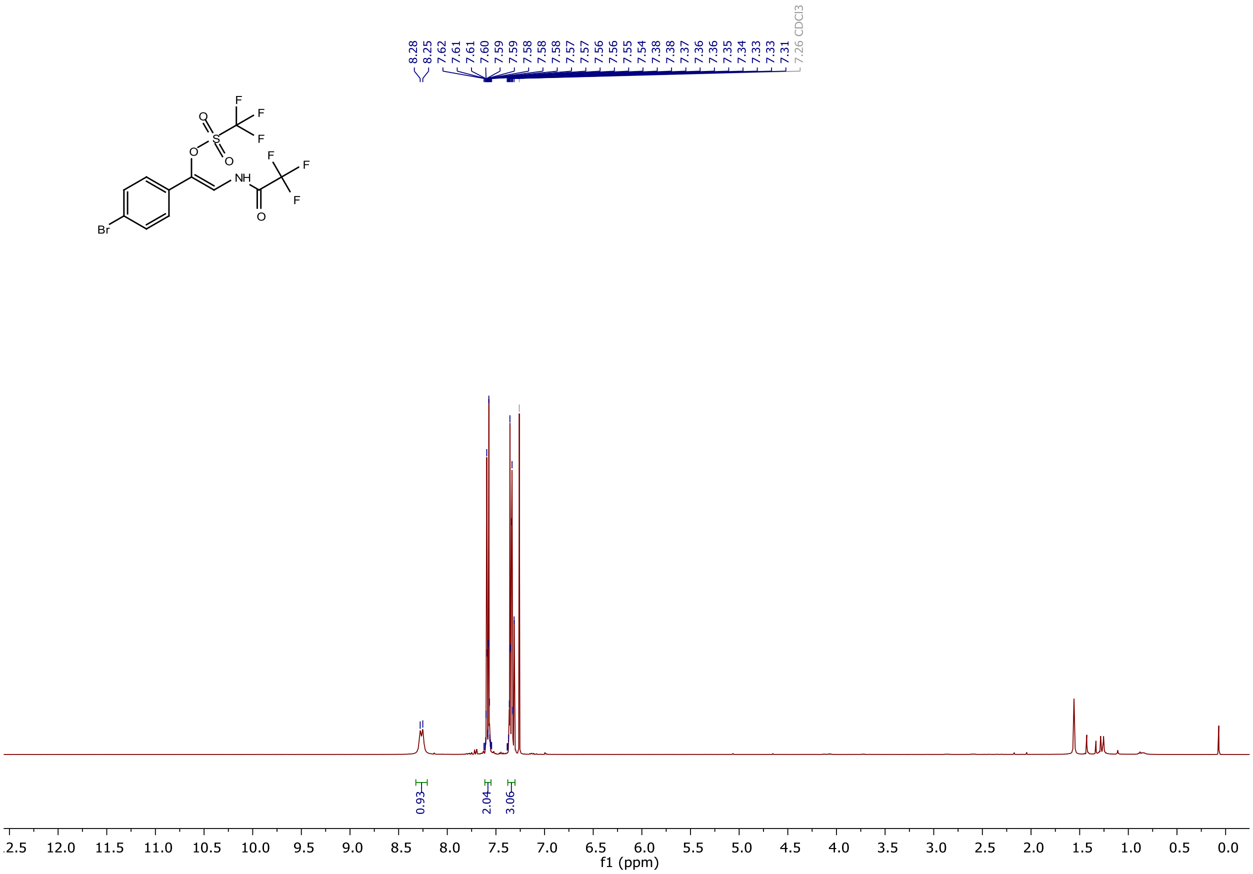

Figure SS4. ^{1}H NMR spectrum of **1b** ($CDCl_3$, 400 MHz)

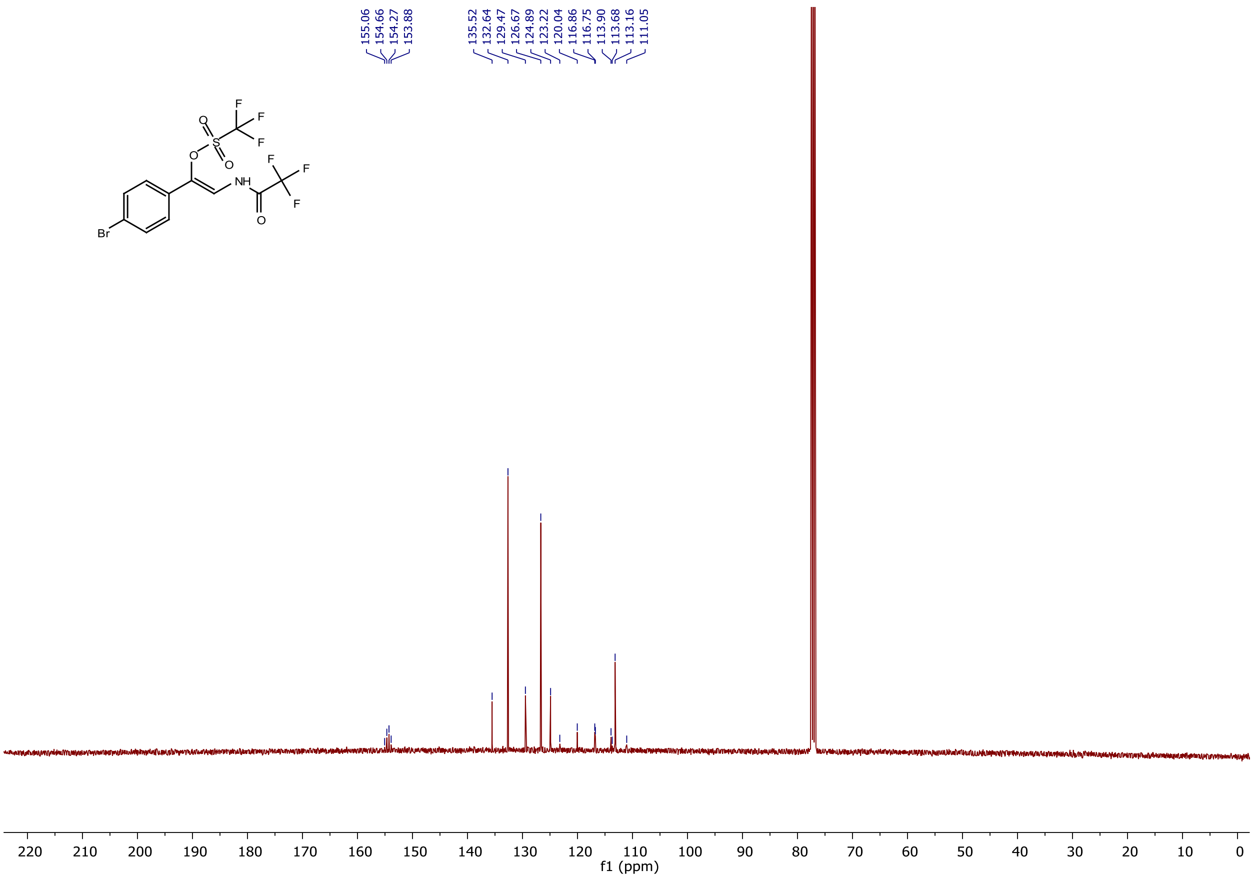

Figure SS5. ^{13}C NMR spectrum of **1b** ($CDCl_3$, 101 MHz)

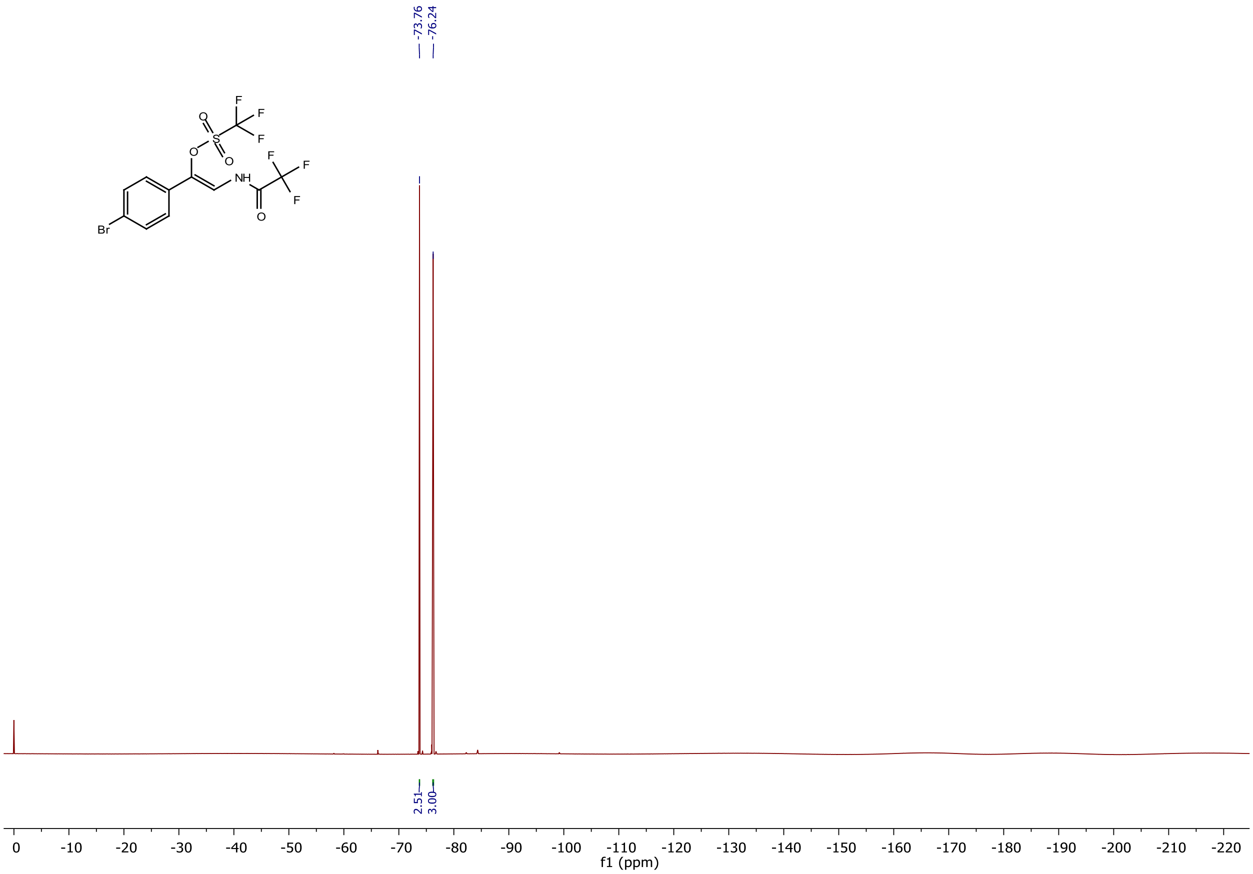

Figure SS6. ^{19}F NMR spectrum of **1b** ($CDCl_3$, 377 MHz)

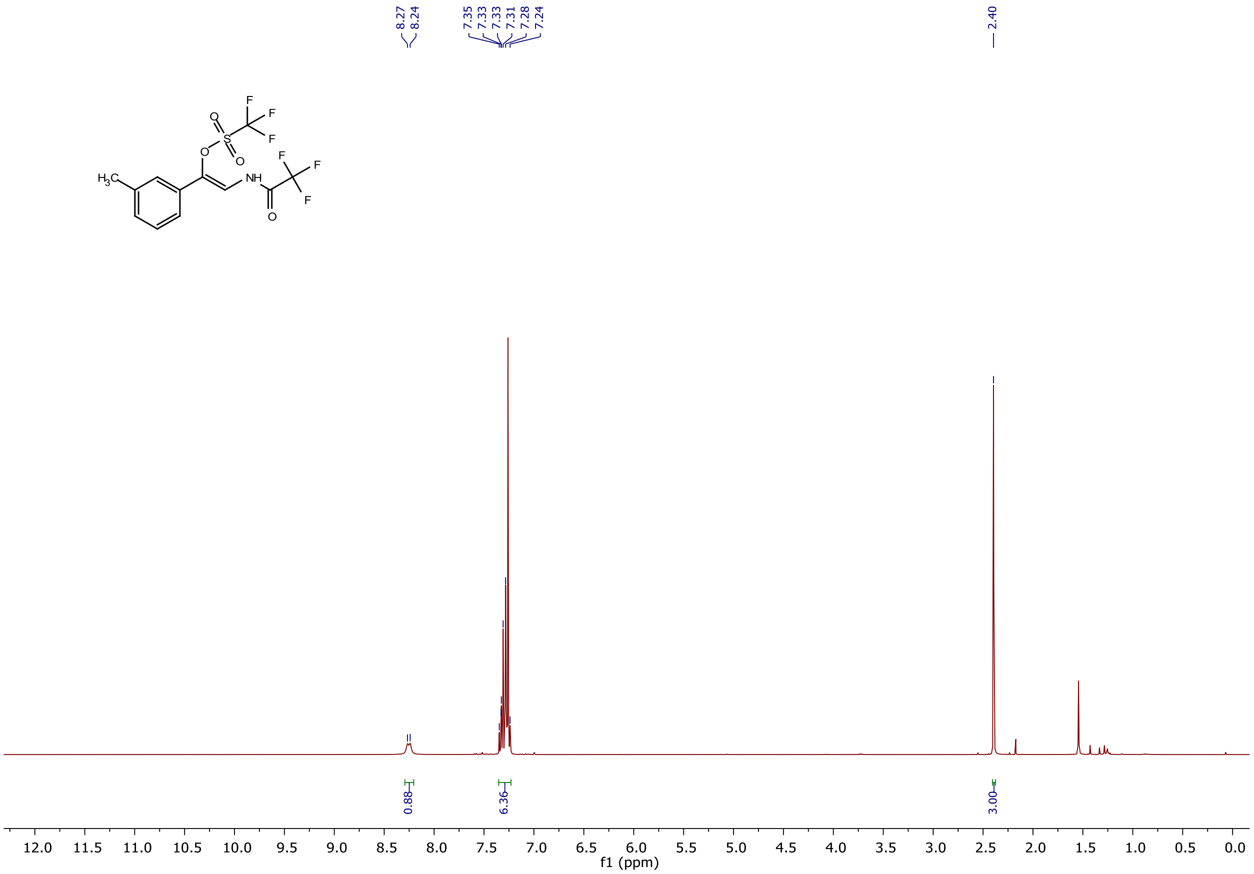

Figure S7. ^{1}H NMR spectrum of **1c** ($CDCl_3$, 400 MHz)

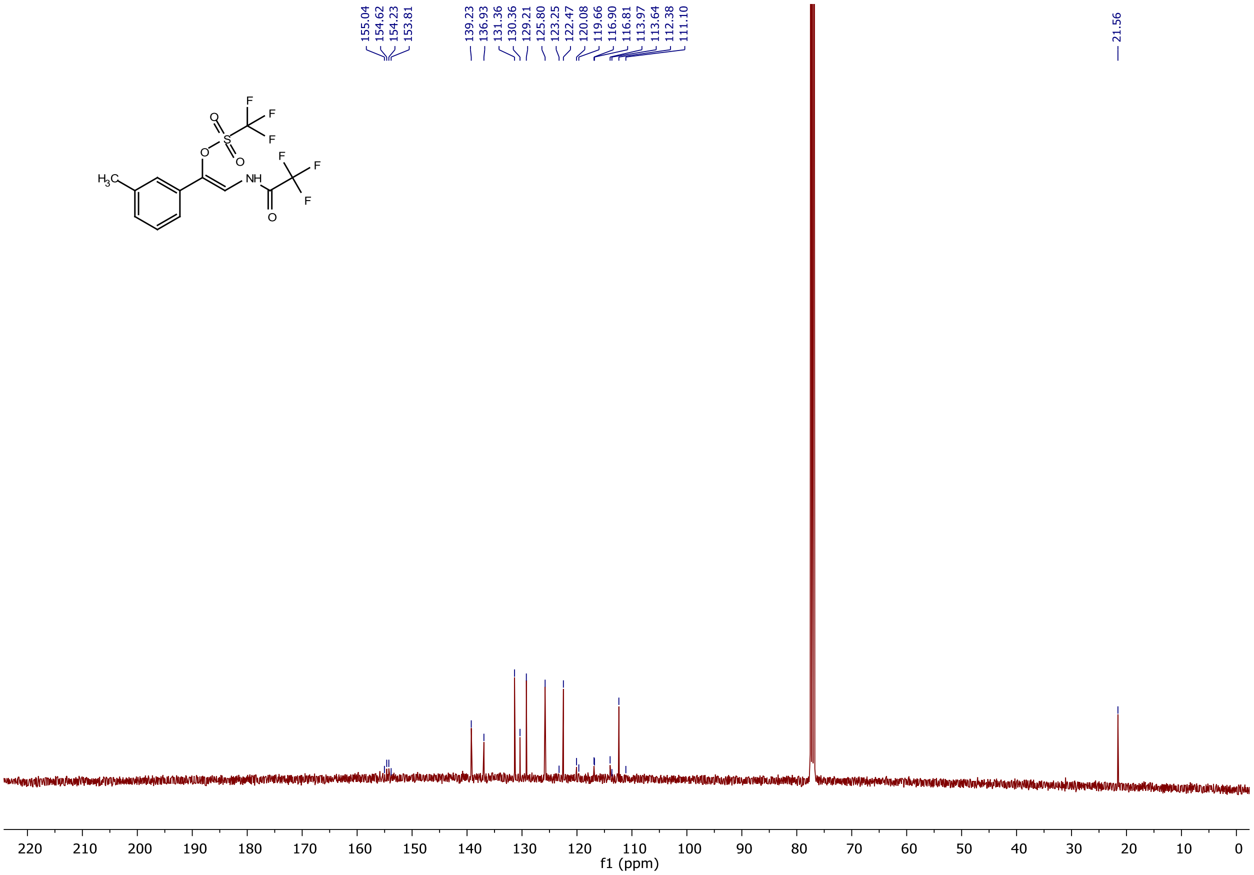

Figure S8. ^{13}C NMR spectrum of **1c** ($CDCl_3$, 101 MHz)

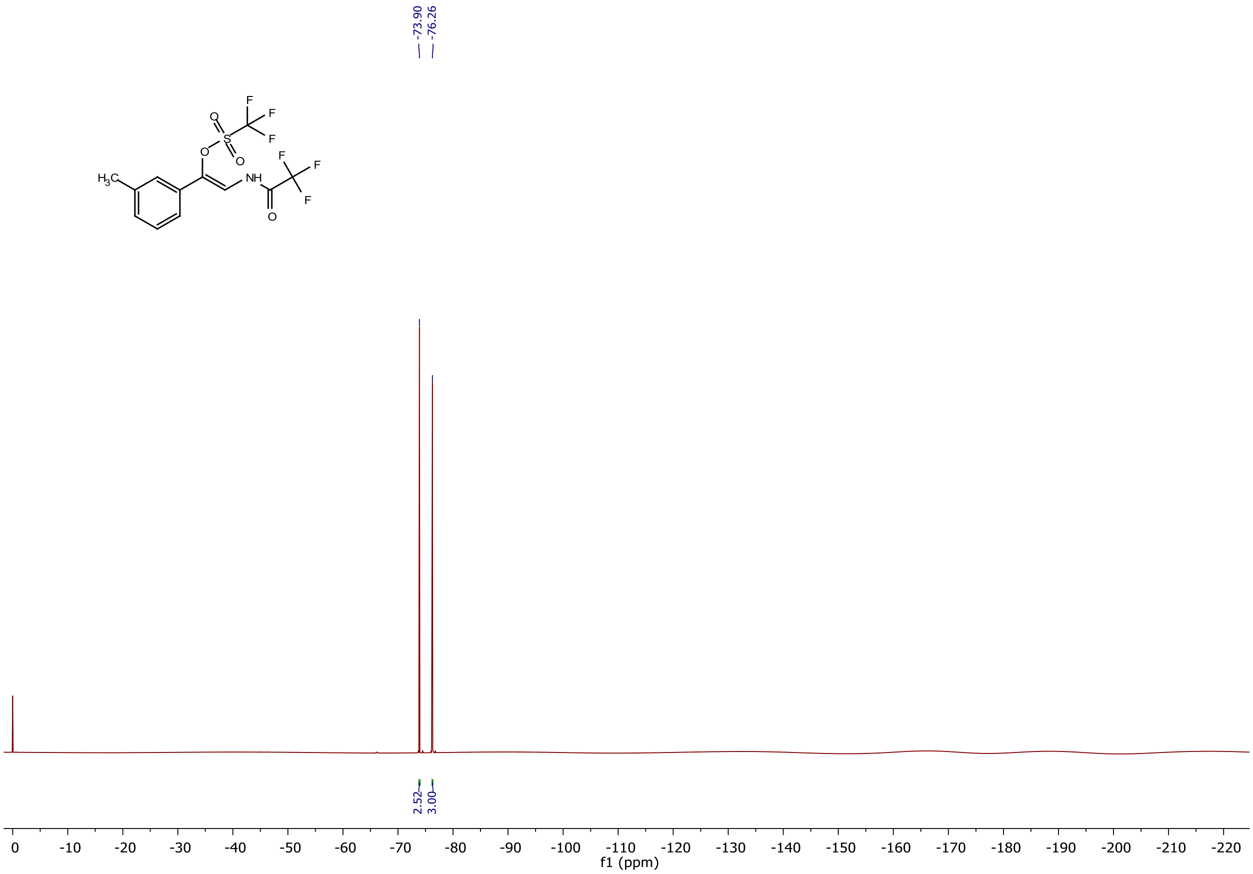

Figure S9. ^{19}F NMR spectrum of **1c** ($CDCl_3$, 377 MHz)

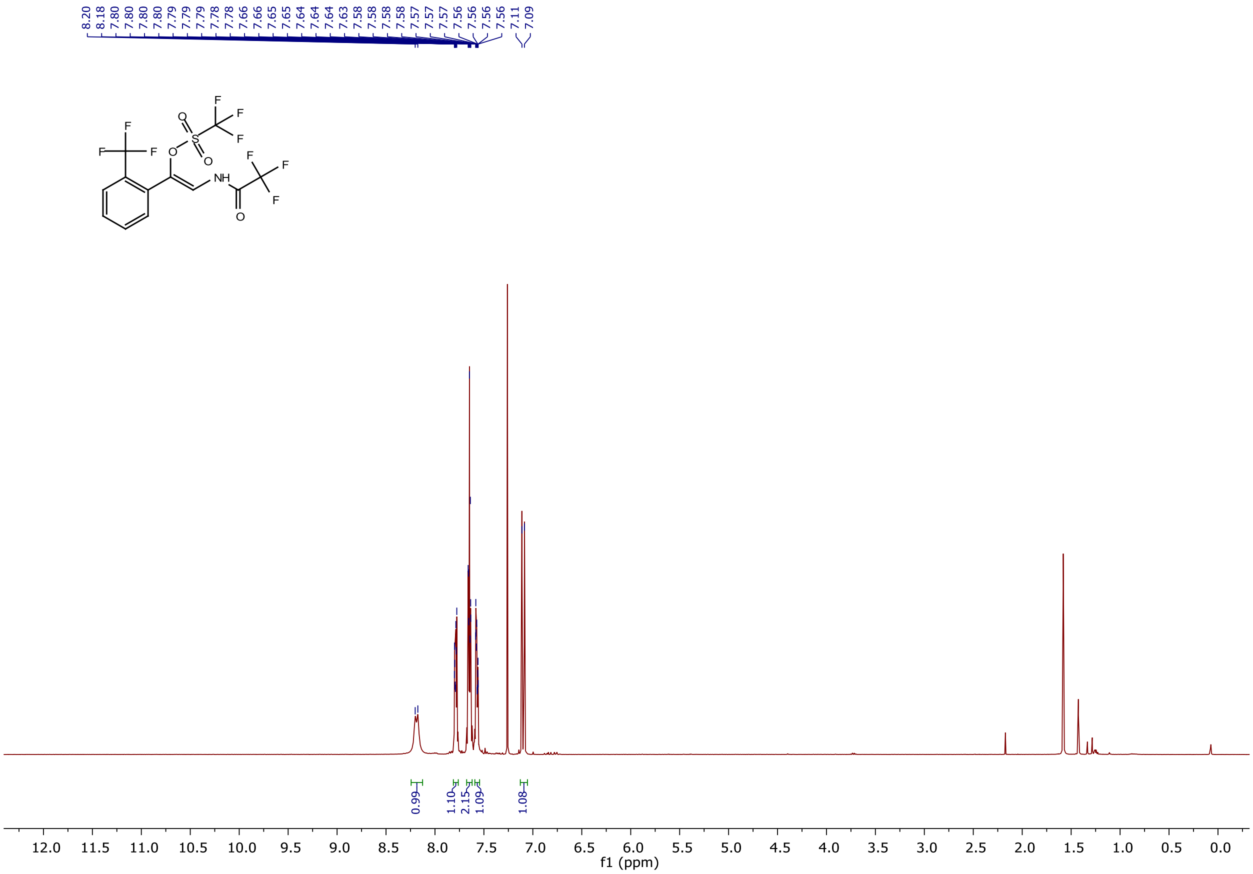

Figure S10. ^{1}H NMR spectrum of **1d** ($CDCl_3$, 400 MHz)

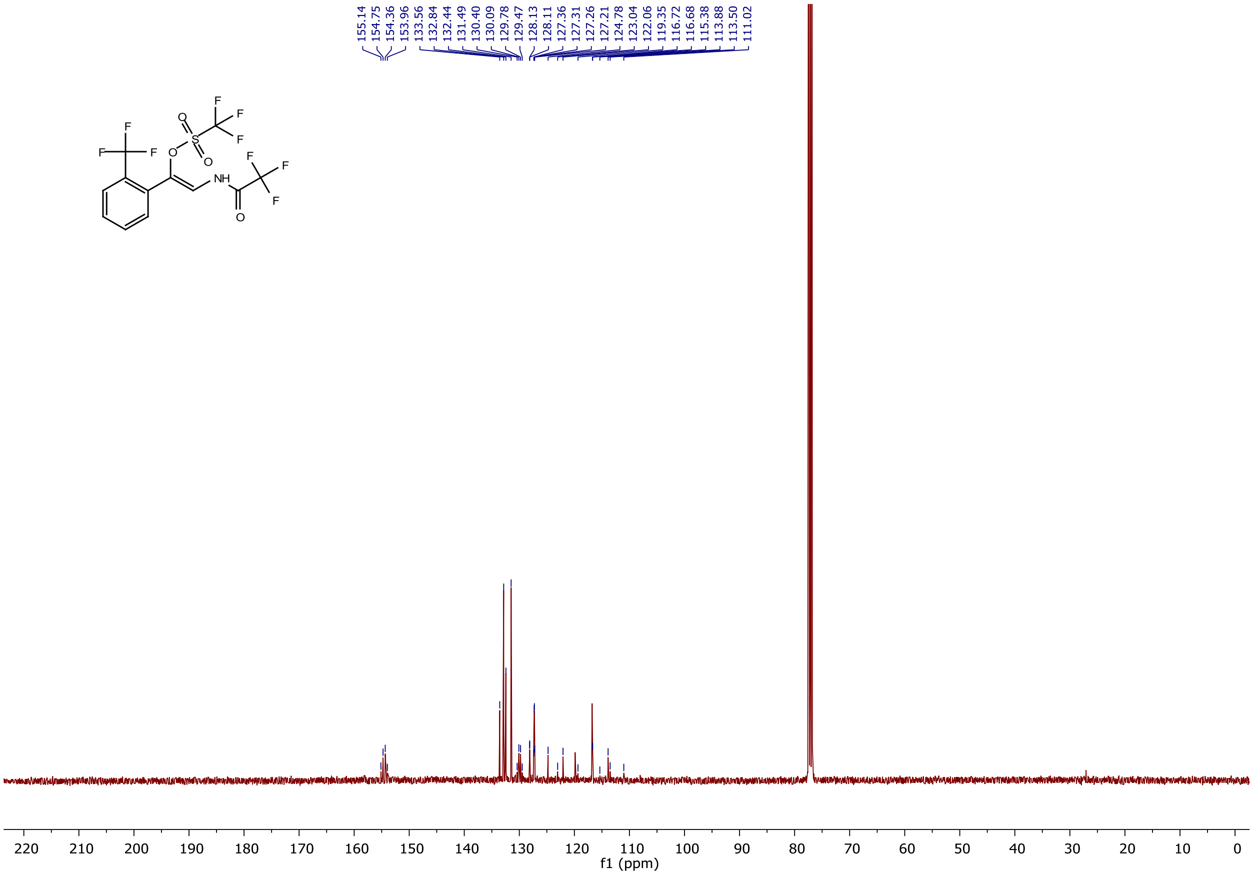

Figure S11. ^{13}C NMR spectrum of **1d** ($CDCl_3$, 101 MHz)

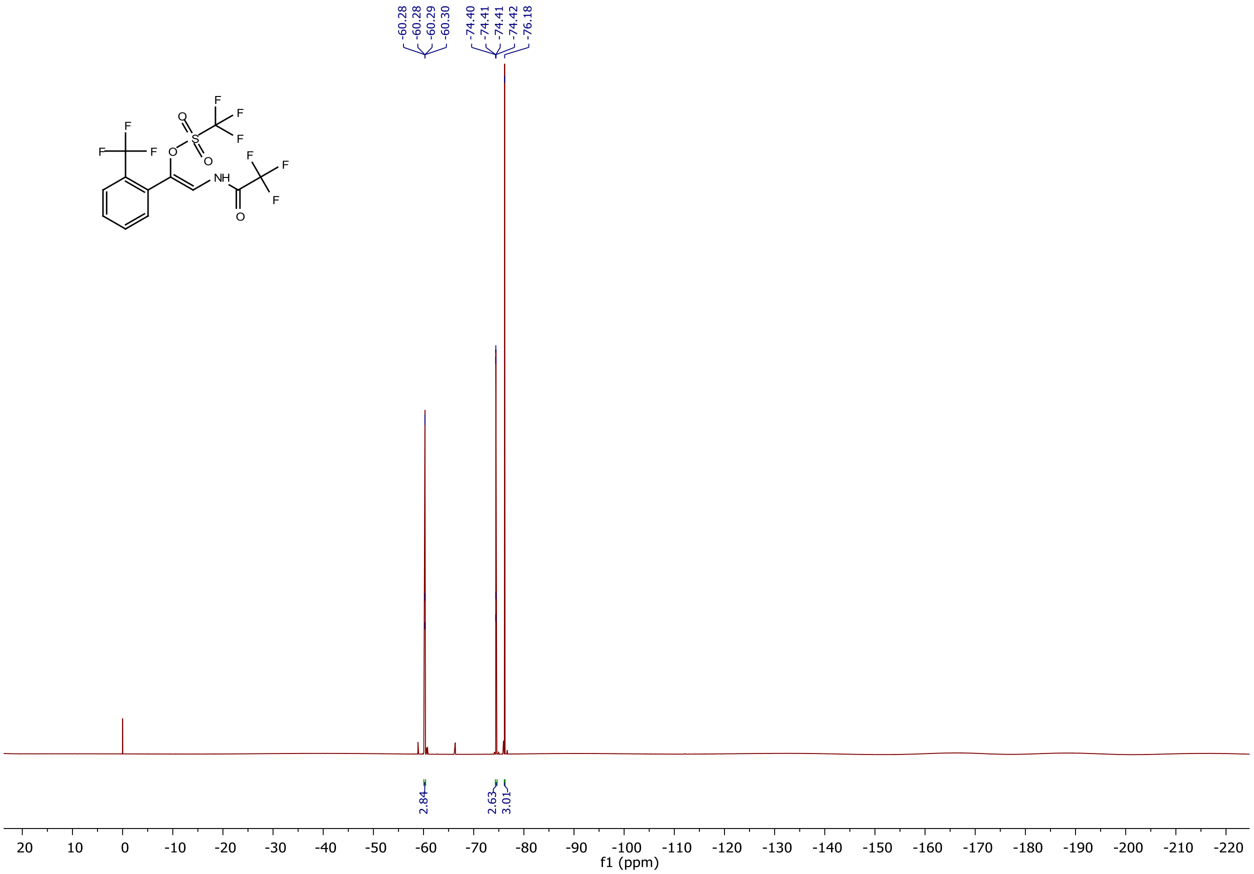

Figure S12. ^{19}F NMR spectrum of **1d** ($CDCl_3$, 377 MHz)

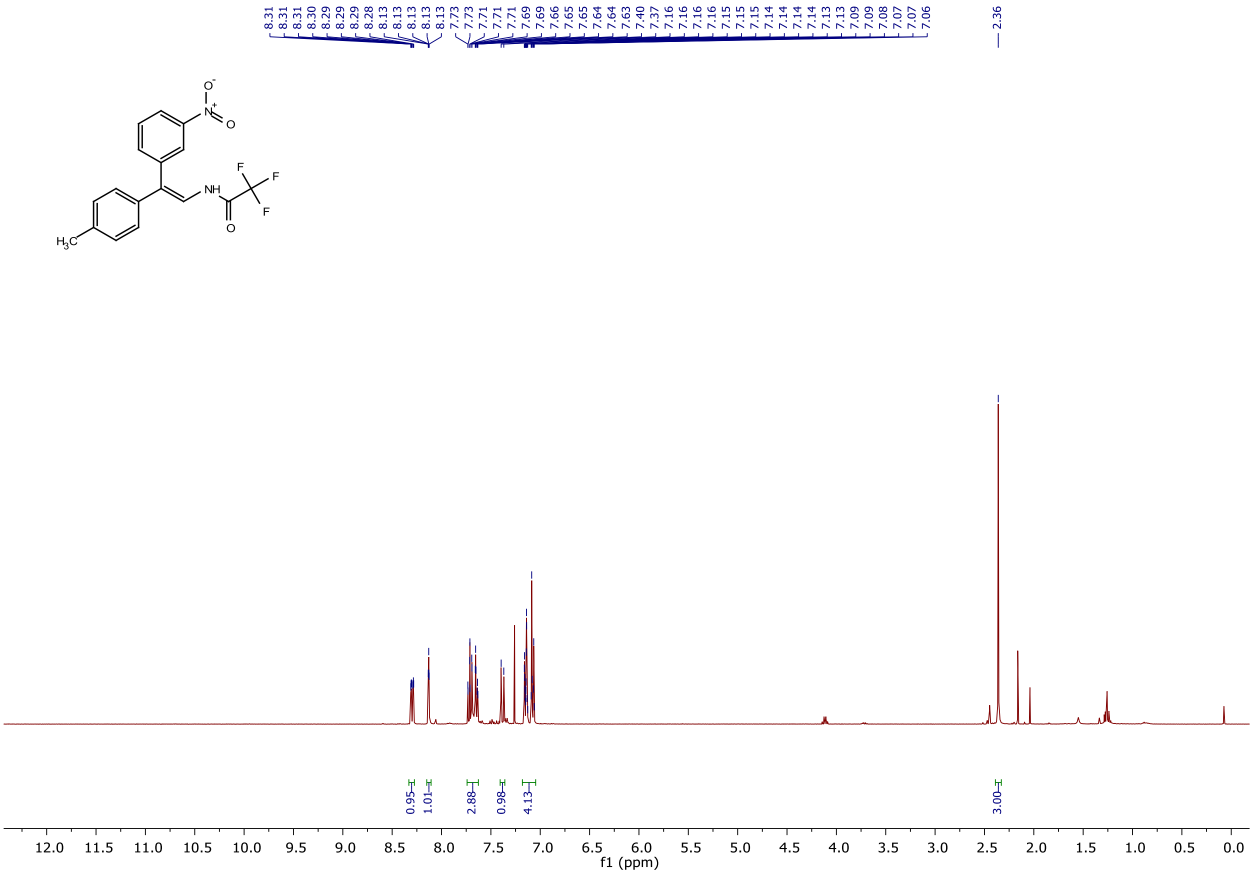

Figure S13. ^{1}H NMR spectrum of **2aa** ($CDCl_3$, 400 MHz)

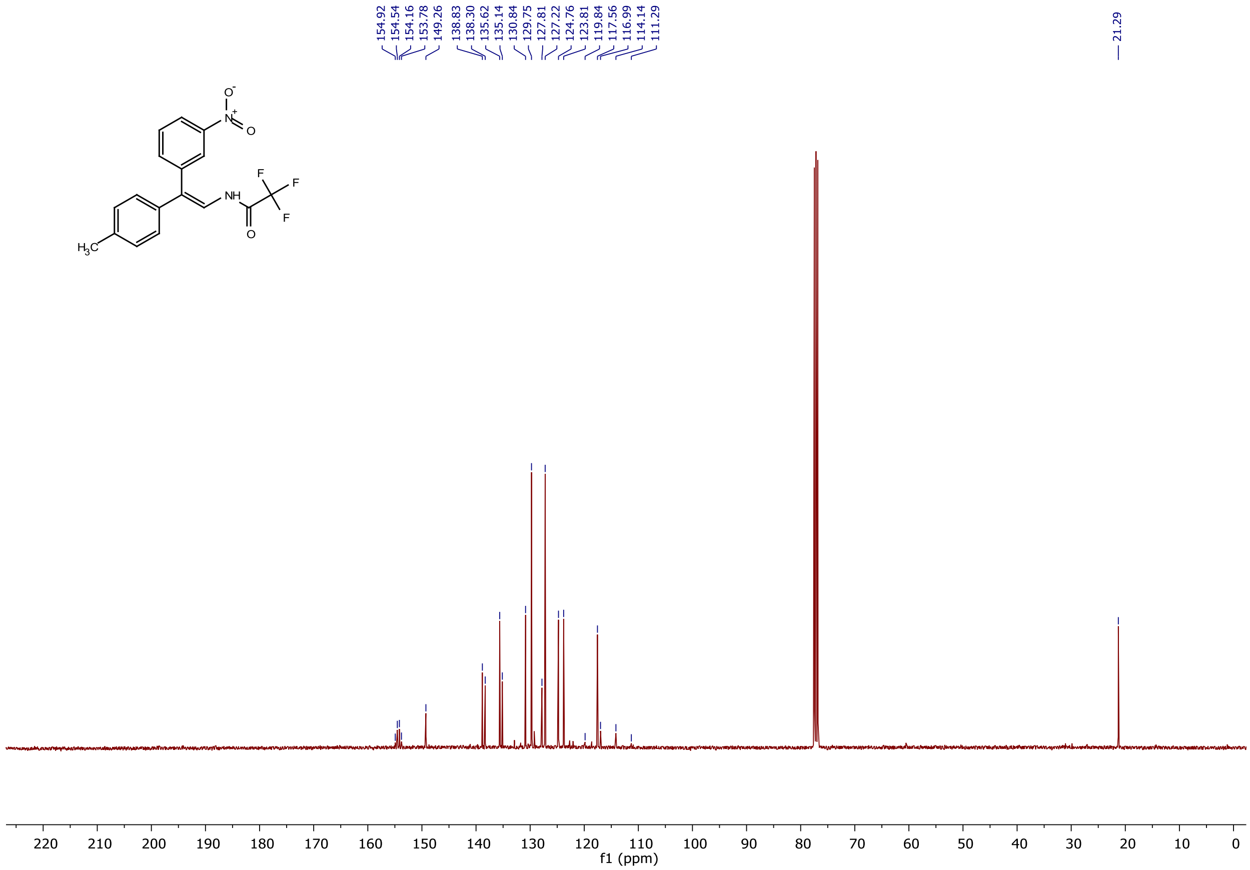

Figure S14. ^{13}C NMR spectrum of **2aa** ($CDCl_3$, 101 MHz)

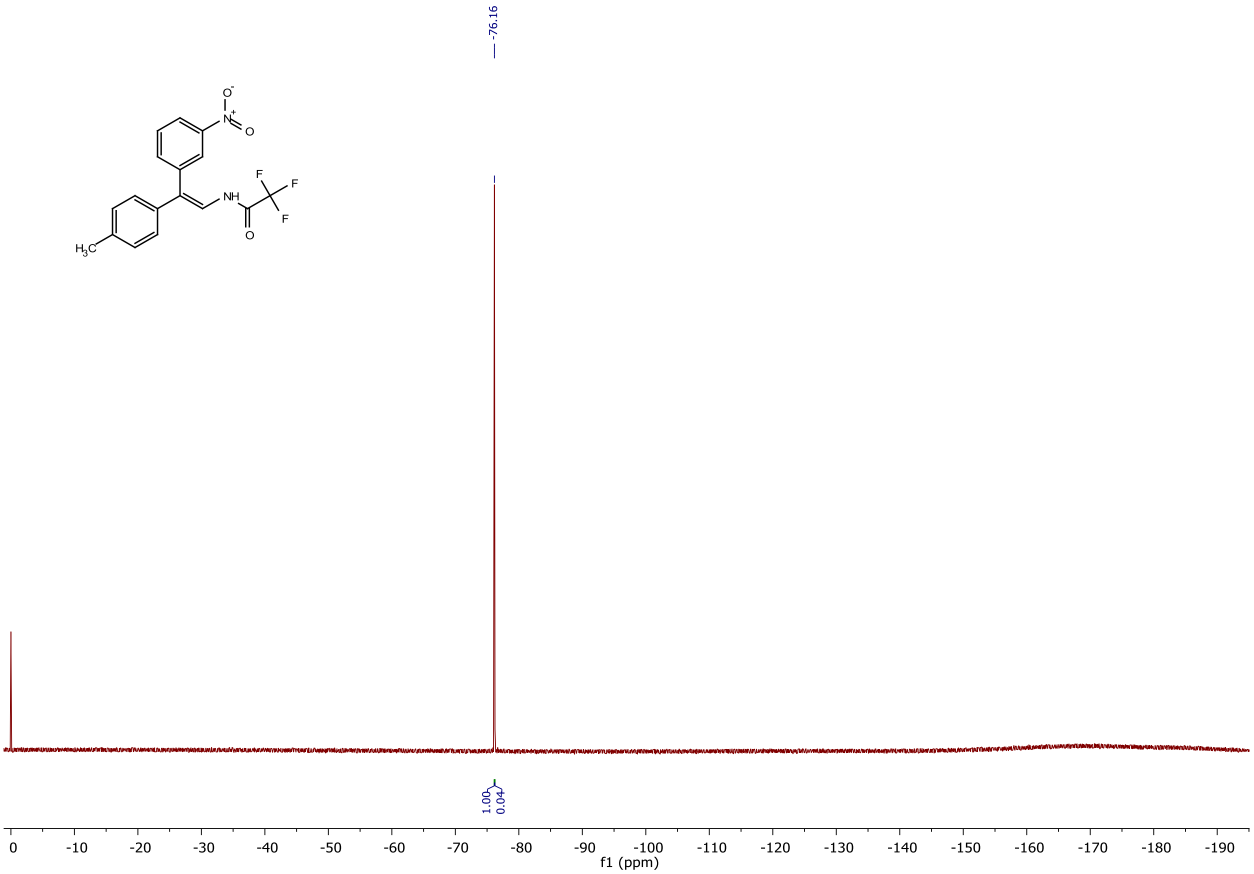

Figure S15. ^{19}F NMR spectrum of **2aa** ($CDCl_3$, 377 MHz)

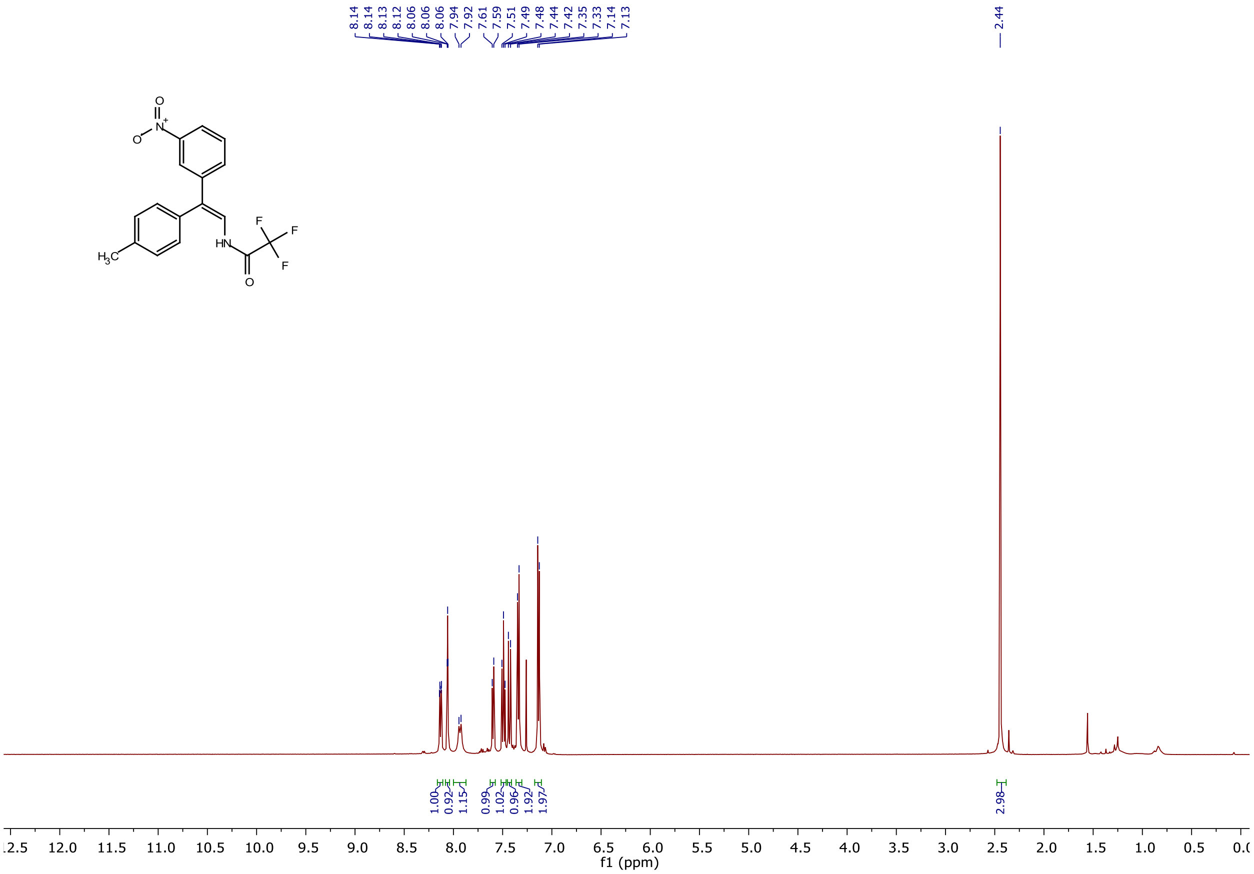

Figure S16. ^{1}H NMR spectrum of **3aa** ($CDCl_3$, 400 MHz)

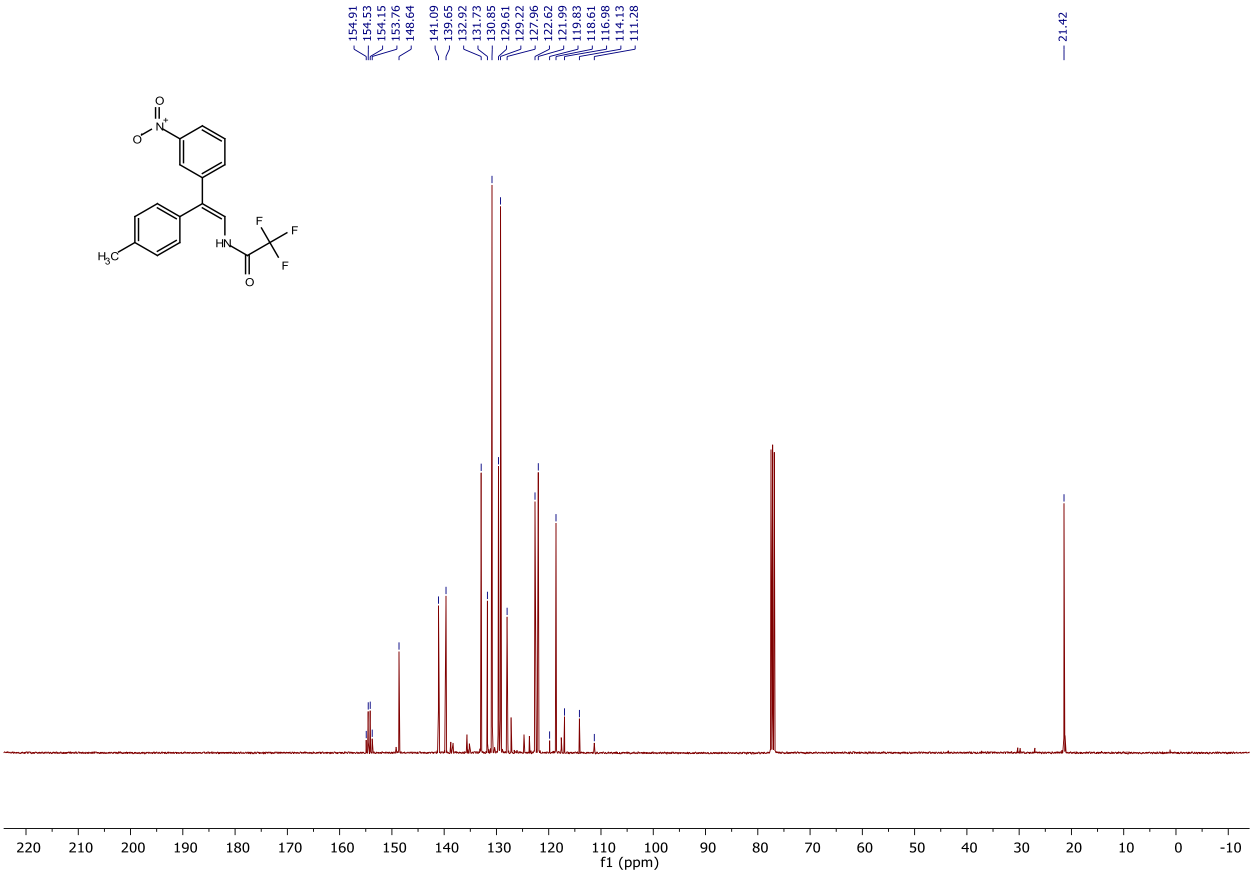

Figure S17. ^{13}C NMR spectrum of **3aa** ($CDCl_3$, 101 MHz)

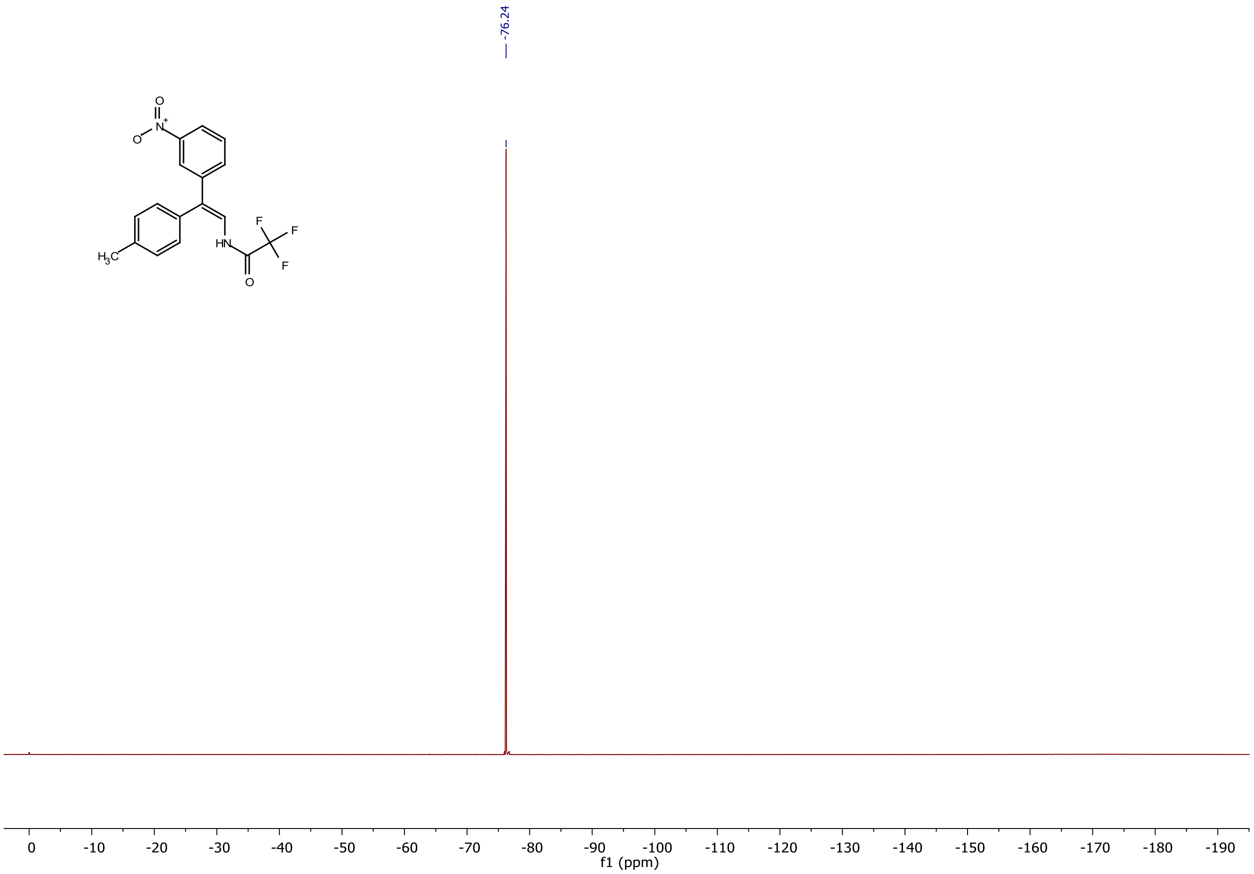

Figure S18. ^{19}F NMR spectrum of **3aa** ($CDCl_3$, 377 MHz)

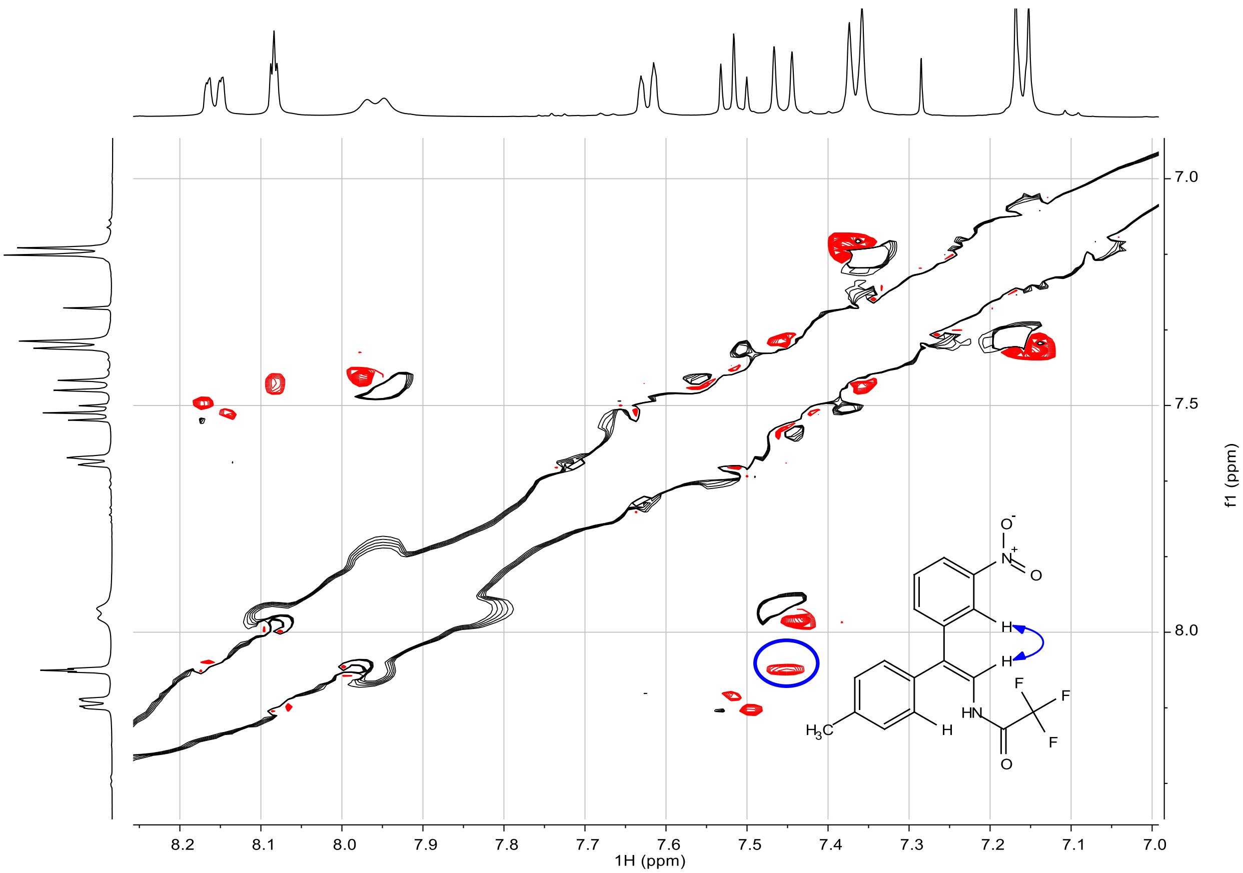

Figure S19. 2D ^{1}H-^{1}H ROESY NMR spectrum of **3aa** ($CDCl_3$, 500 MHz)

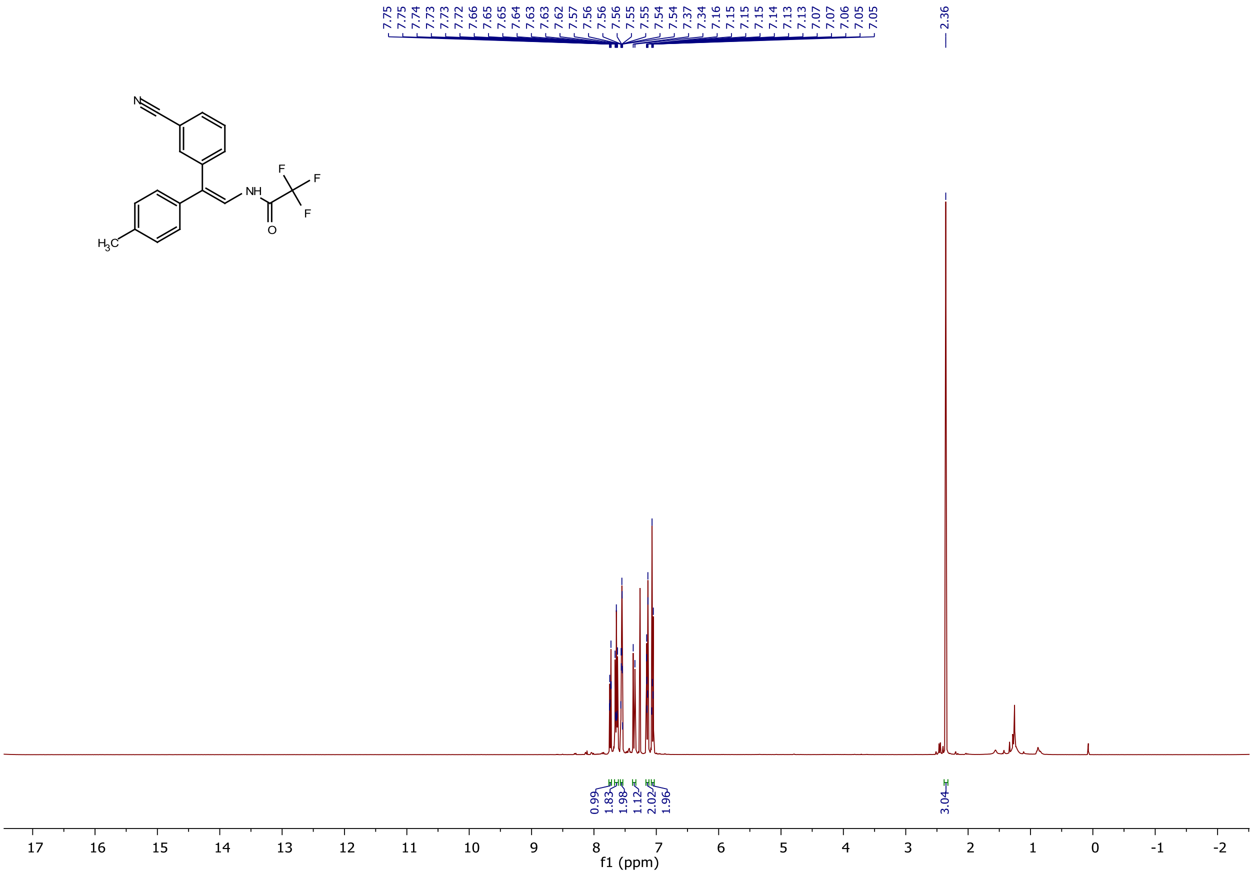

Figure S20. ^{1}H NMR spectrum of **2ab** ($CDCl_3$, 400 MHz)

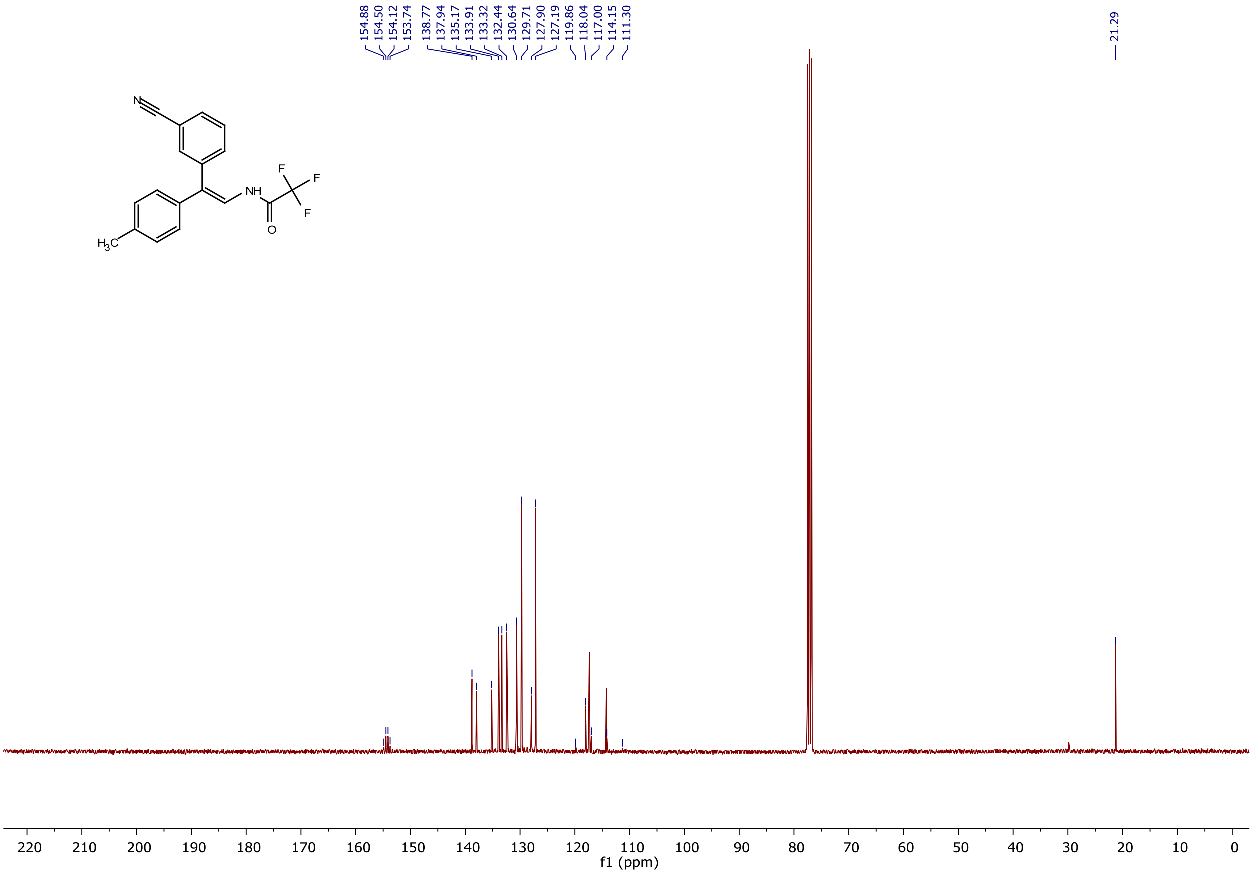

Figure S21. ^{13}C NMR spectrum of **2ab** ($CDCl_3$, 101 MHz)

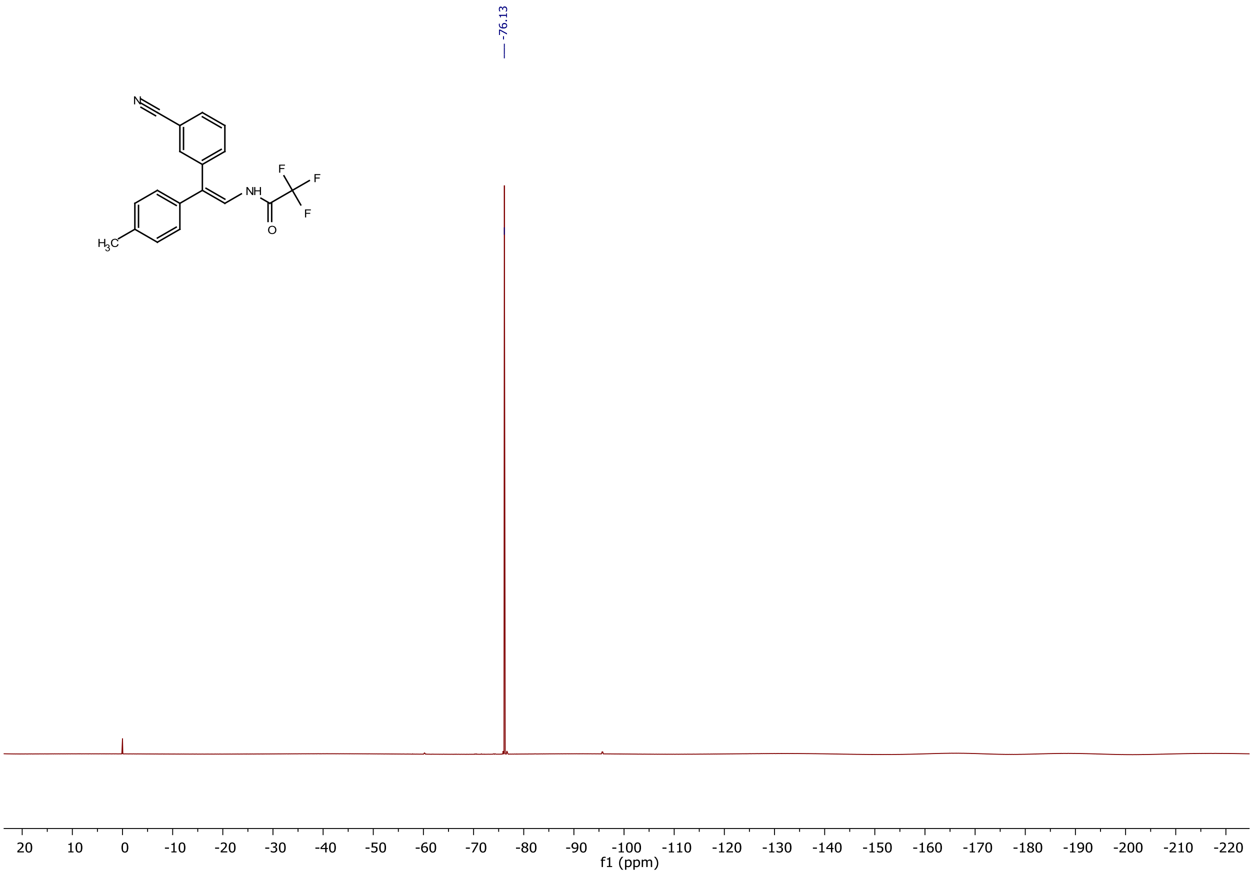

Figure S22. ^{19}F NMR spectrum of **2ab** ($CDCl_3$, 377 MHz)

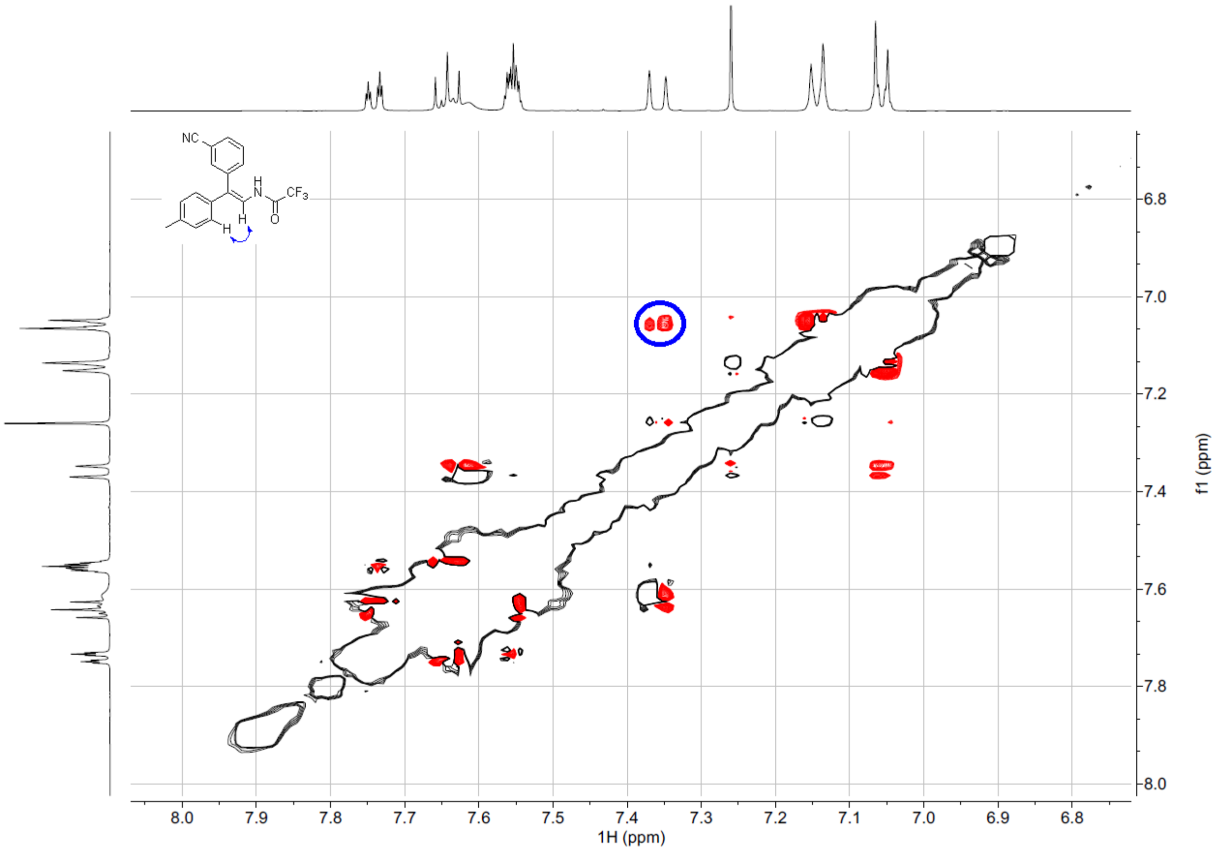

Figure S23. 2D ^{1}H-^{1}H ROESY NMR spectrum of **2ab** ($CDCl_3$, 500 MHz)

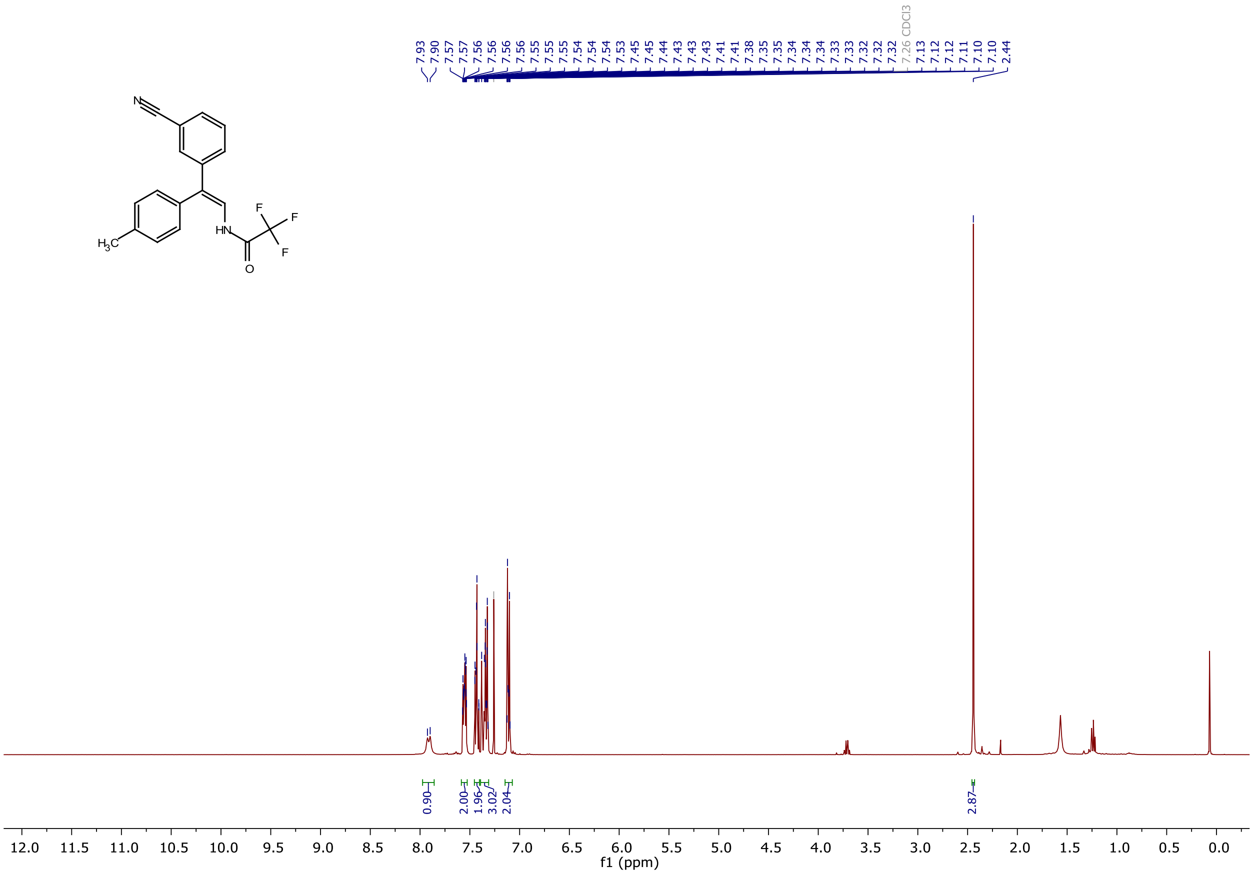

Figure S24. ^{1}H NMR spectrum of **3ab** ($CDCl_3$, 400 MHz)

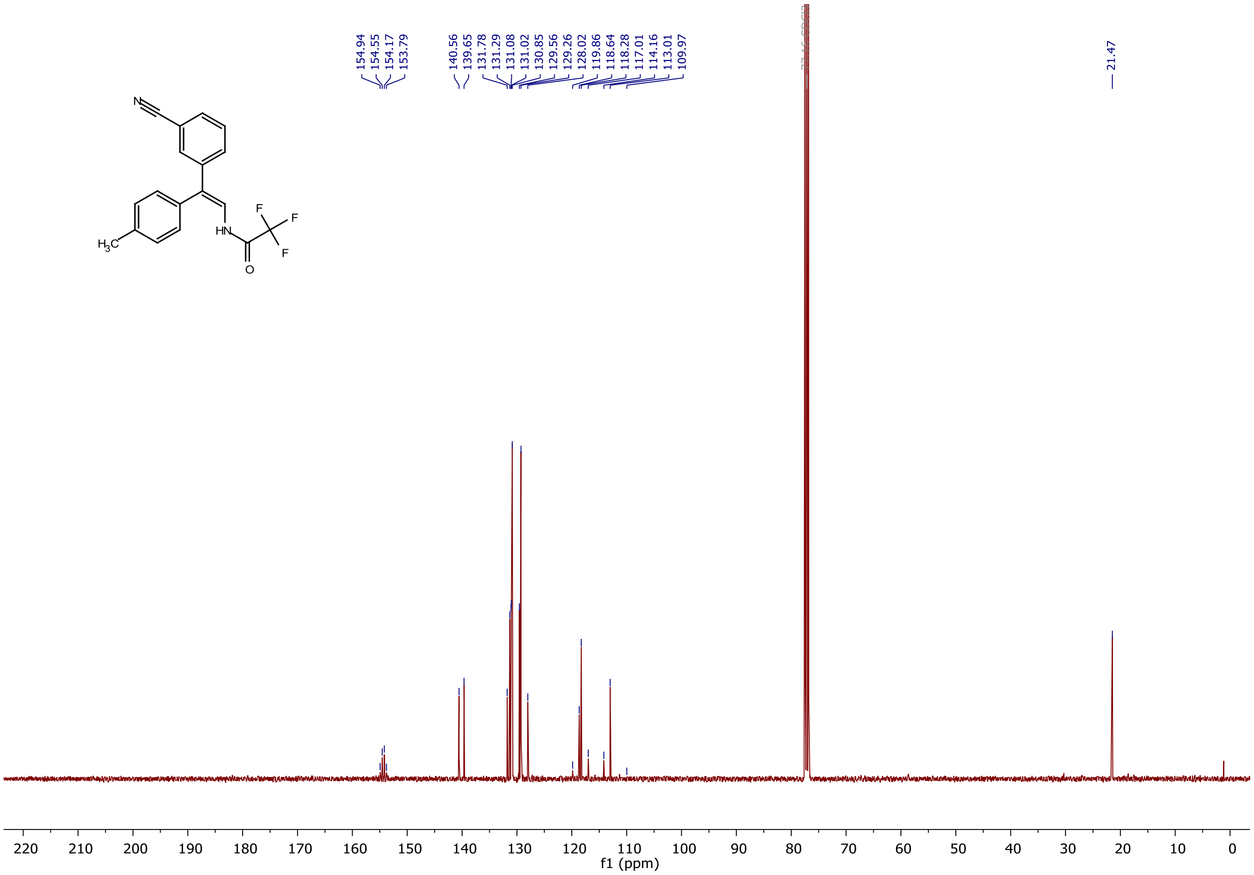

Figure S25. ^{13}C NMR spectrum of **3ab** ($CDCl_3$, 101 MHz)

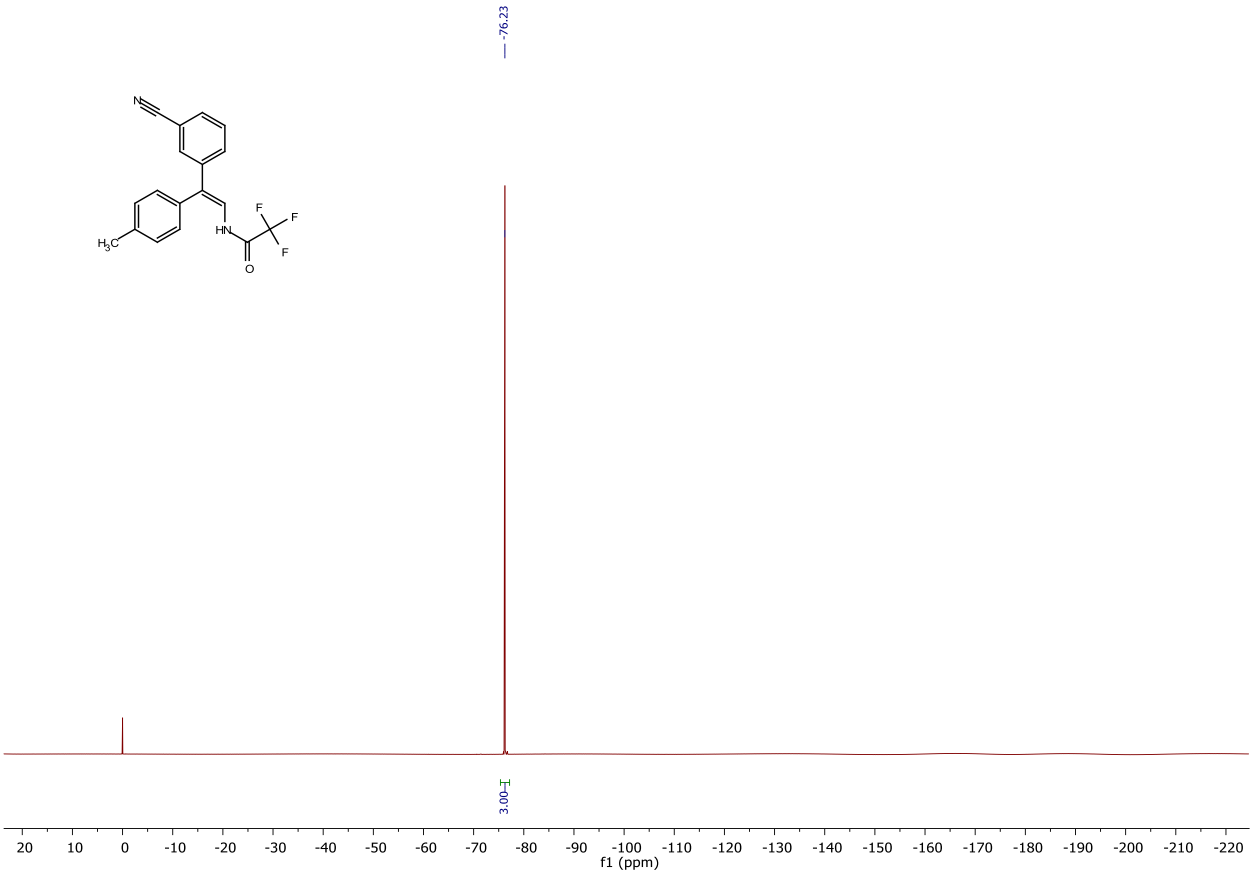

Figure S26. ^{19}F NMR spectrum of **3ab** ($CDCl_3$, 377 MHz)

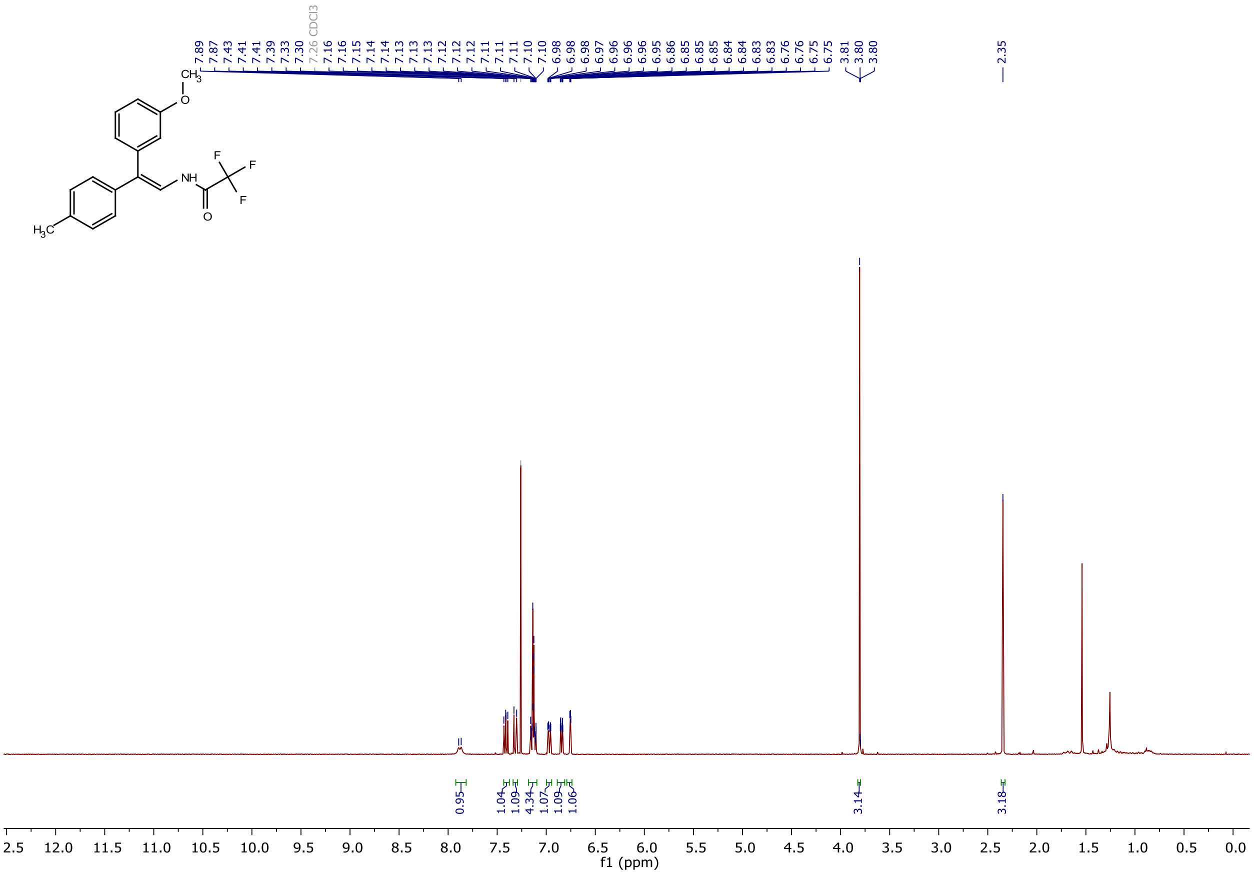

Figure S27. ^{1}H NMR spectrum of **2ac** ($CDCl_3$, 400 MHz)

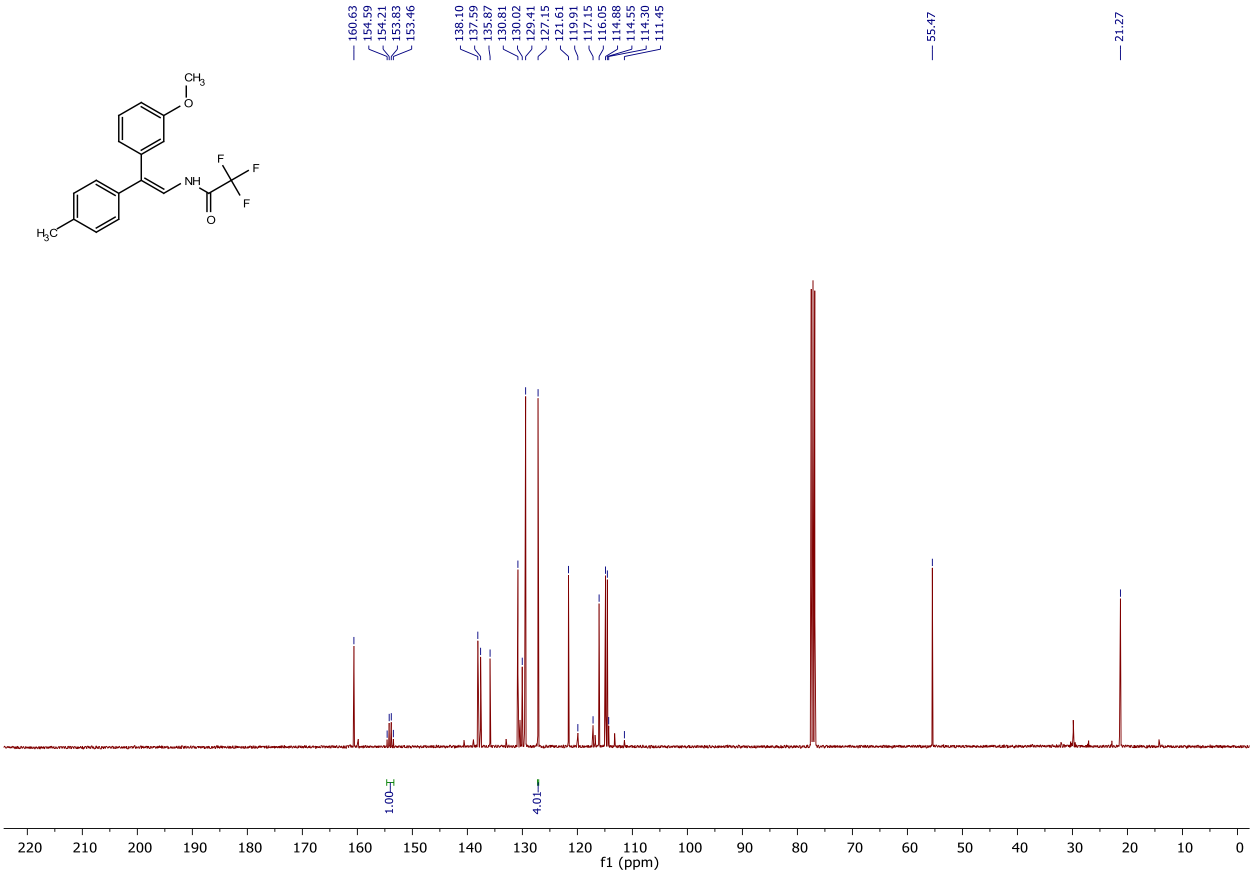

Figure S28. ^{13}C NMR spectrum of **2ac** ($CDCl_3$, 101 MHz)

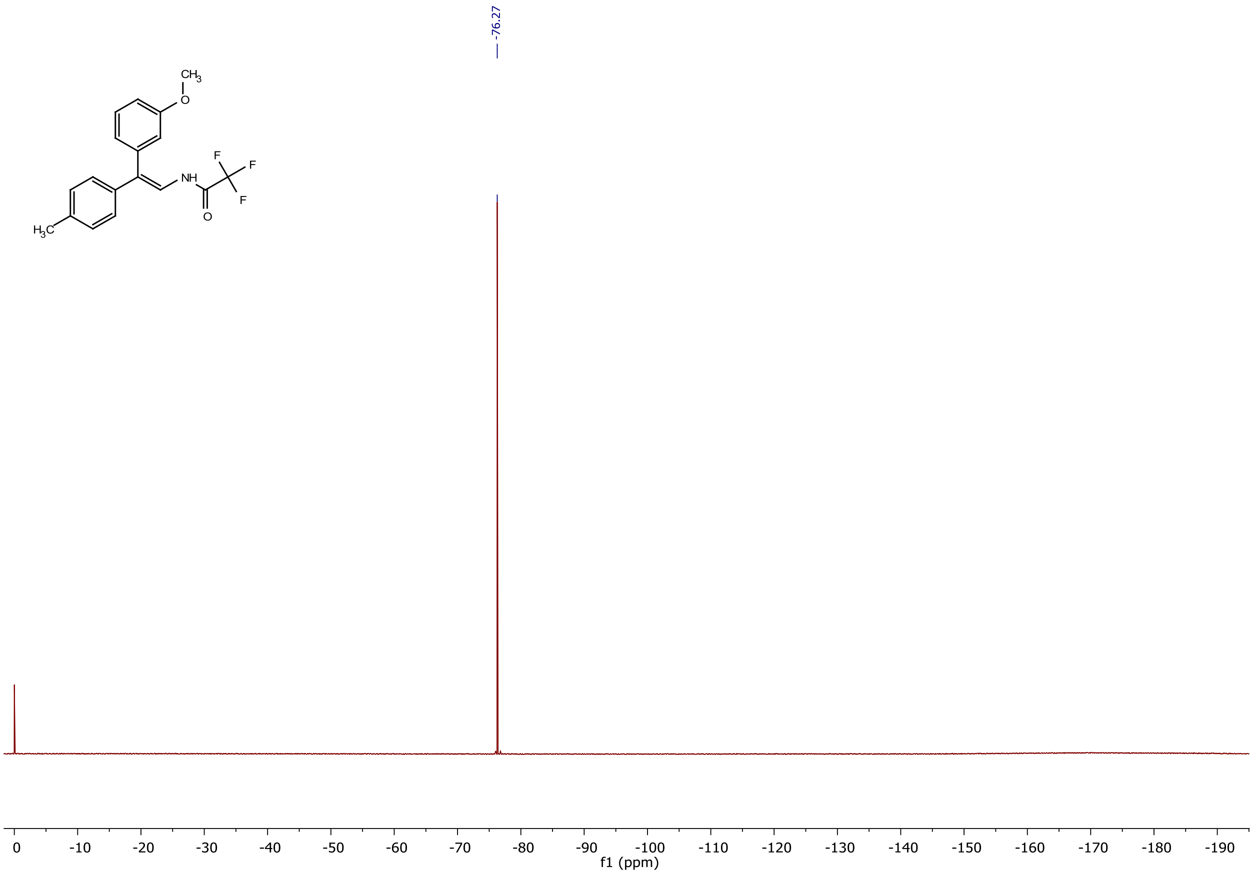

Figure S29. ^{19}F NMR spectrum of **2ac** ($CDCl_3$, 377 MHz)

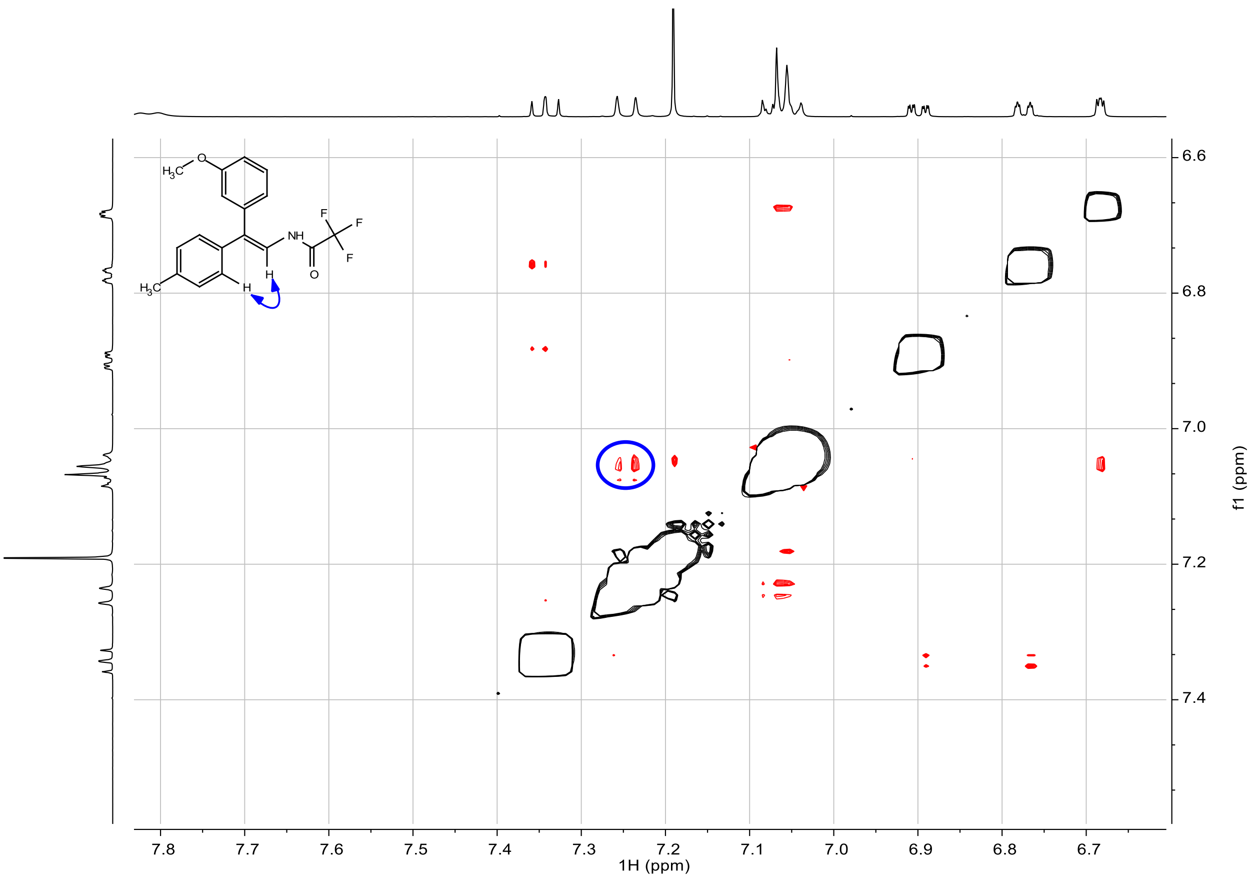

Figure S30. 2D ^{1}H-^{1}H ROESY NMR spectrum of **2ac** ($CDCl_3$, 500 MHz)

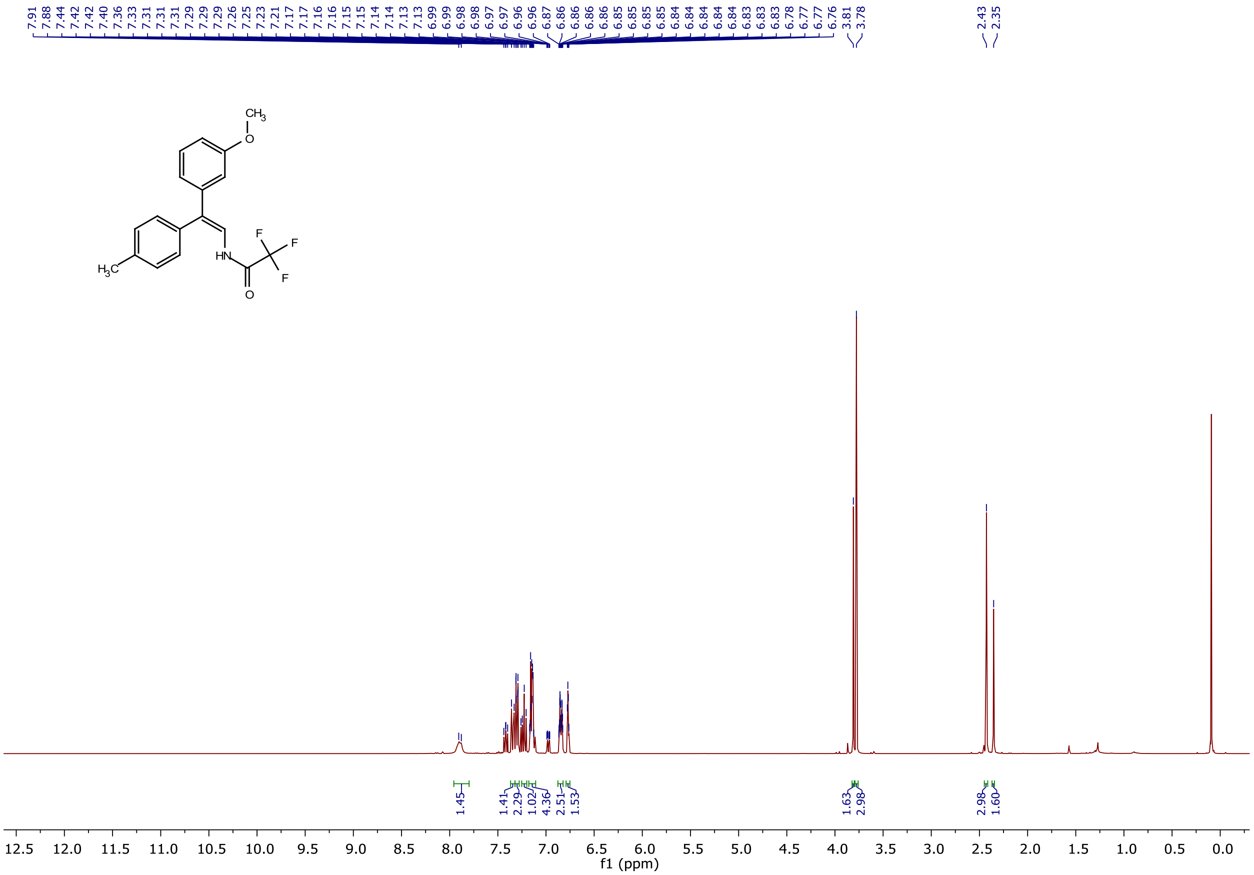

Figure S31. ^{1}H NMR spectrum of **3ac** ($CDCl_3$, 400 MHz)

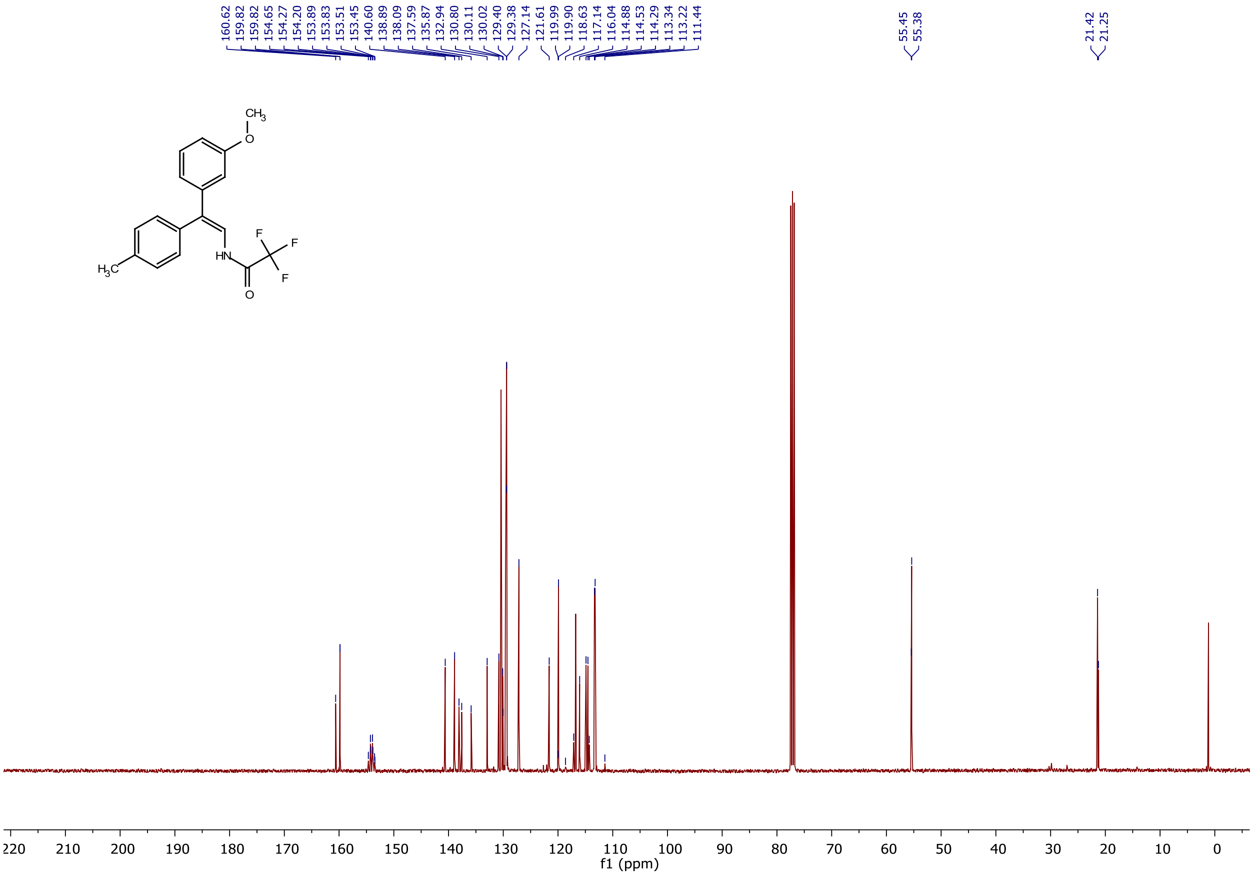

Figure S32. ^{13}C NMR spectrum of **3ac** ($CDCl_3$, 101 MHz)

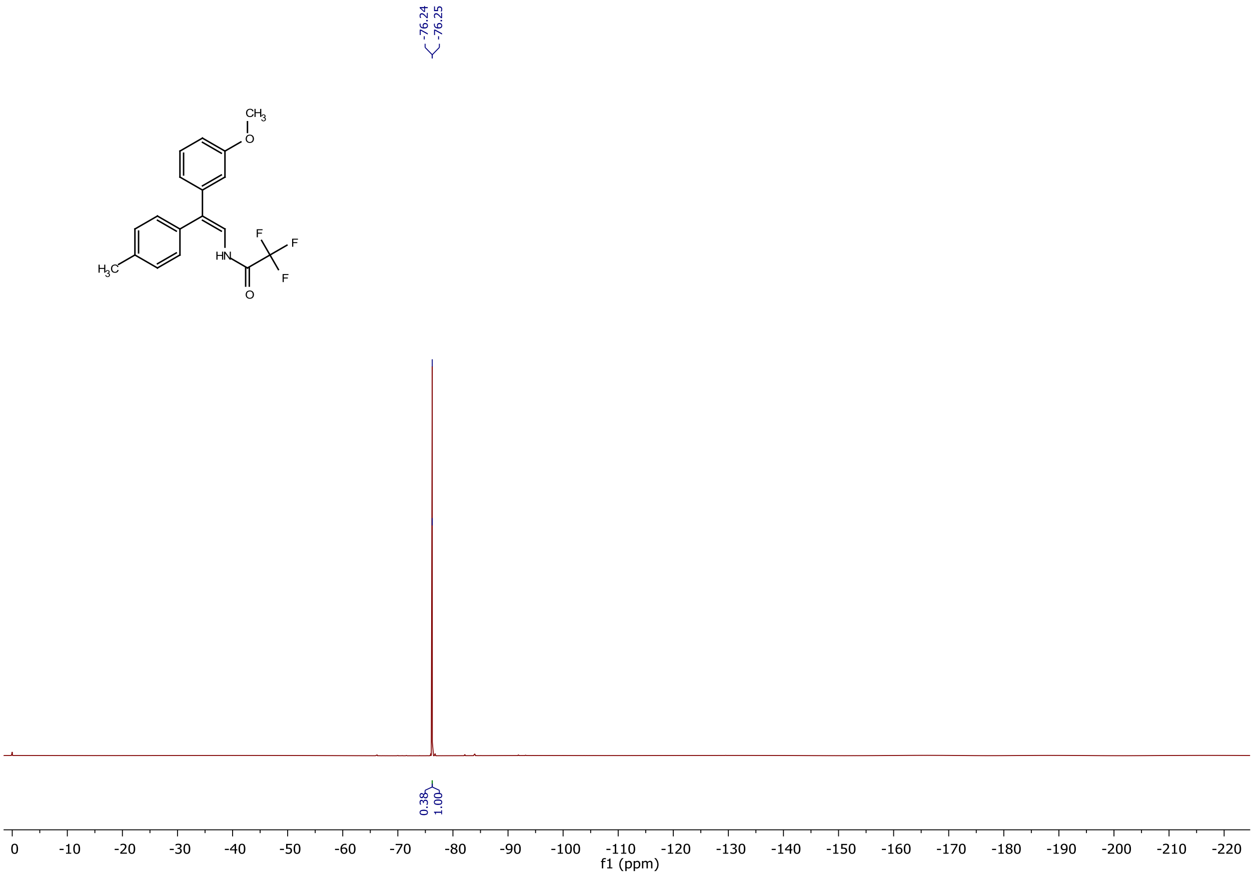

Figure S33. ^{19}F NMR spectrum of **3ac** ($CDCl_3$, 377 MHz)

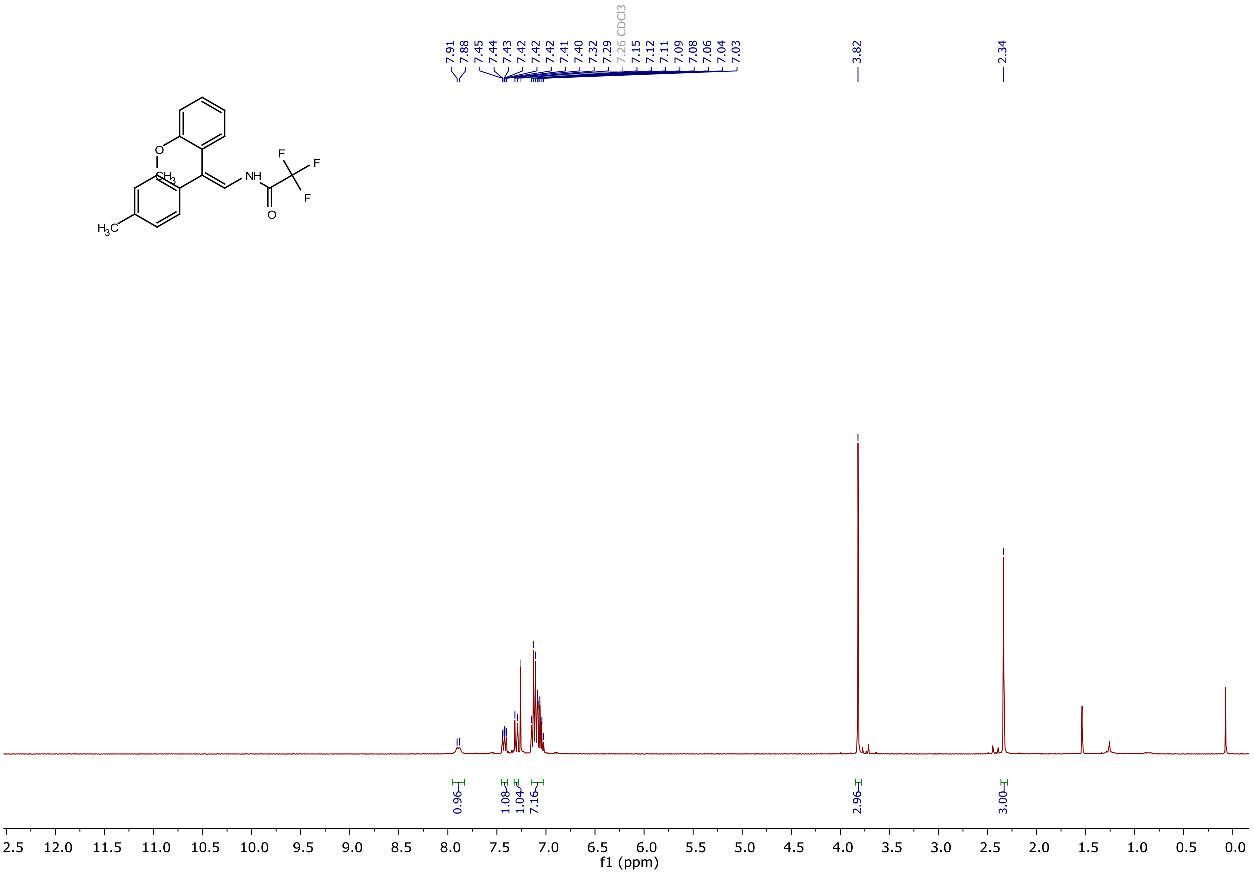

Figure S34. ^{1}H NMR spectrum of **2ad** ($CDCl_3$, 400 MHz)

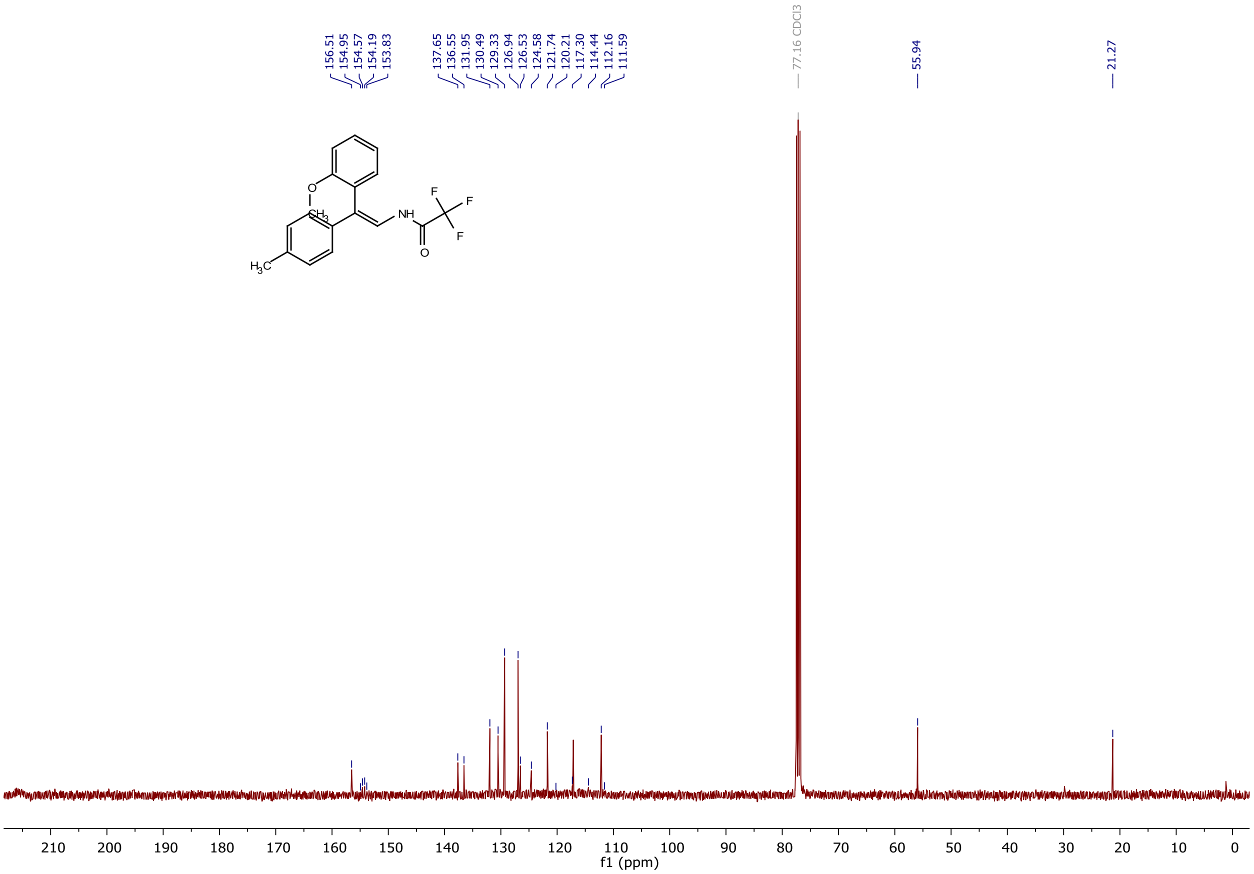

Figure S35. ^{13}C NMR spectrum of **2ad** ($CDCl_3$, 101 MHz)

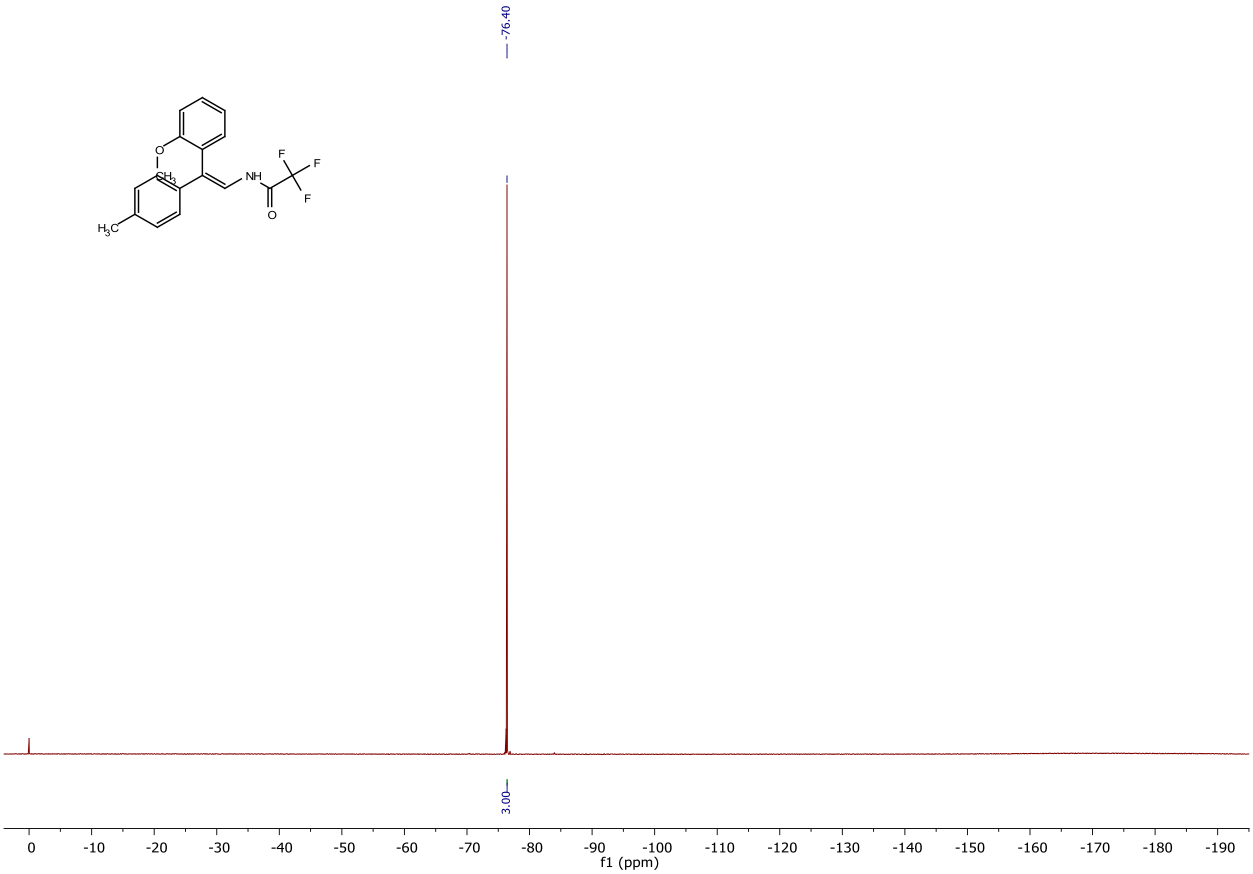

Figure S36. ^{19}F NMR spectrum of **2ad** ($CDCl_3$, 377 MHz)

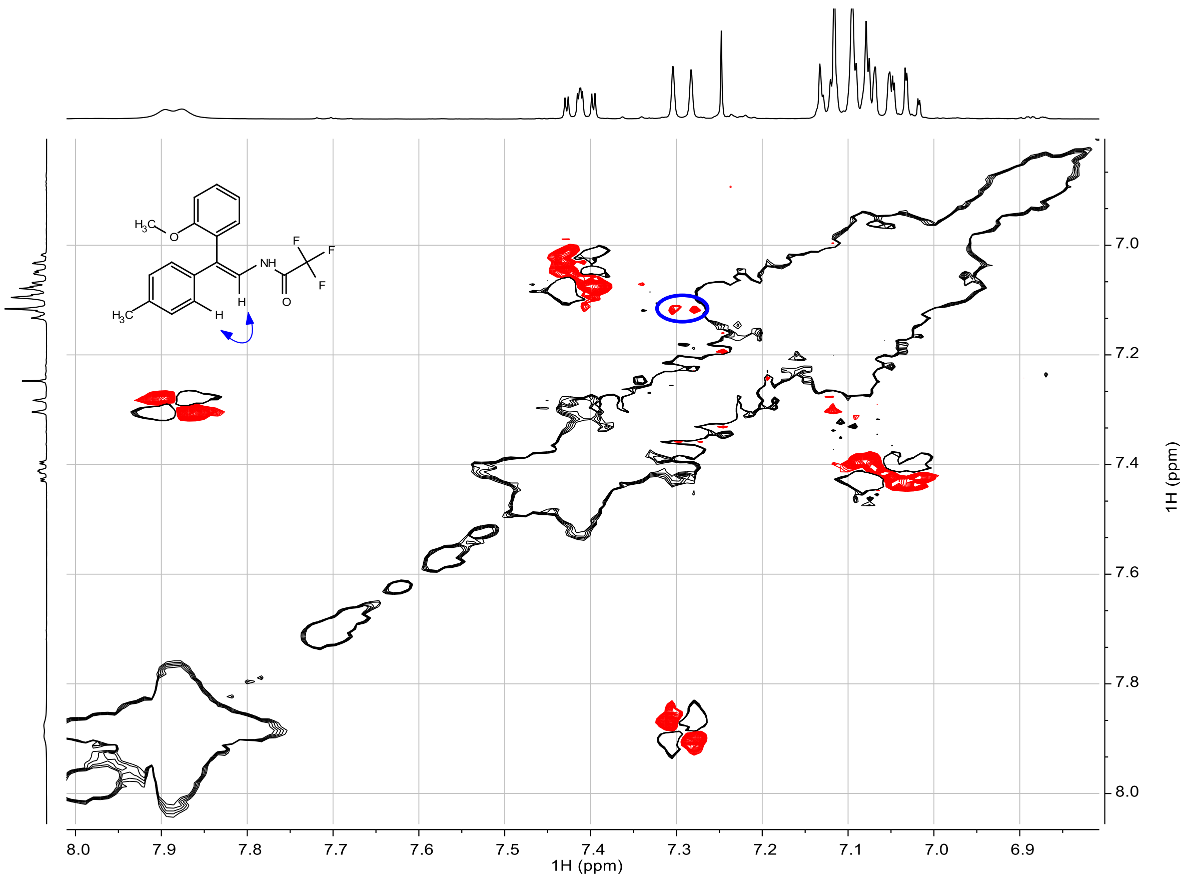

Figure S37. 2D ^{1}H-^{1}H ROESY NMR spectrum of **2ad** ($CDCl_3$, 500 MHz)

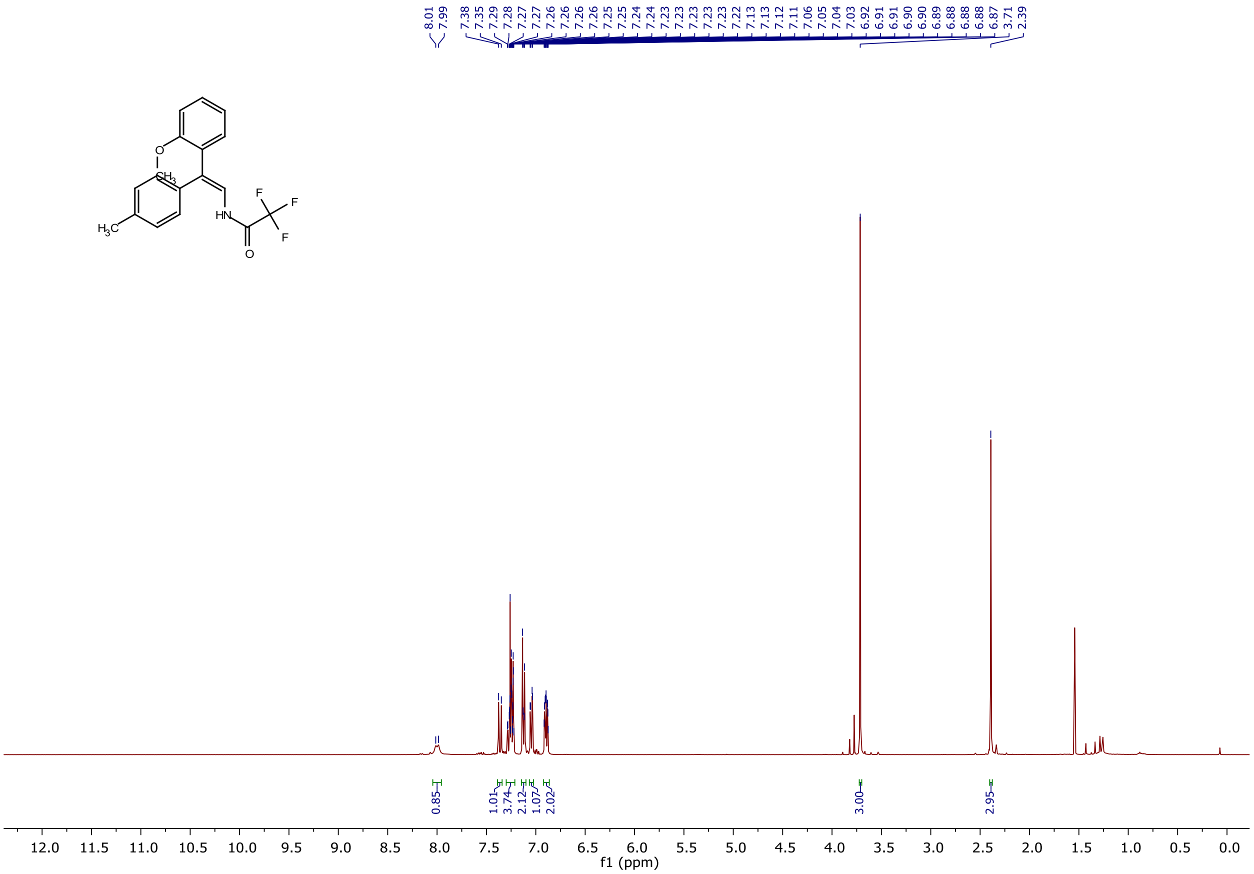

Figure S38. ^{1}H NMR spectrum of **3ad** ($CDCl_3$, 400 MHz)

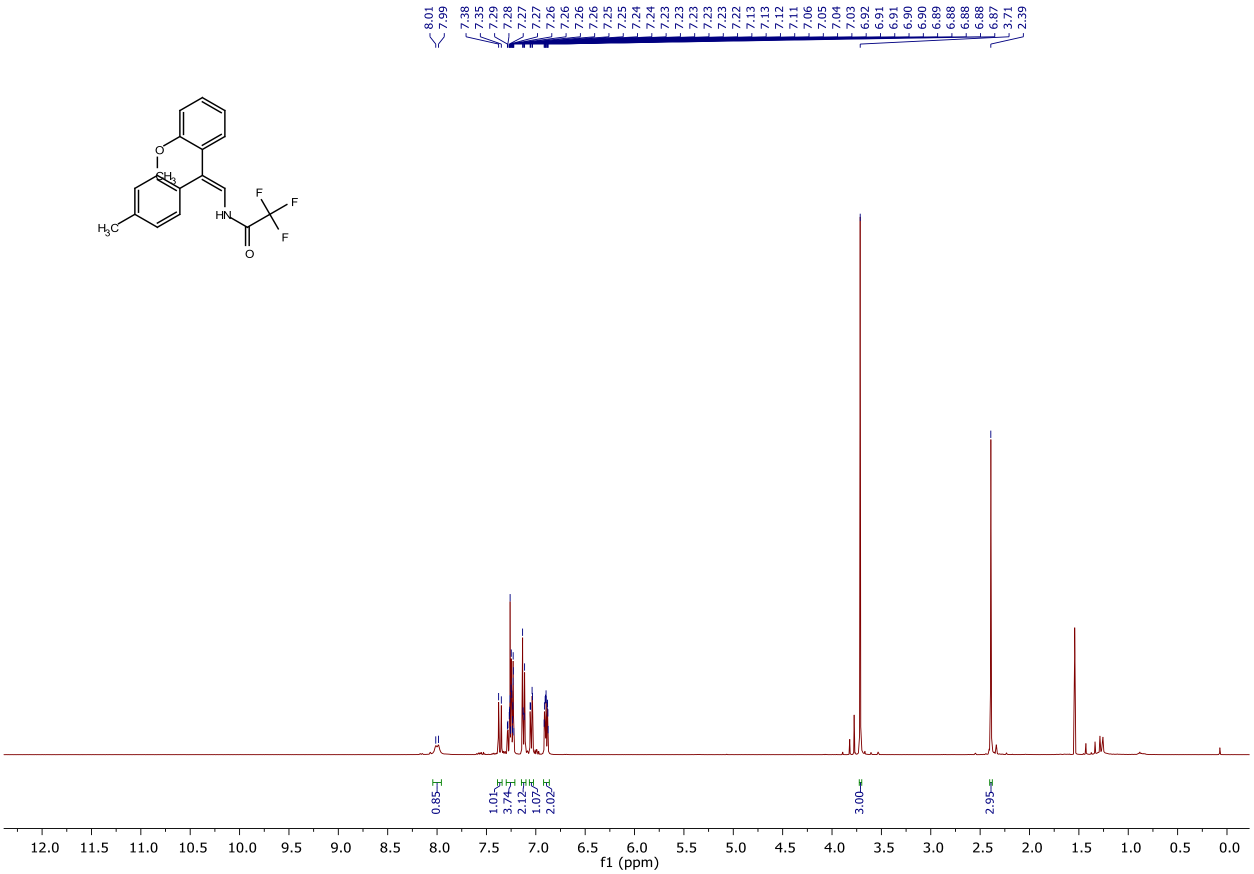

Figure S39. ^{13}C NMR spectrum of **3ad** ($CDCl_3$, 101 MHz)

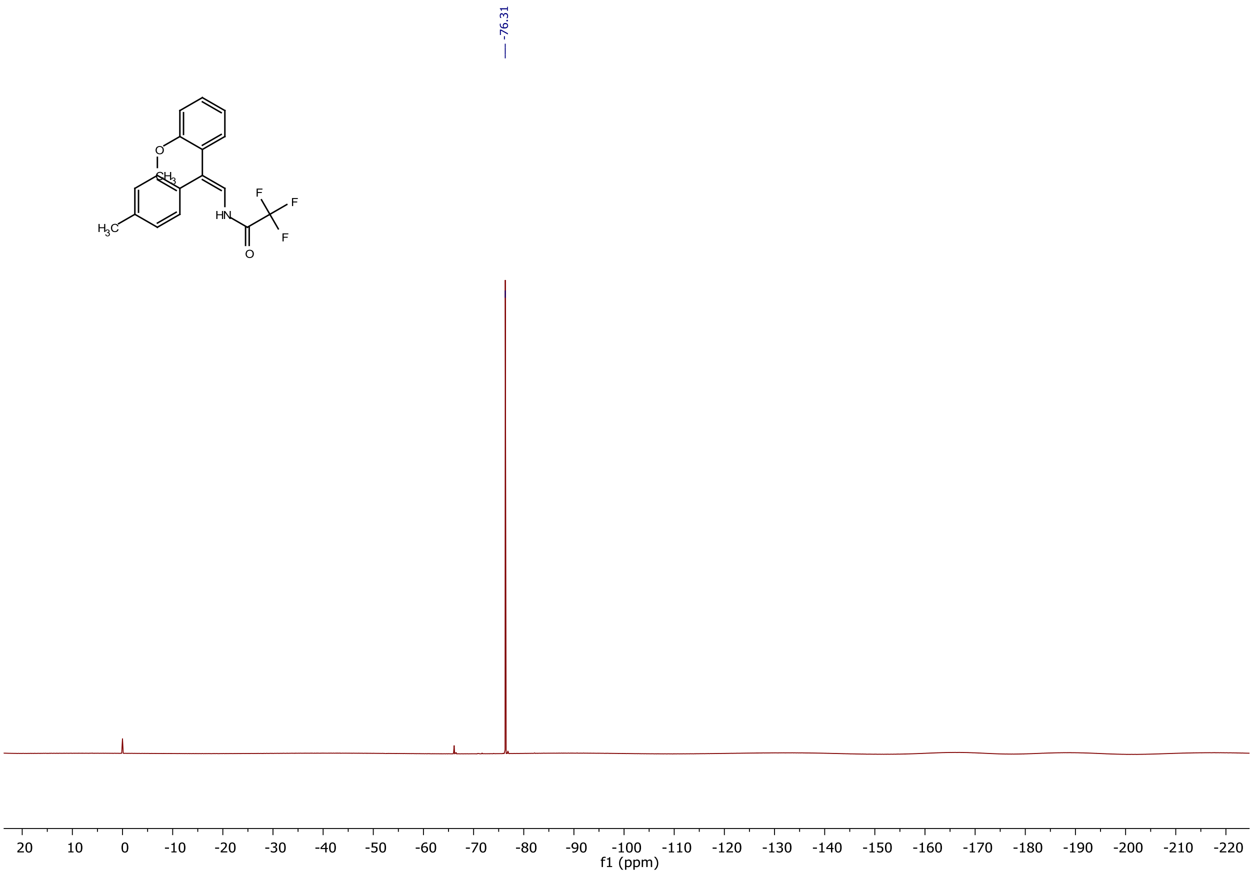

Figure S40. ^{19}F NMR spectrum of **3ad** ($CDCl_3$, 377 MHz)

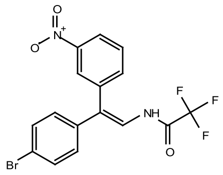

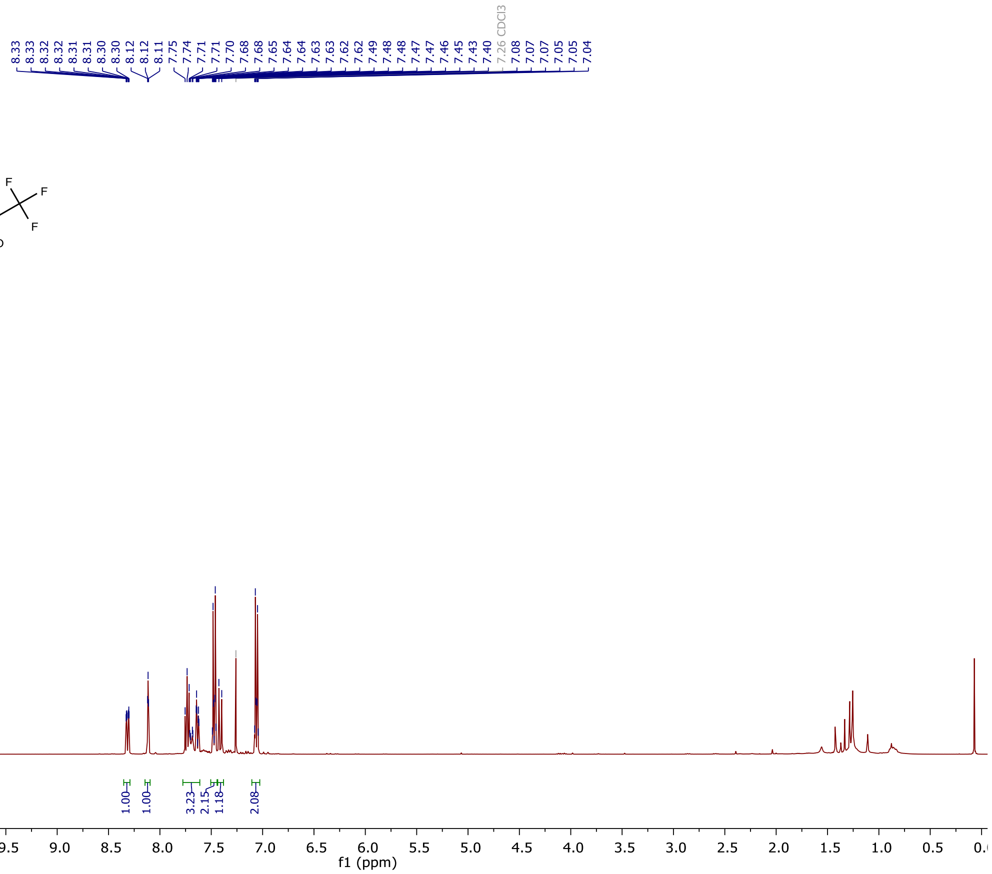

Figure S41. ^{1}H NMR spectrum of **2ba** ($CDCl_3$, 400 MHz)

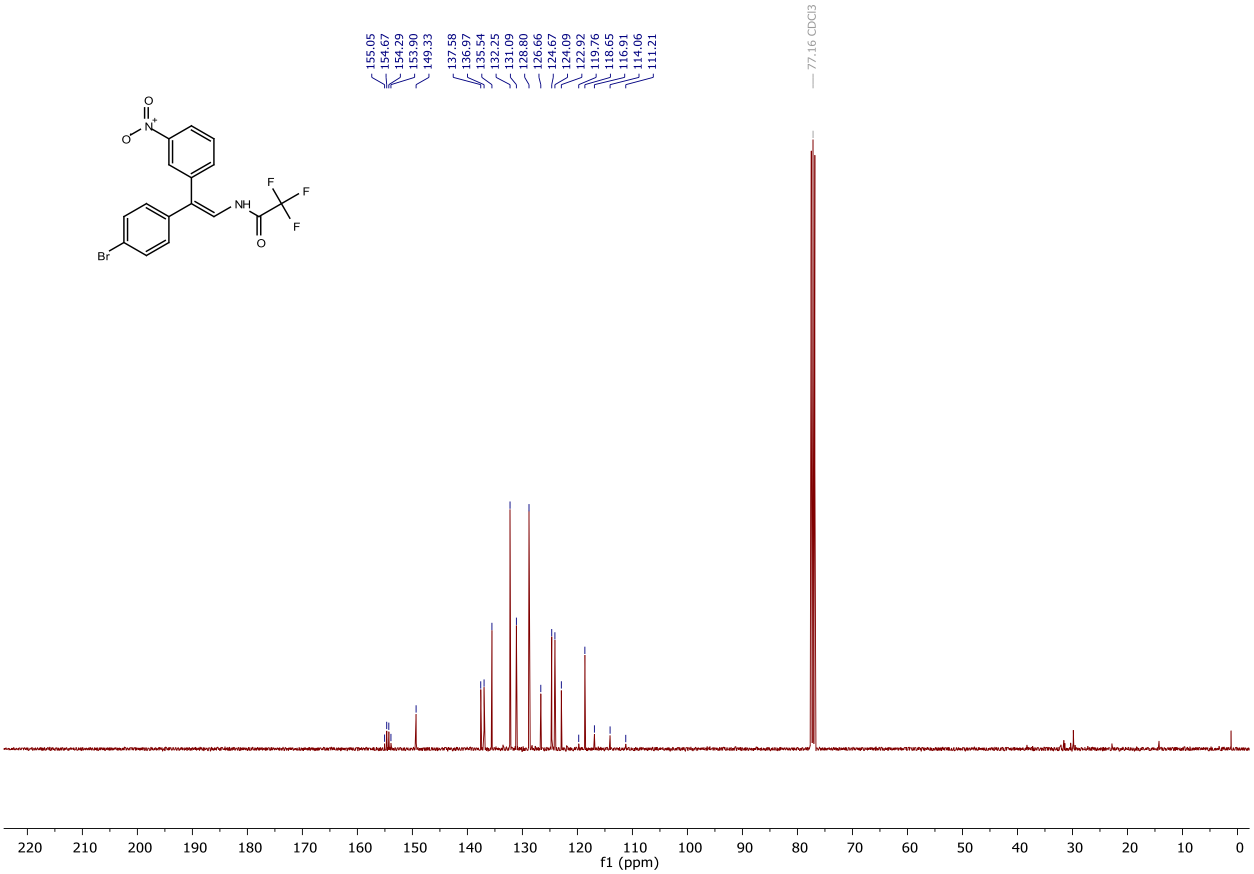

Figure S42. ^{13}C NMR spectrum of **2ba** ($CDCl_3$, 101 MHz)

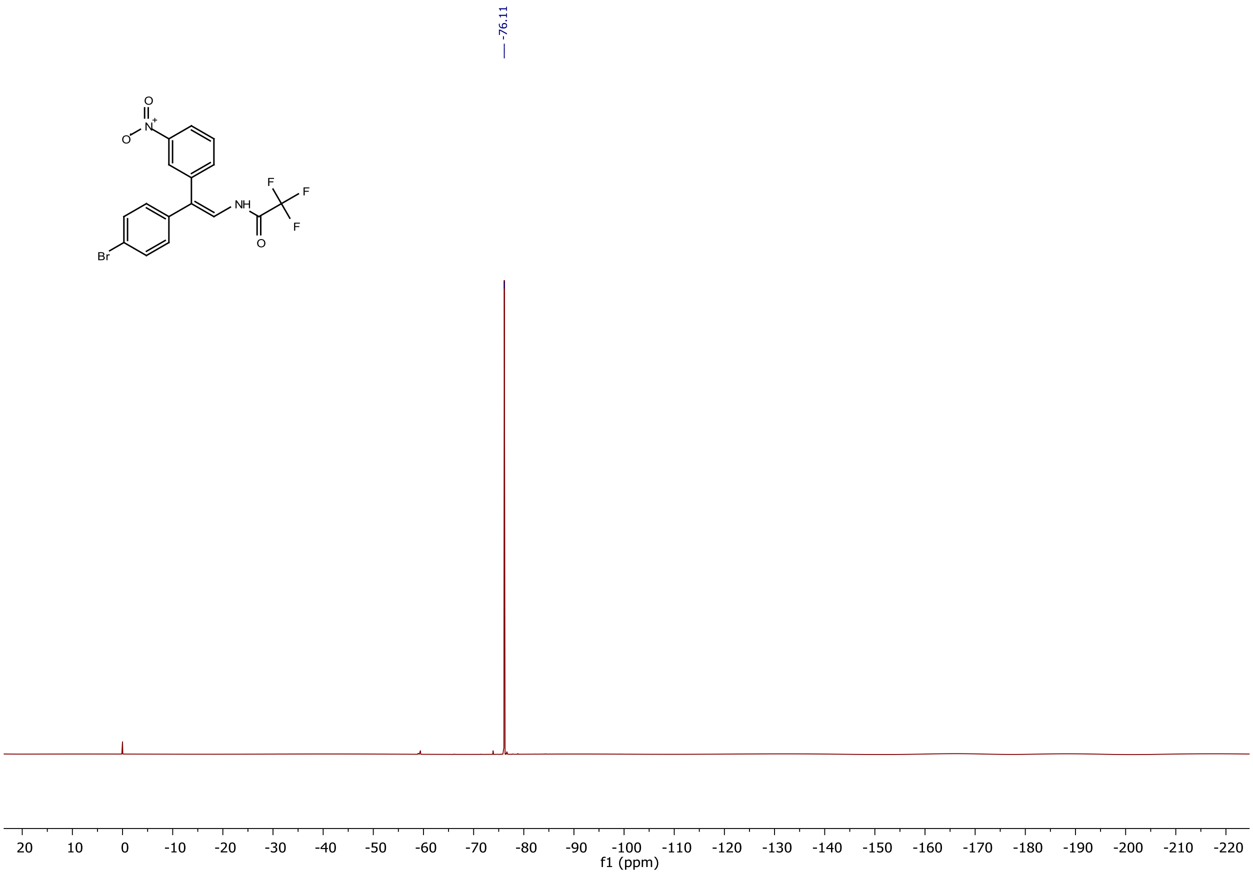

Figure S43. ^{19}F NMR spectrum of **2ba** ($CDCl_3$, 377 MHz)

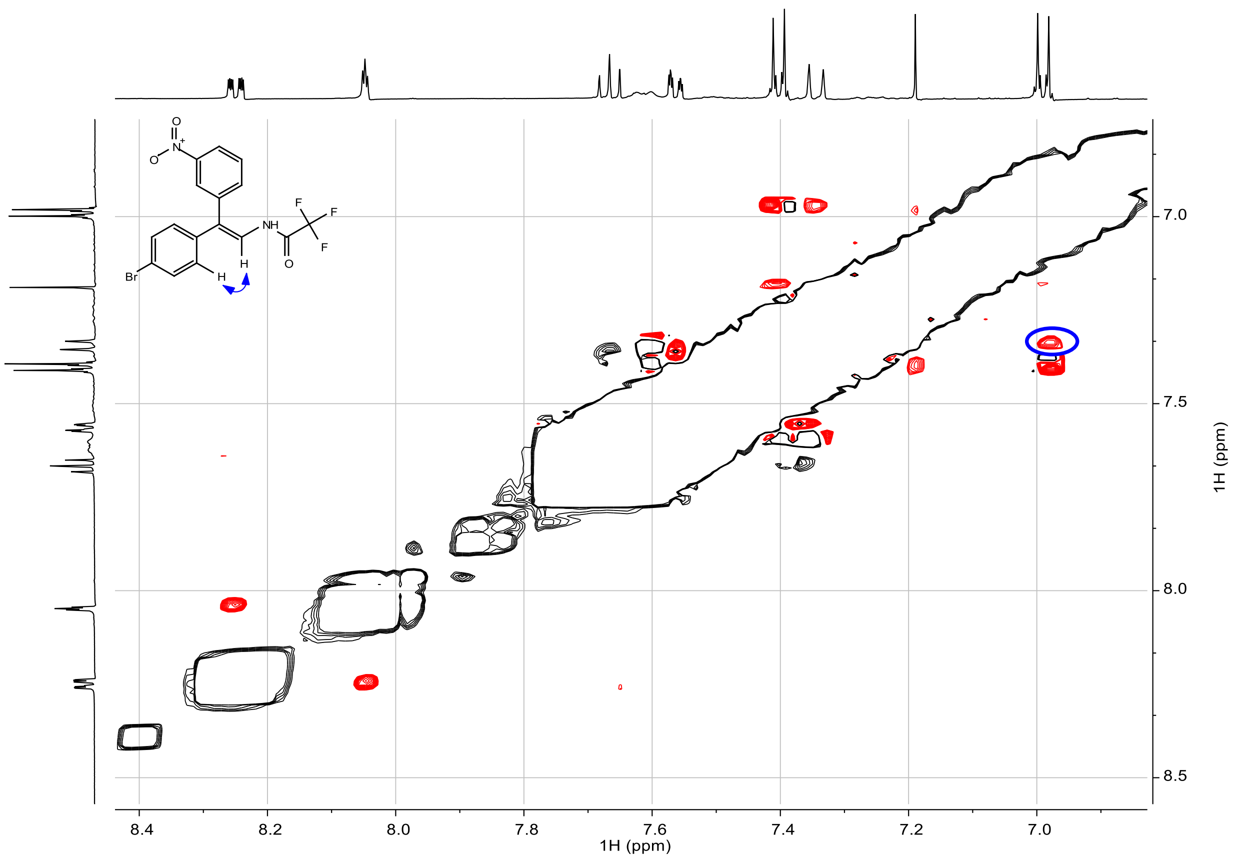

Figure S44. 2D ^1H-^1H ROESY NMR spectrum of **2ba** ($CDCl_3$, 500 MHz)

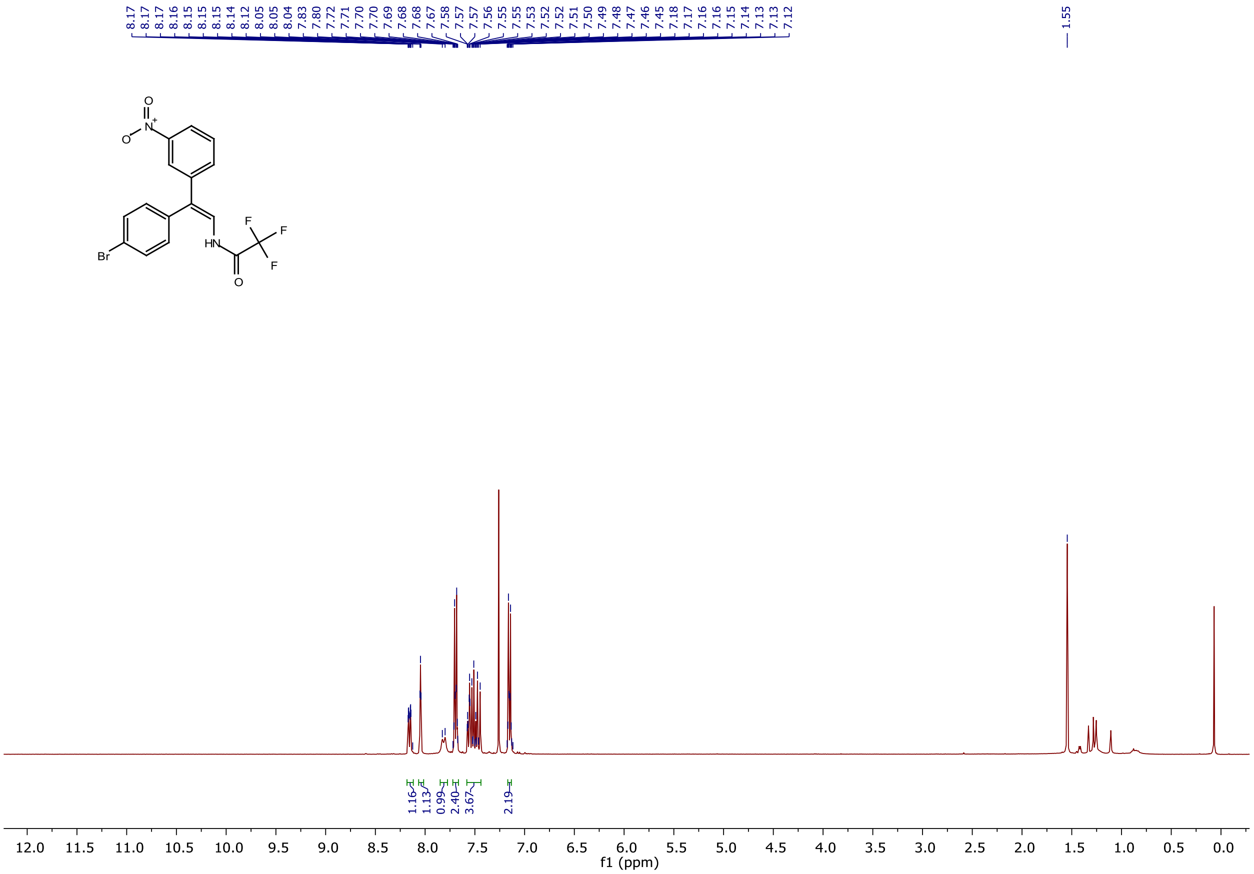

Figure S45. ^{1}H NMR spectrum of **3ba** ($CDCl_3$, 400 MHz)

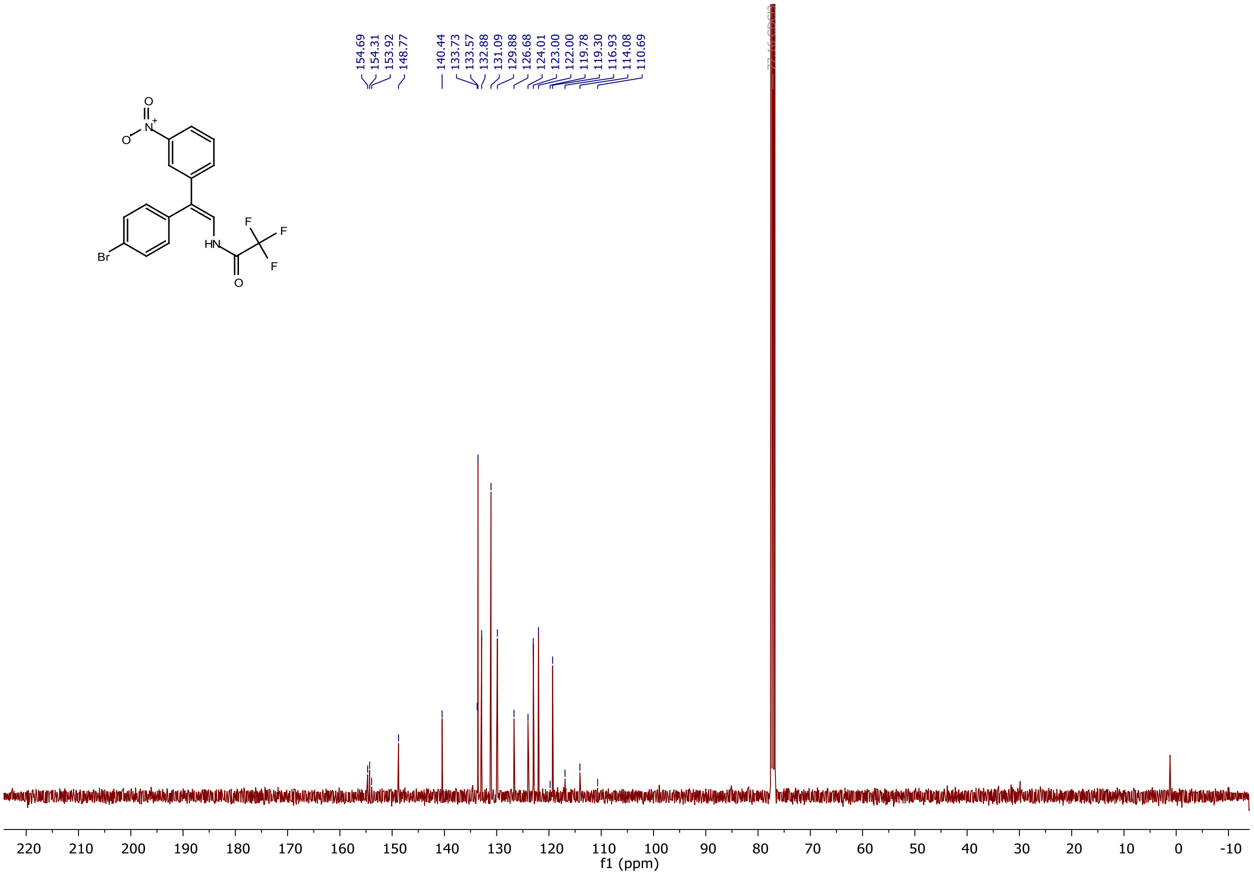

Figure S46. ^{13}C NMR spectrum of **3ba** ($CDCl_3$, 101 MHz)

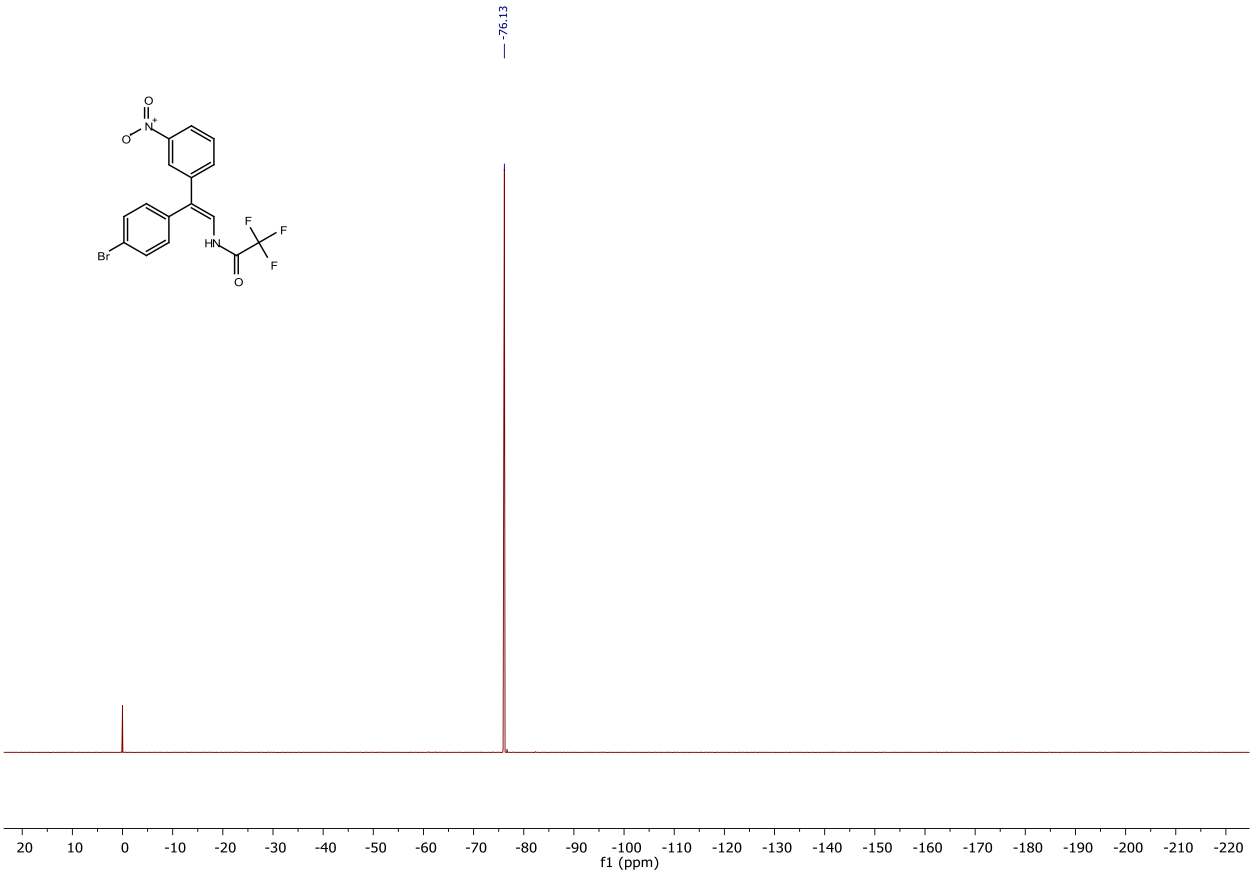

Figure S47. ^{19}F NMR spectrum of **3ba** ($CDCl_3$, 377 MHz)

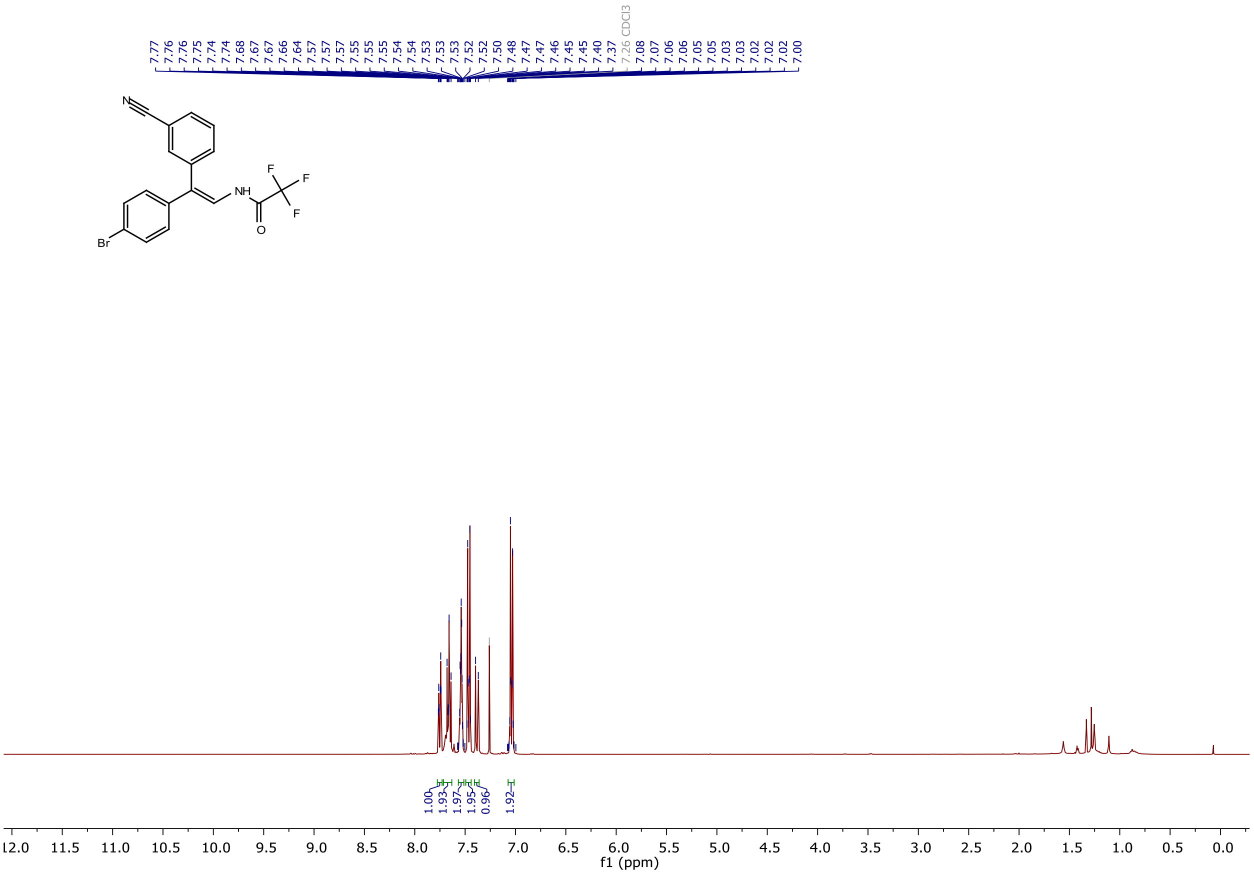

Figure S48. ^{1}H NMR spectrum of **2bb** ($CDCl_3$, 400 MHz)

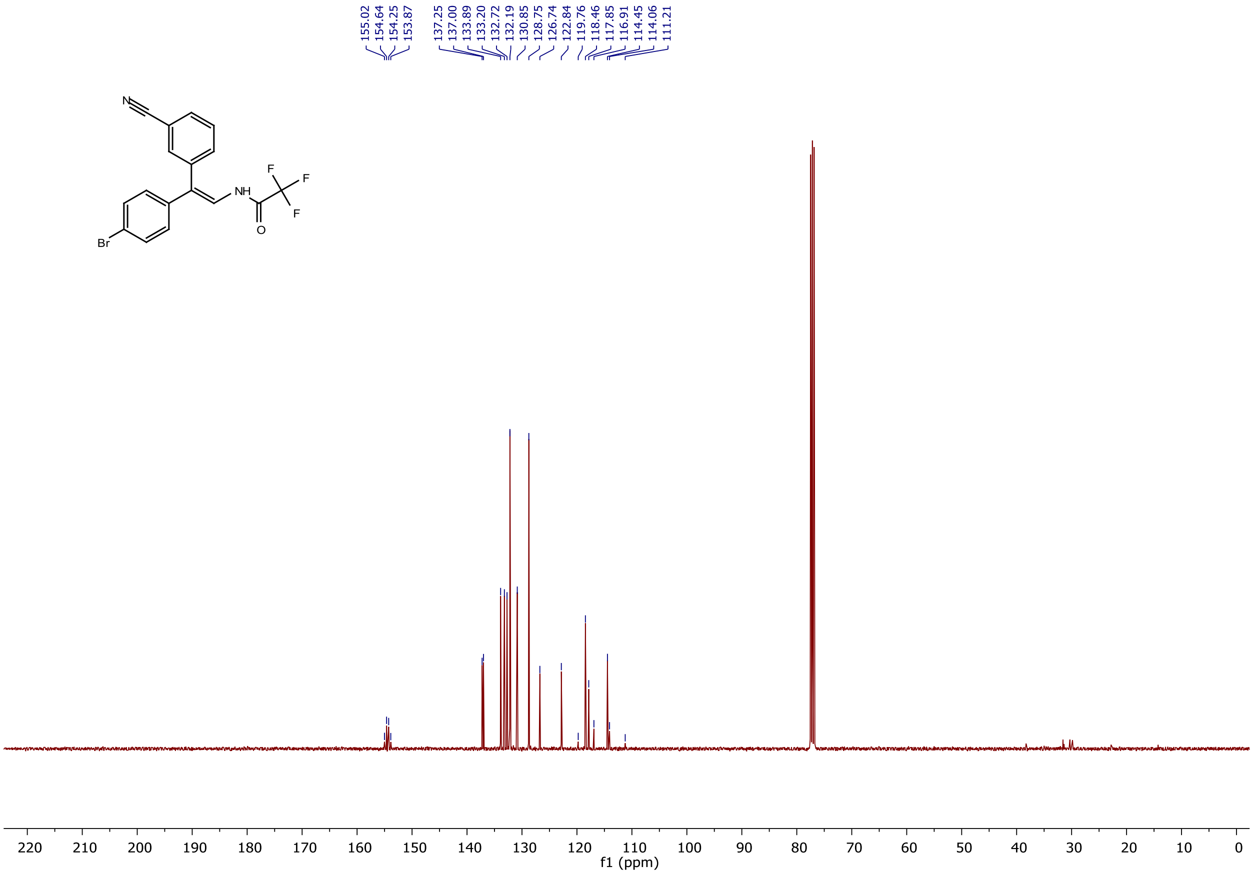

Figure S49. ^{13}C NMR spectrum of **2bb** ($CDCl_3$, 101 MHz)

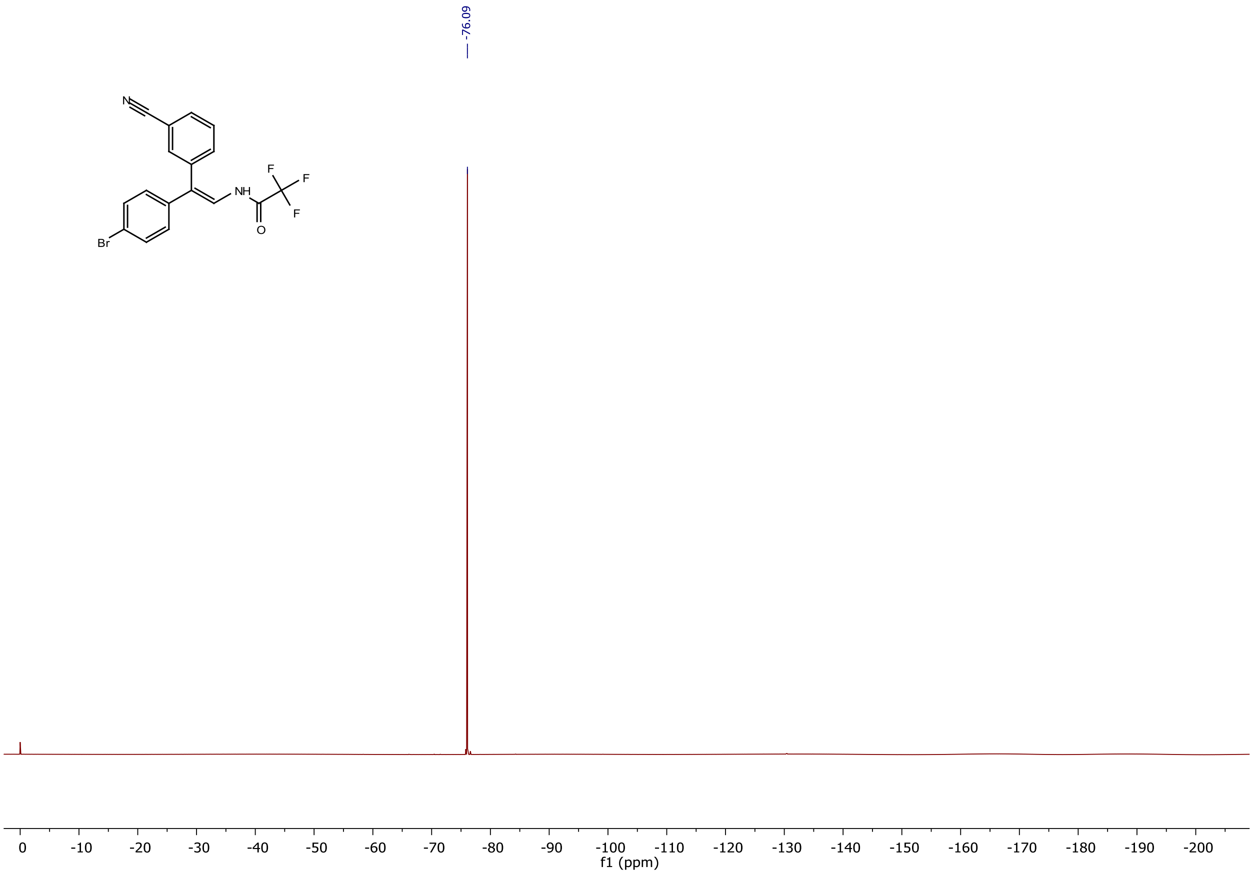

Figure S50. ^{19}F NMR spectrum of **2bb** ($CDCl_3$, 377 MHz)

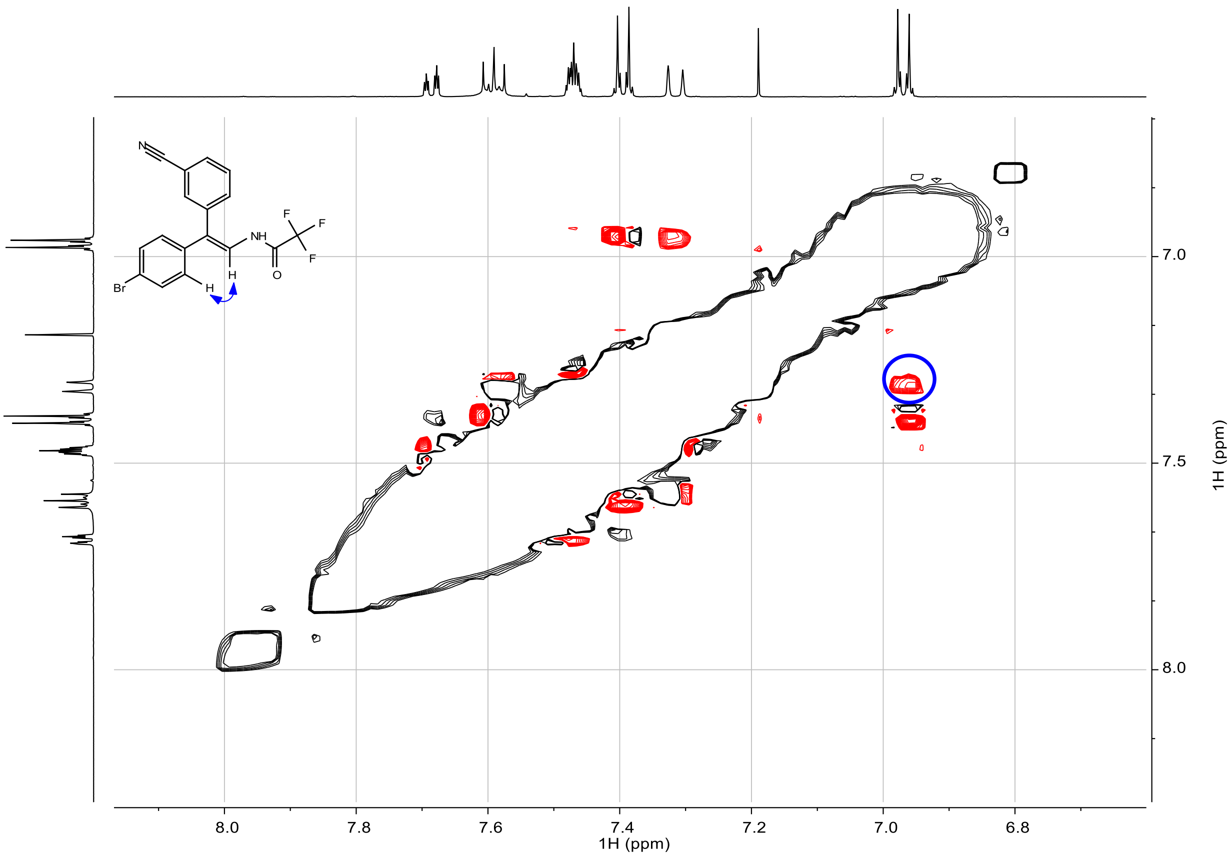

Figure S51. 2D ^{1}H-^{1}H ROESY NMR spectrum of **2bb** ($CDCl_3$, 500 MHz)

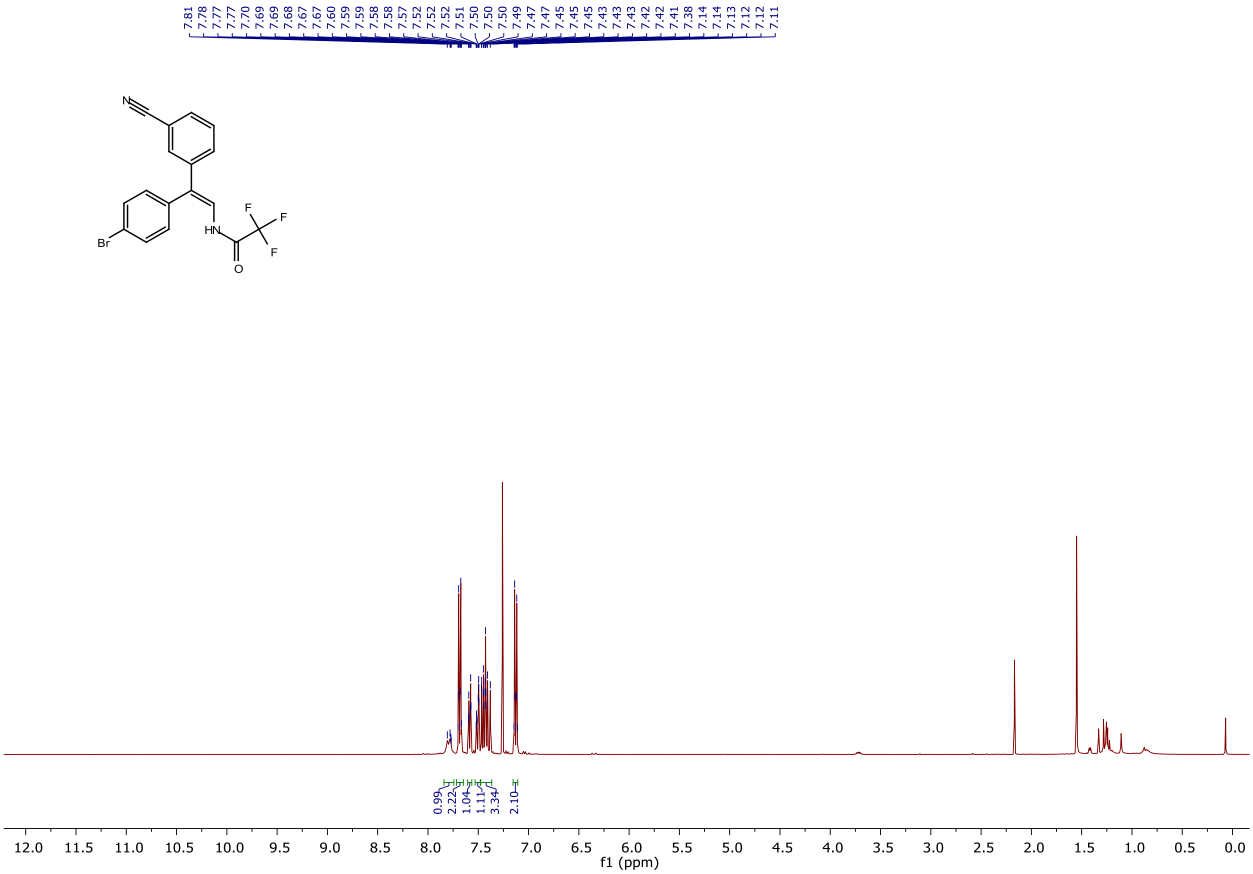

Figure S52. ^{1}H NMR spectrum of **3bb** ($CDCl_3$, 400 MHz)

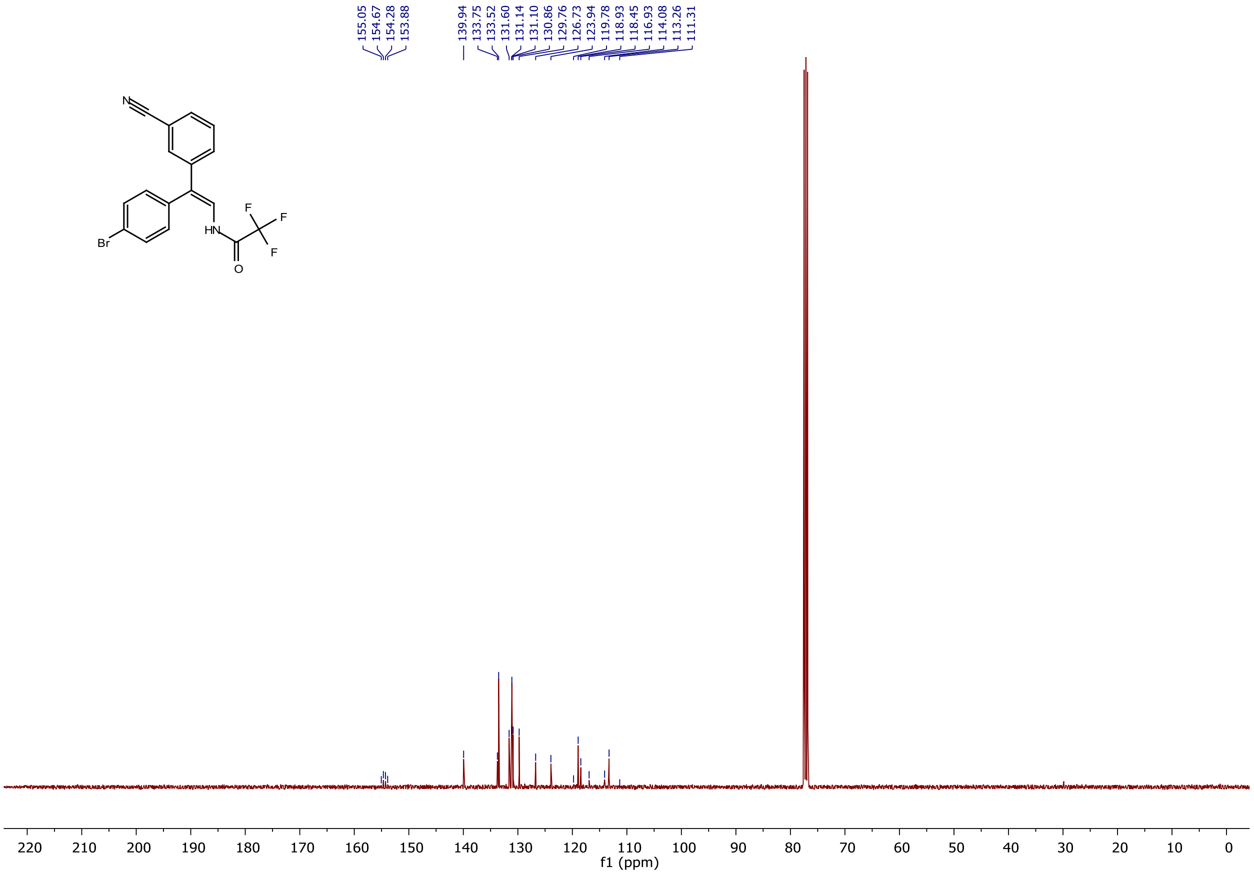

Figure S53. ^{13}C NMR spectrum of **3bb** ($CDCl_3$, 101 MHz)

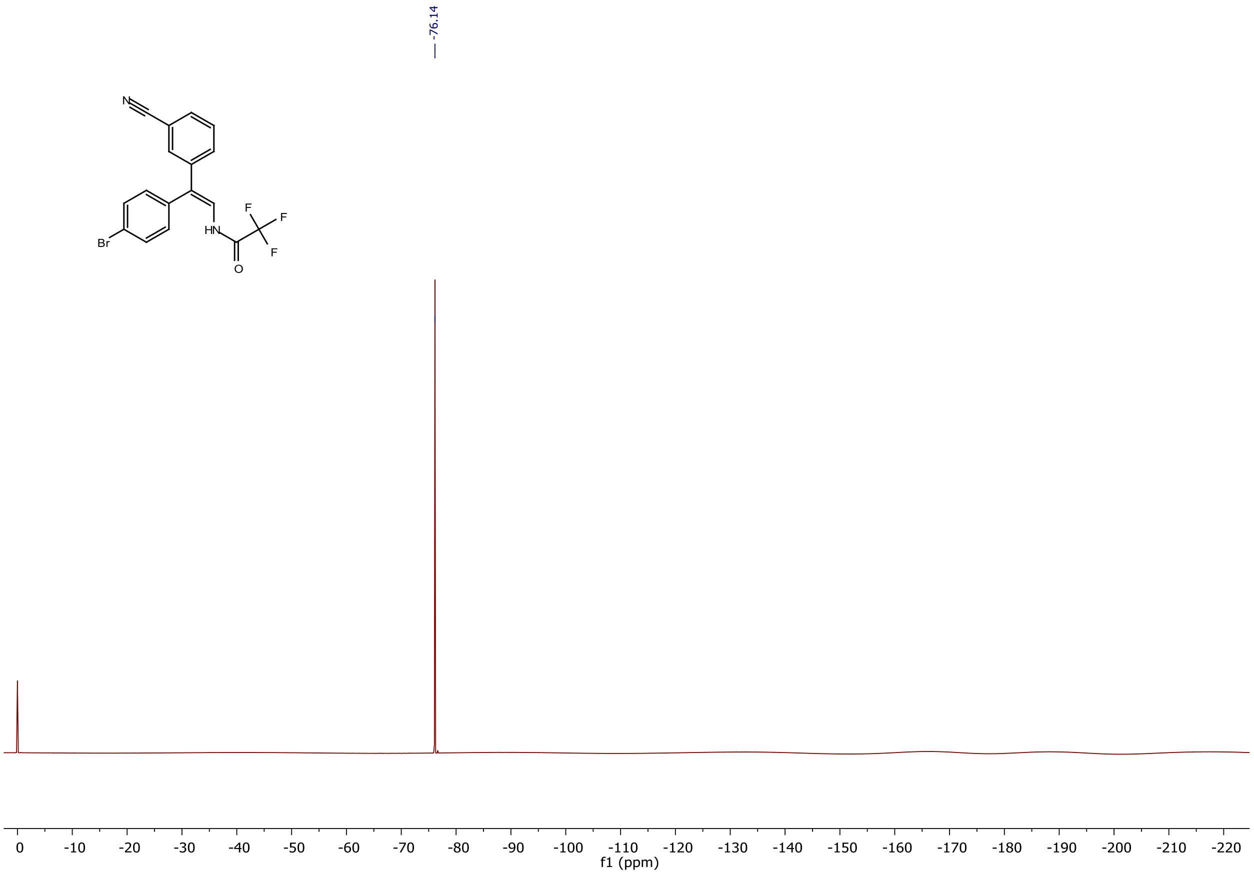

Figure S54. ^{19}F NMR spectrum of **3bb** ($CDCl_3$, 377 MHz)

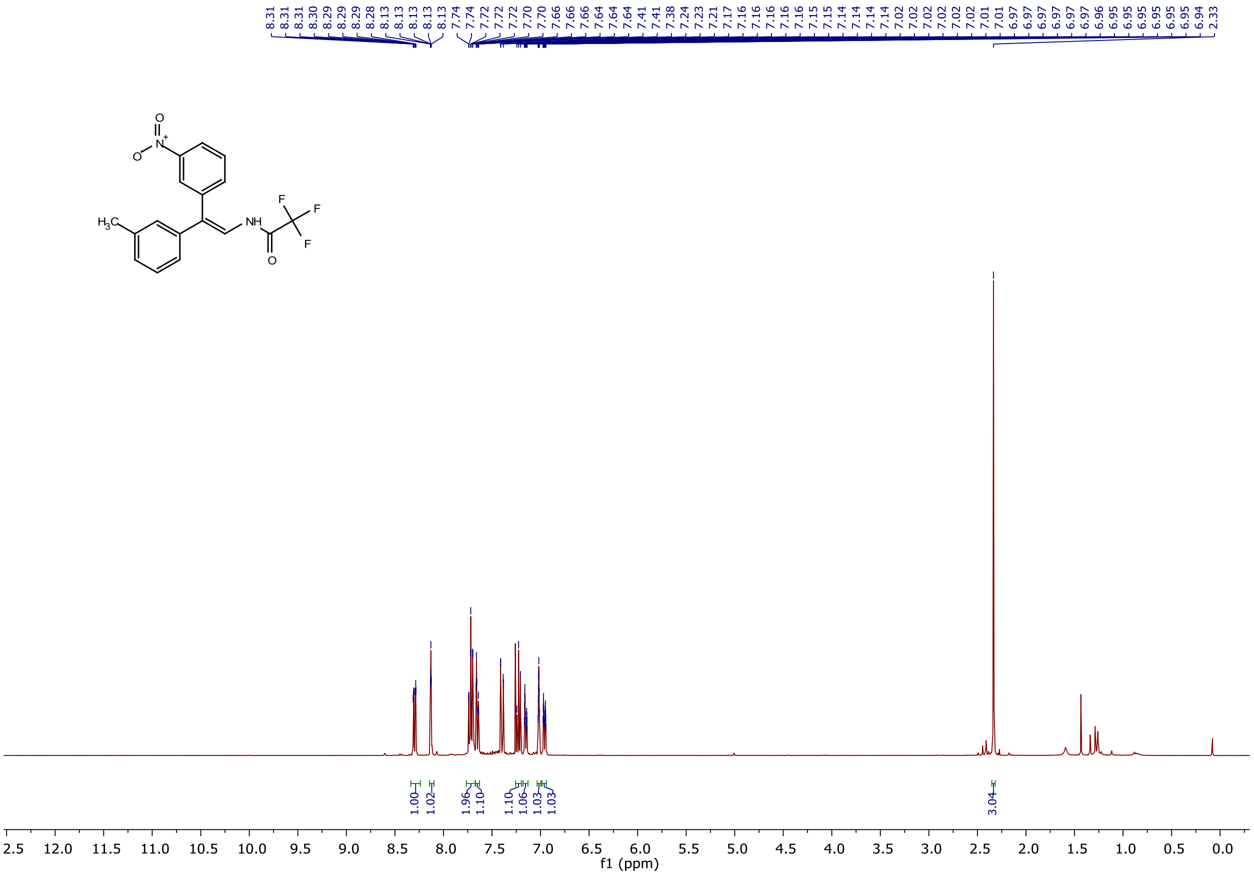

Figure S55. ^{1}H NMR spectrum of **2ca** ($CDCl_3$, 400 MHz)

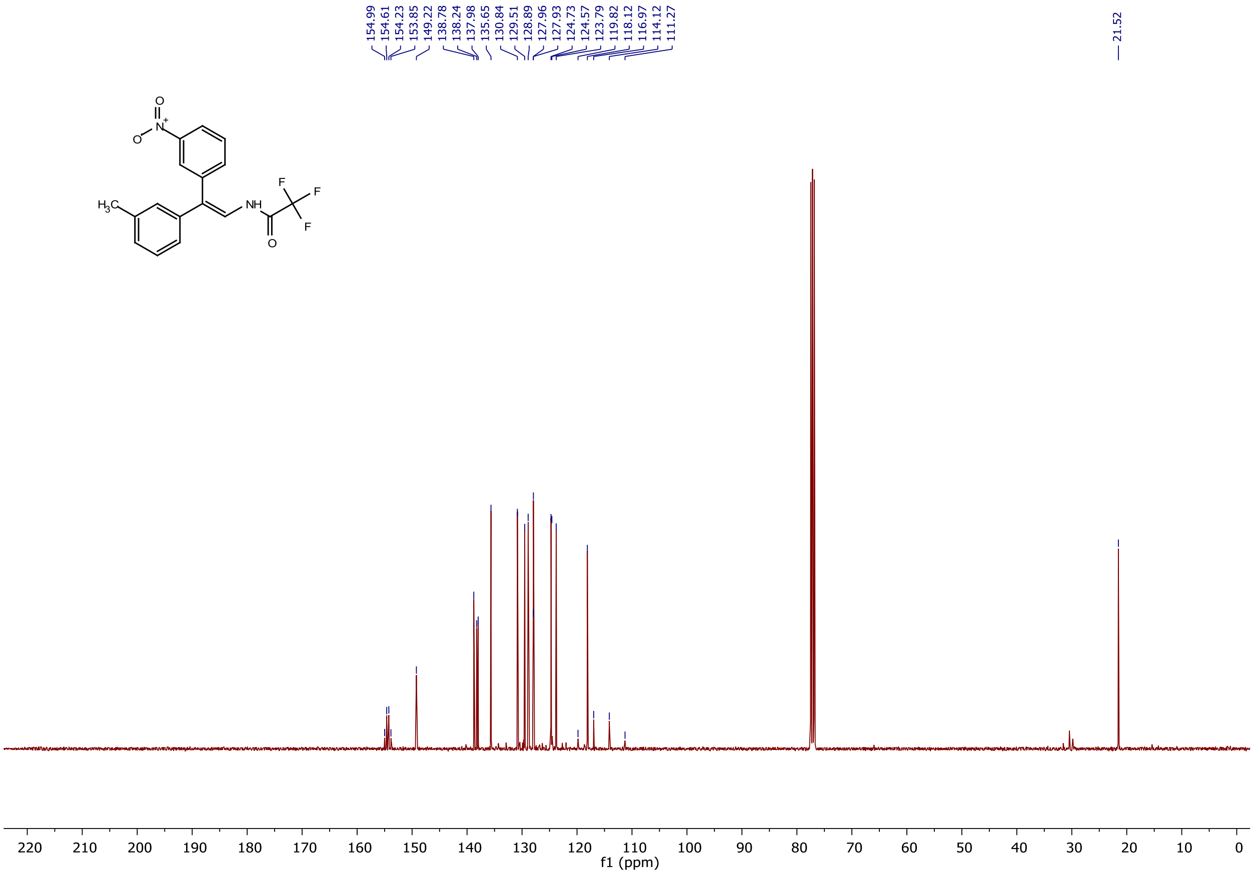

Figure S56. ^{13}C NMR spectrum of **2ca** ($CDCl_3$, 101 MHz)

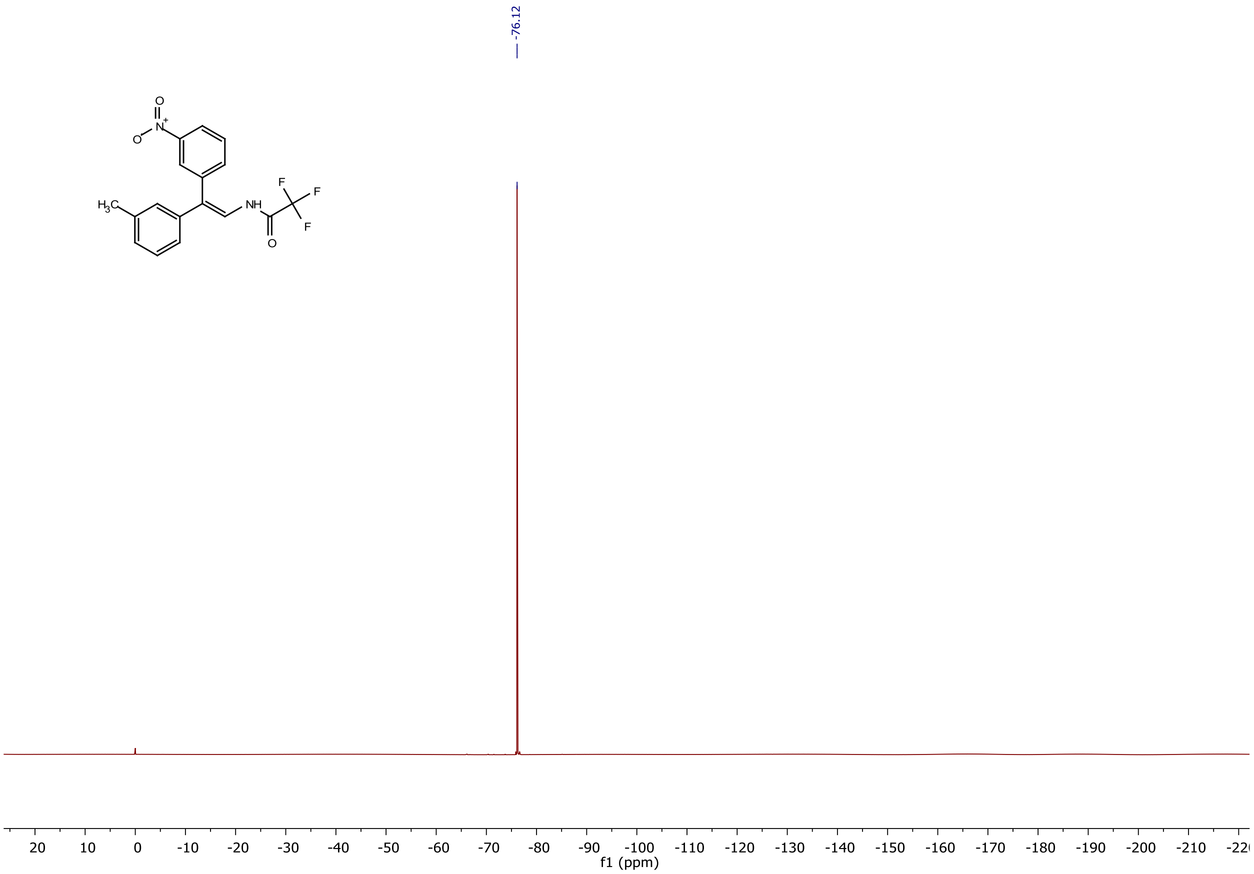

Figure S57. ^{19}F NMR spectrum of **2ca** ($CDCl_3$, 377 MHz)

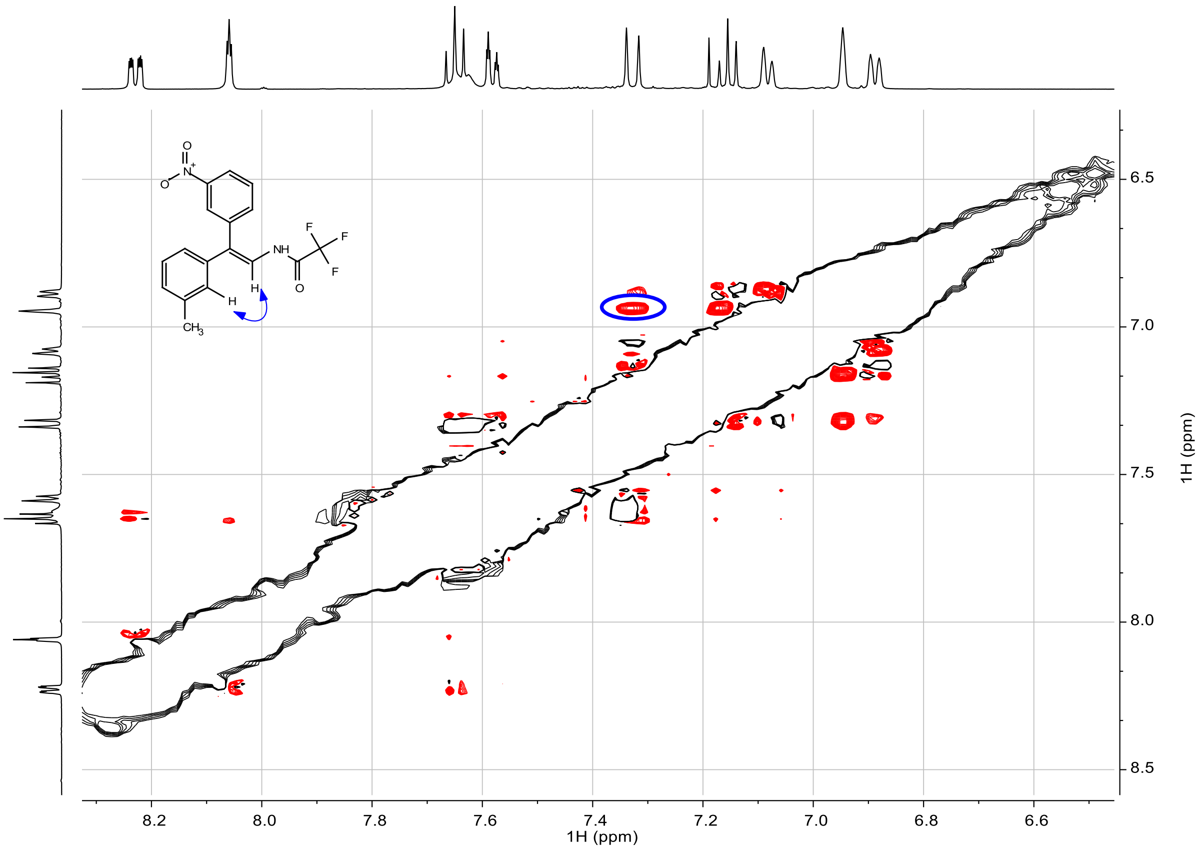

Figure S58. 2D ^{1}H-^{1}H ROESY NMR spectrum of **2ca** ($CDCl_3$, 500 MHz)

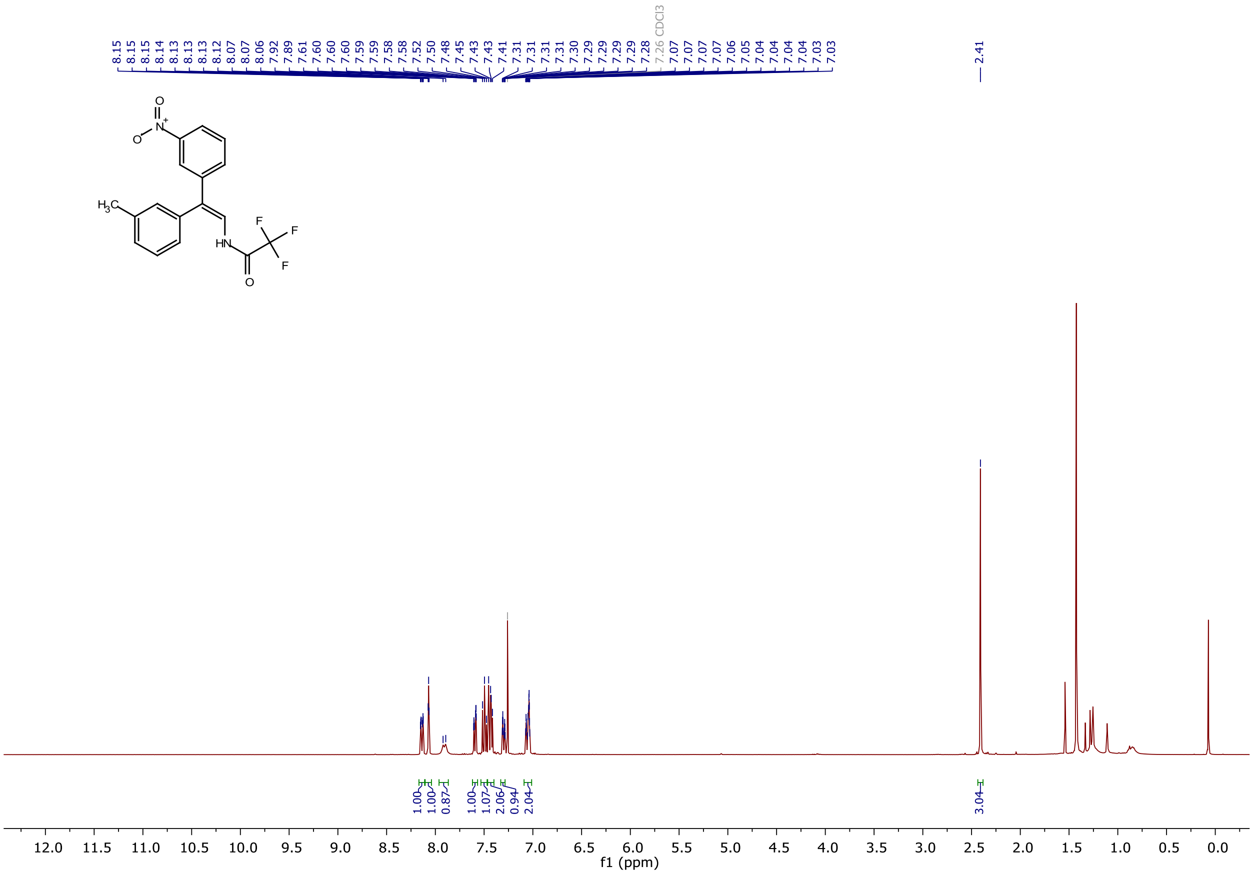

Figure S59. ^{1}H NMR spectrum of **3ca** ($CDCl_3$, 400 MHz)

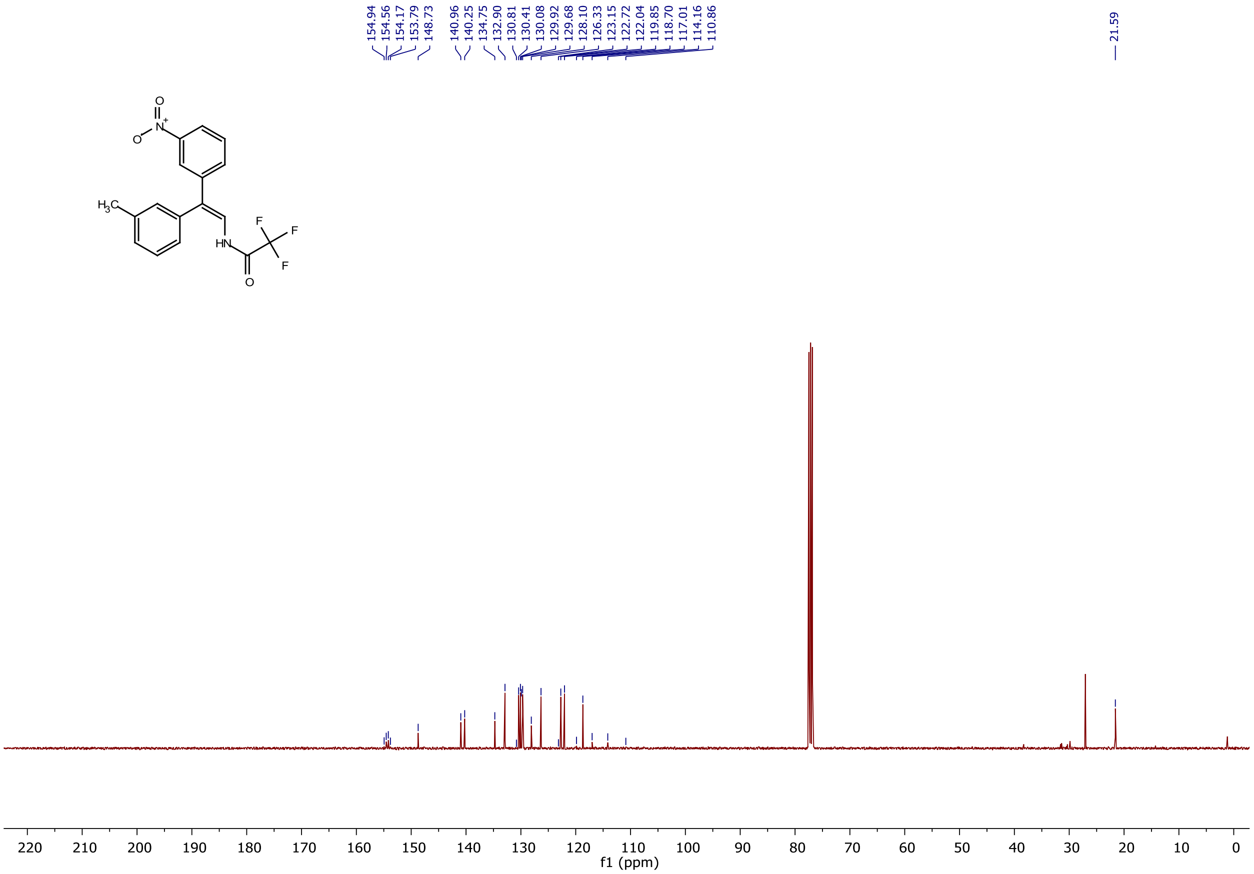

Figure S60. ^{13}C NMR spectrum of **3ca** ($CDCl_3$, 101 MHz)

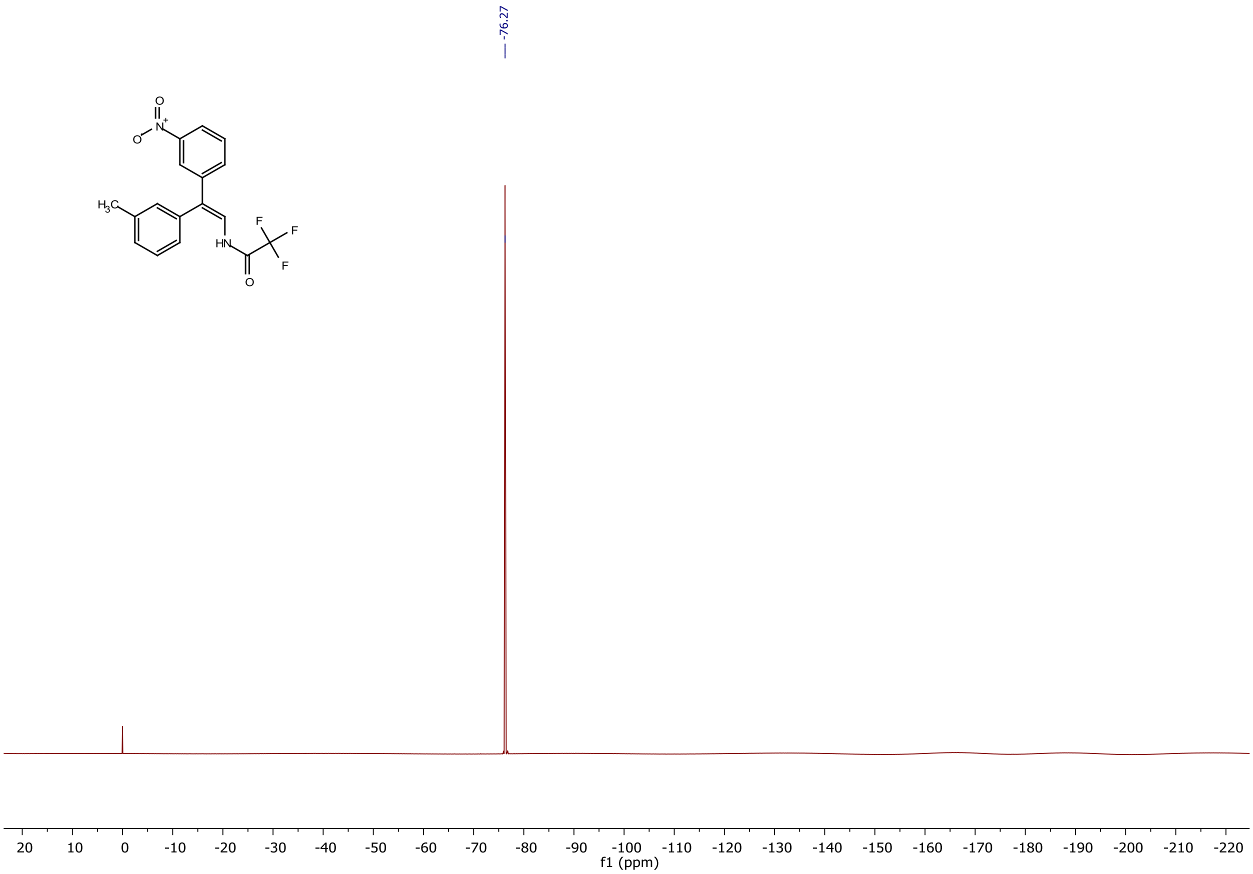

Figure S61. ^{19}F NMR spectrum of **3ca** ($CDCl_3$, 377 MHz)

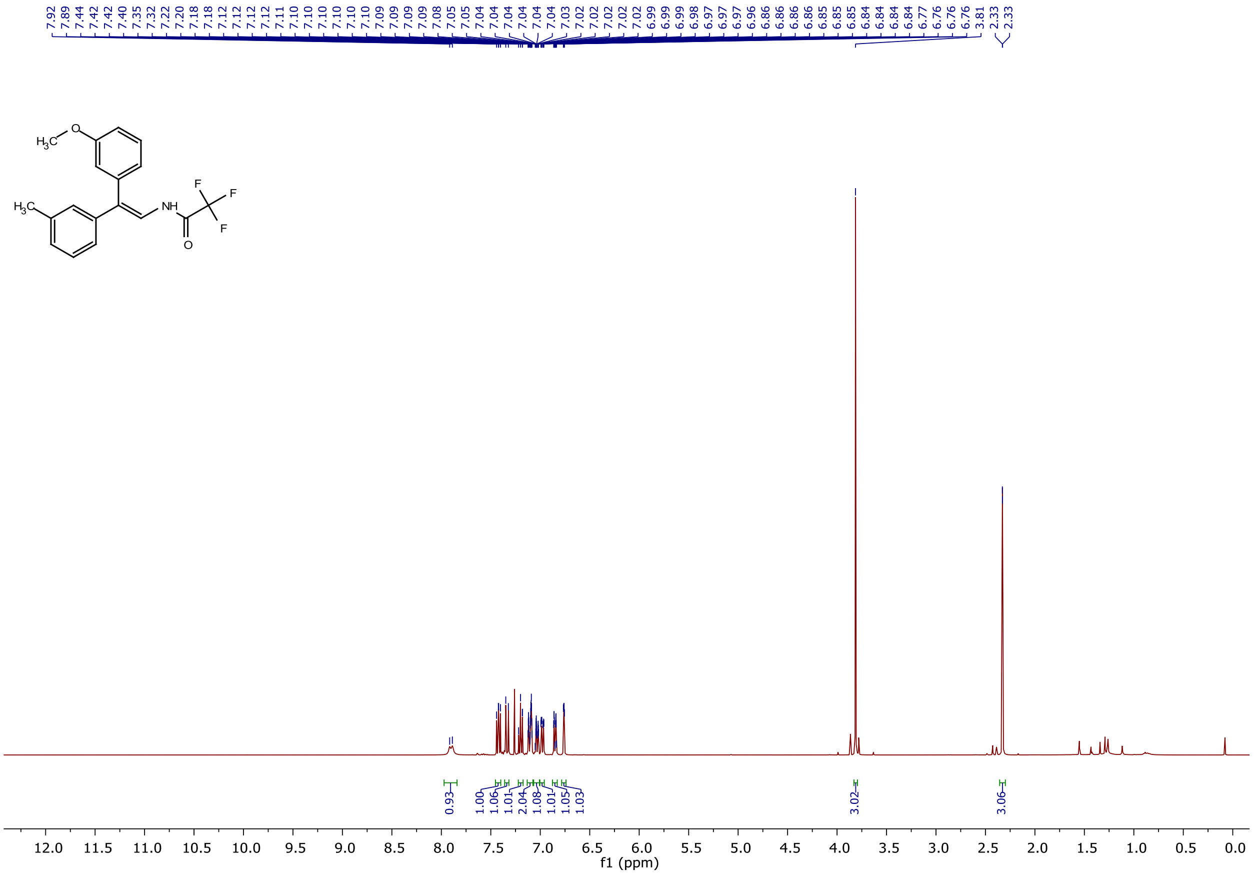

Figure S62. ^{1}H NMR spectrum of **2cc** ($CDCl_3$, 400 MHz)

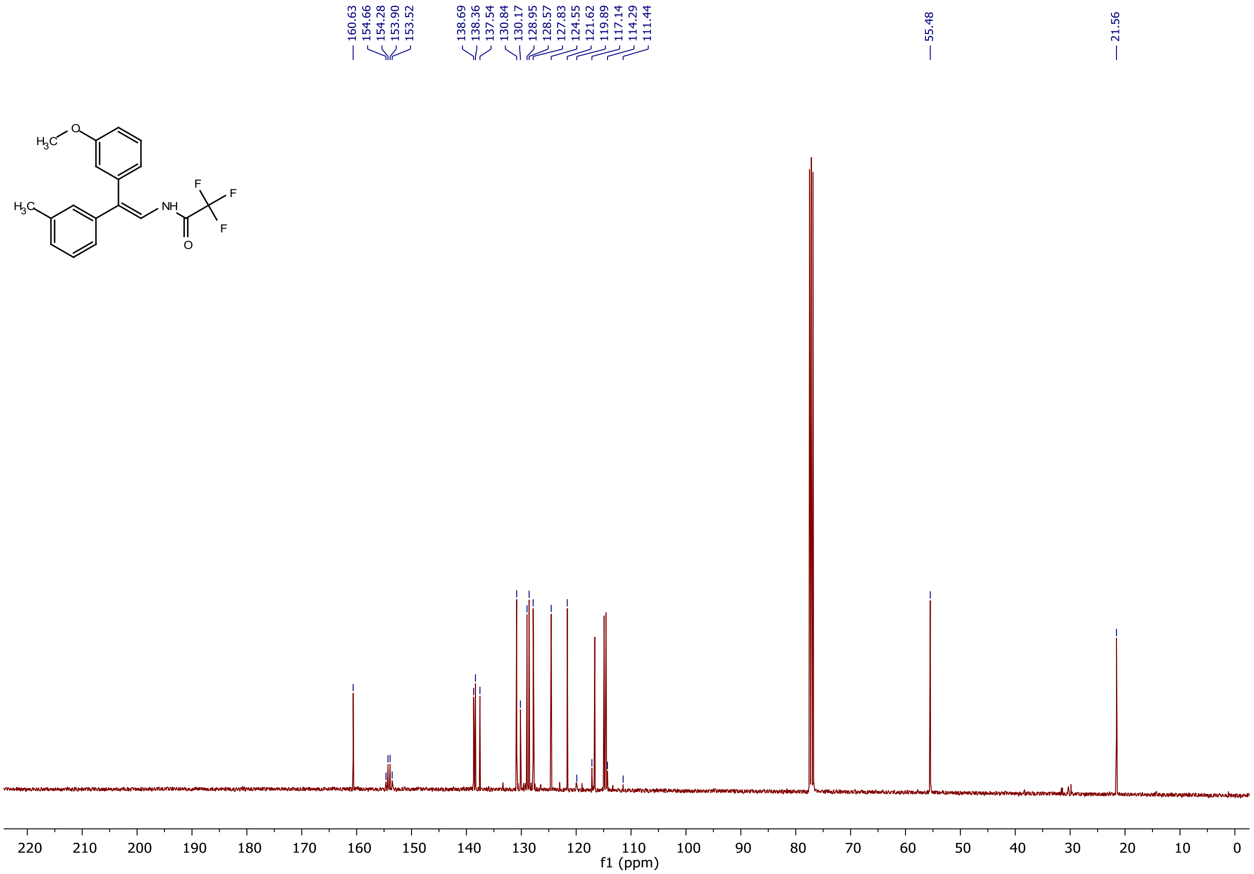

Figure S63. ^{13}C NMR spectrum of **2cc** ($CDCl_3$, 101 MHz)

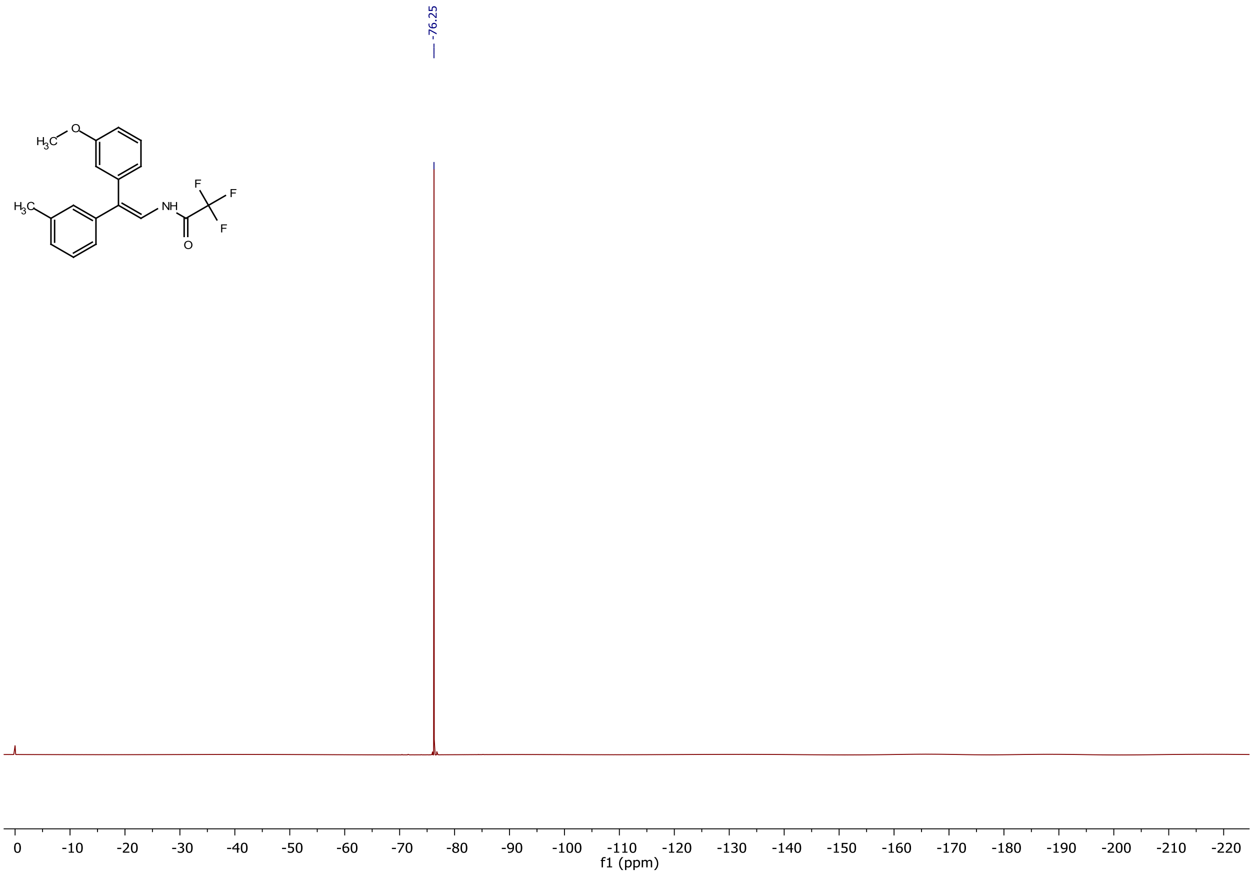

Figure S64. ^{19}F NMR spectrum of **2cc** ($CDCl_3$, 377 MHz)

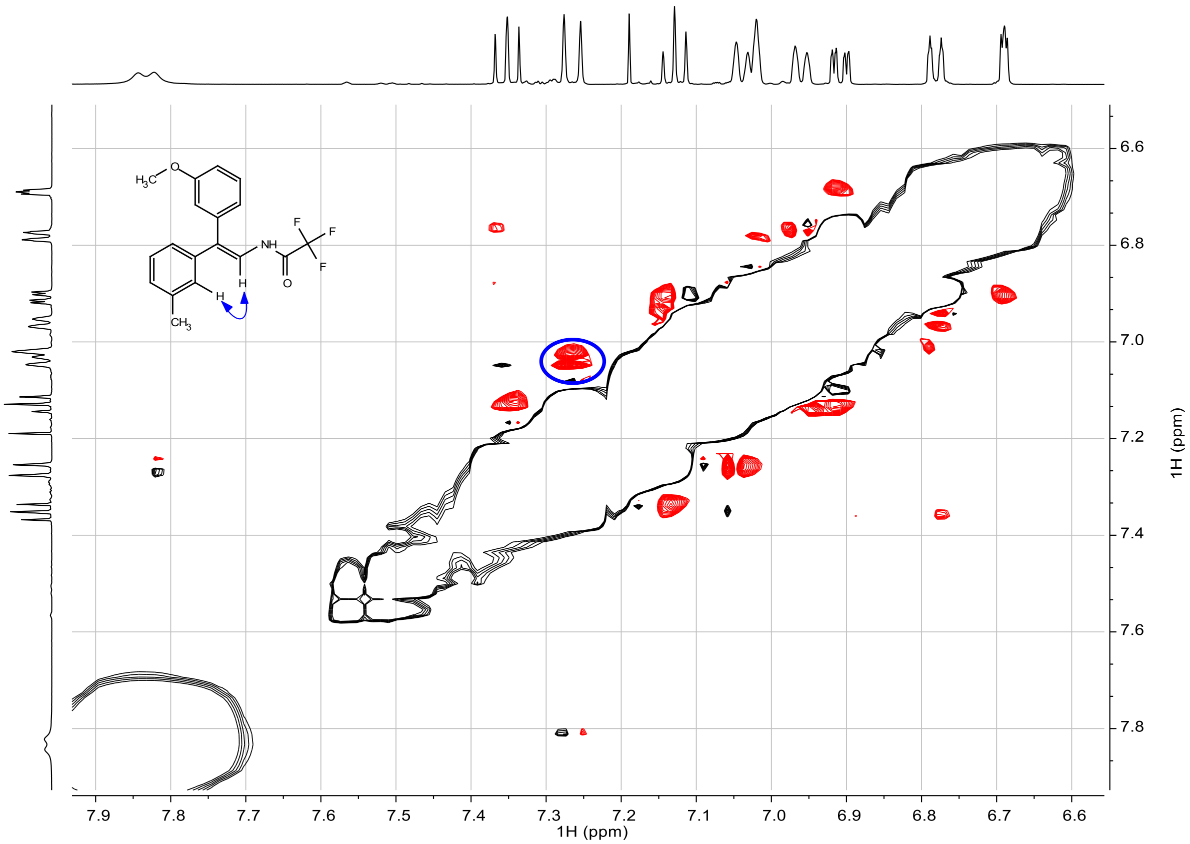

Figure S65. 2D ^1H-^1H ROESY NMR spectrum of **2cc** ($CDCl_3$, 500 MHz)

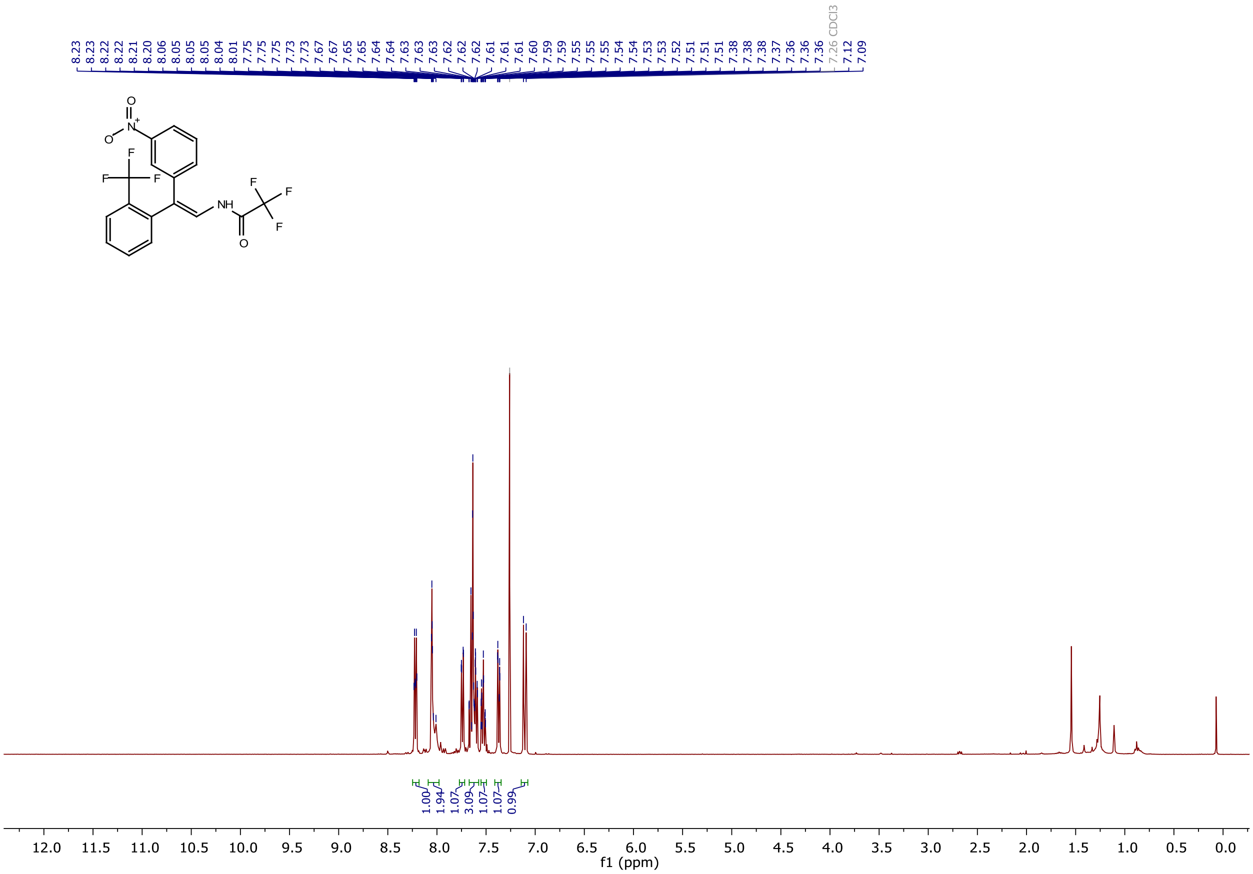

Figure S66. ^{1}H NMR spectrum of **2da** ($CDCl_3$, 400 MHz)

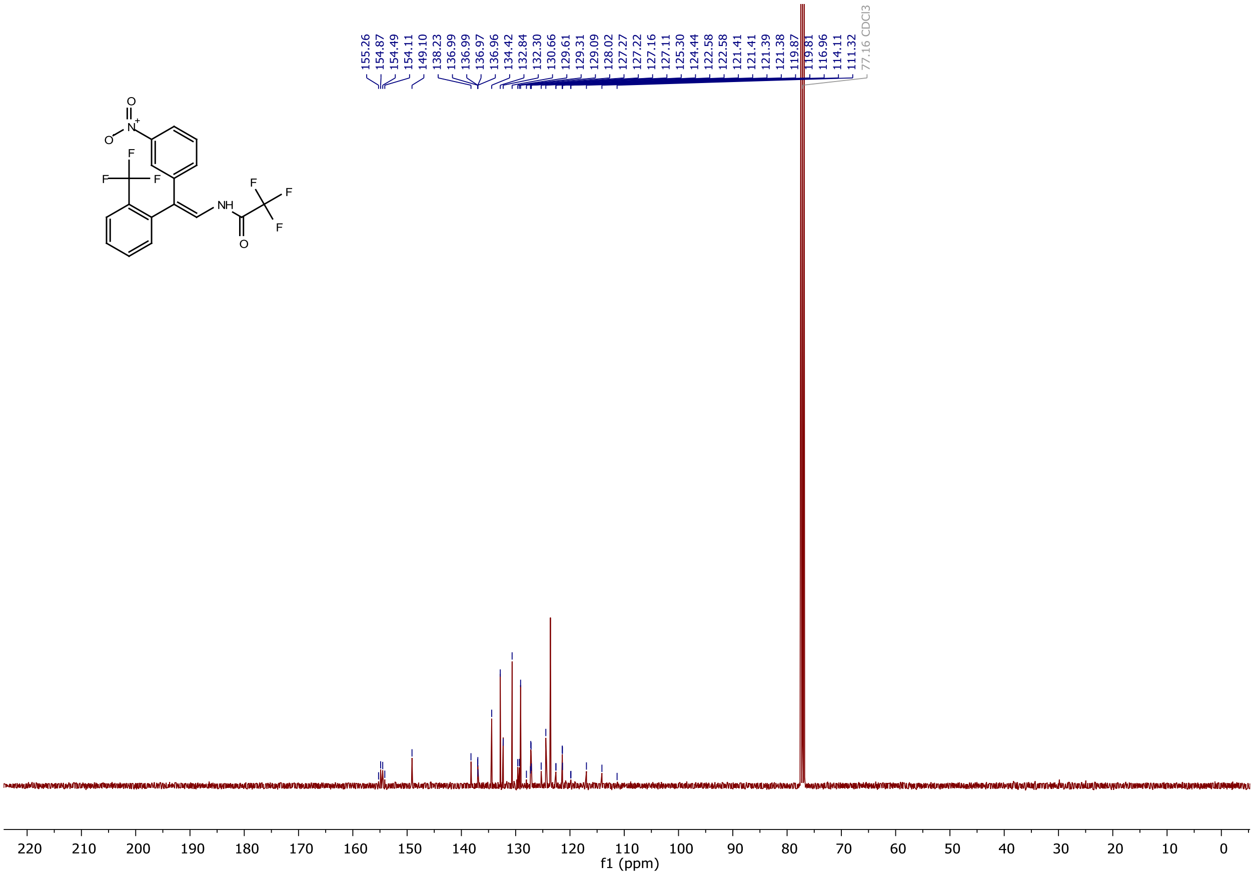

Figure S67. ^{13}C NMR spectrum of **2da** ($CDCl_3$, 101 MHz)

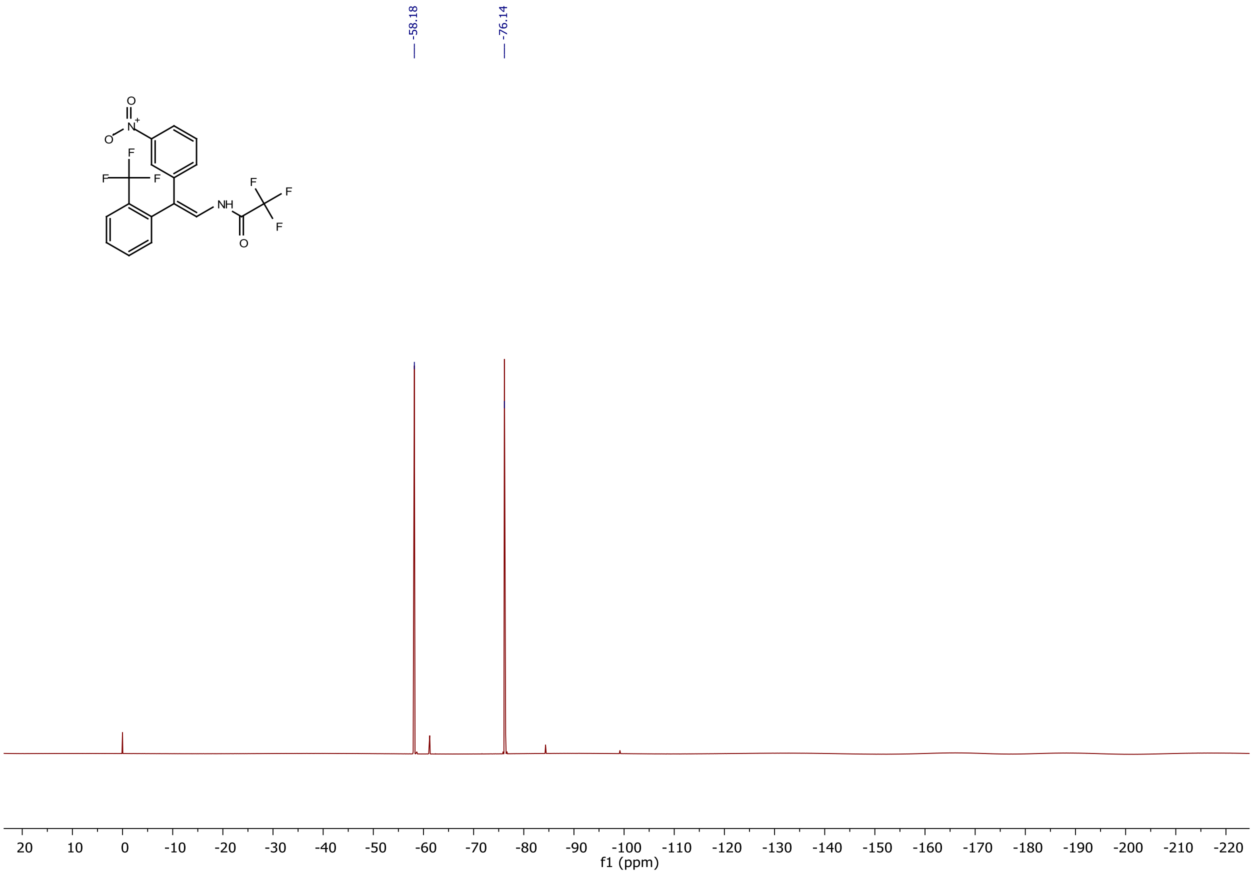

Figure S68. ^{19}F NMR spectrum of **2da** ($CDCl_3$, 377 MHz)

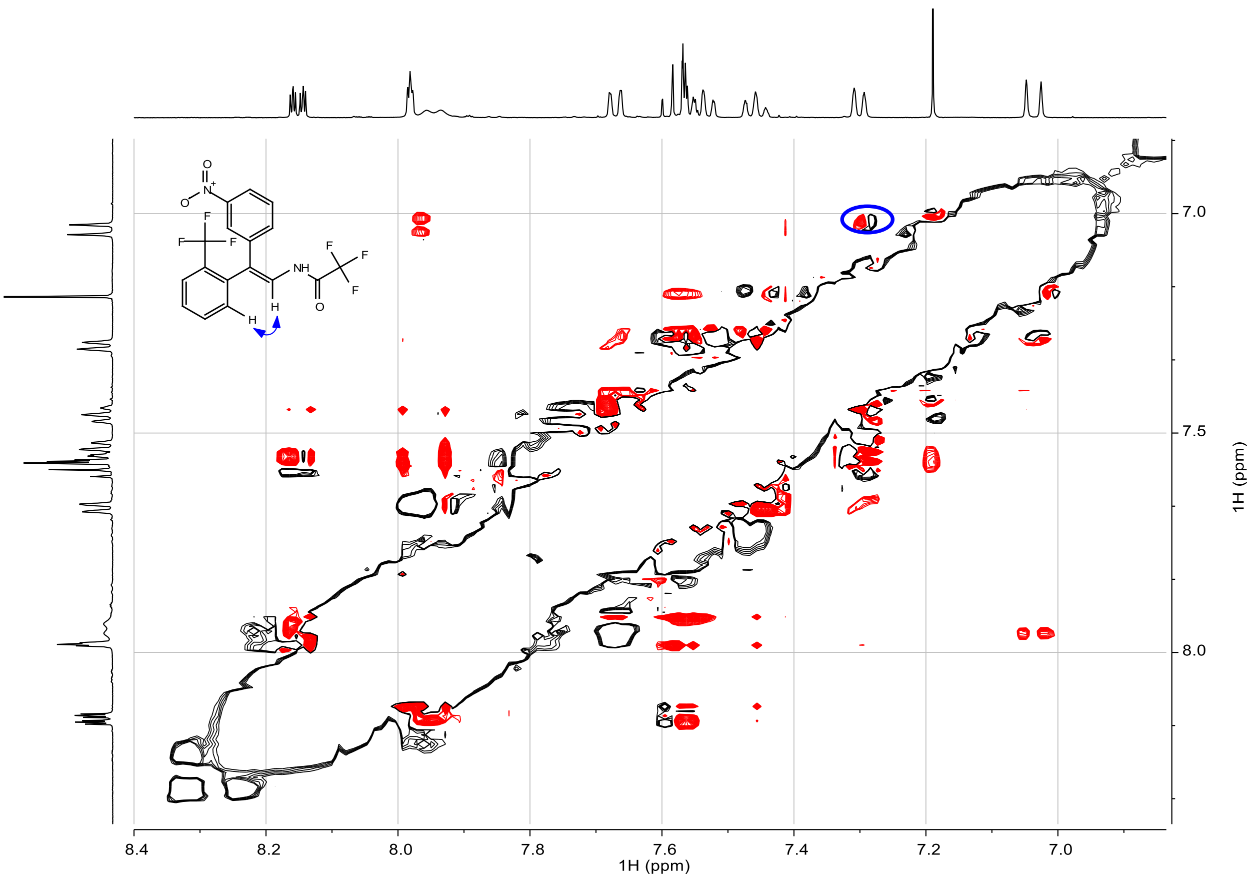

Figure S69. 2D ^{1}H-^{1}H ROESY NMR spectrum of **2da** ($CDCl_3$, 500 MHz)

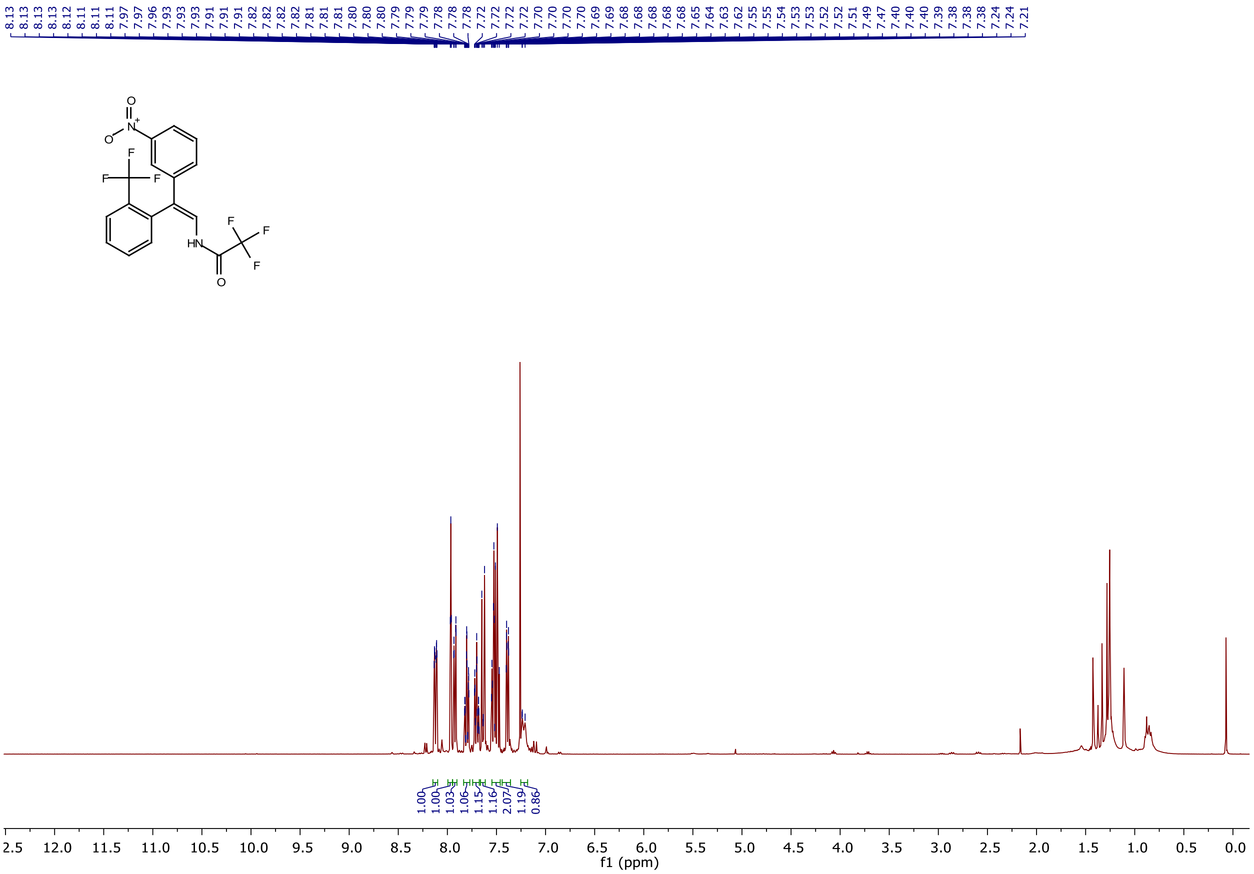

Figure S70. ^{1}H NMR spectrum of **3da** ($CDCl_3$, 400 MHz)

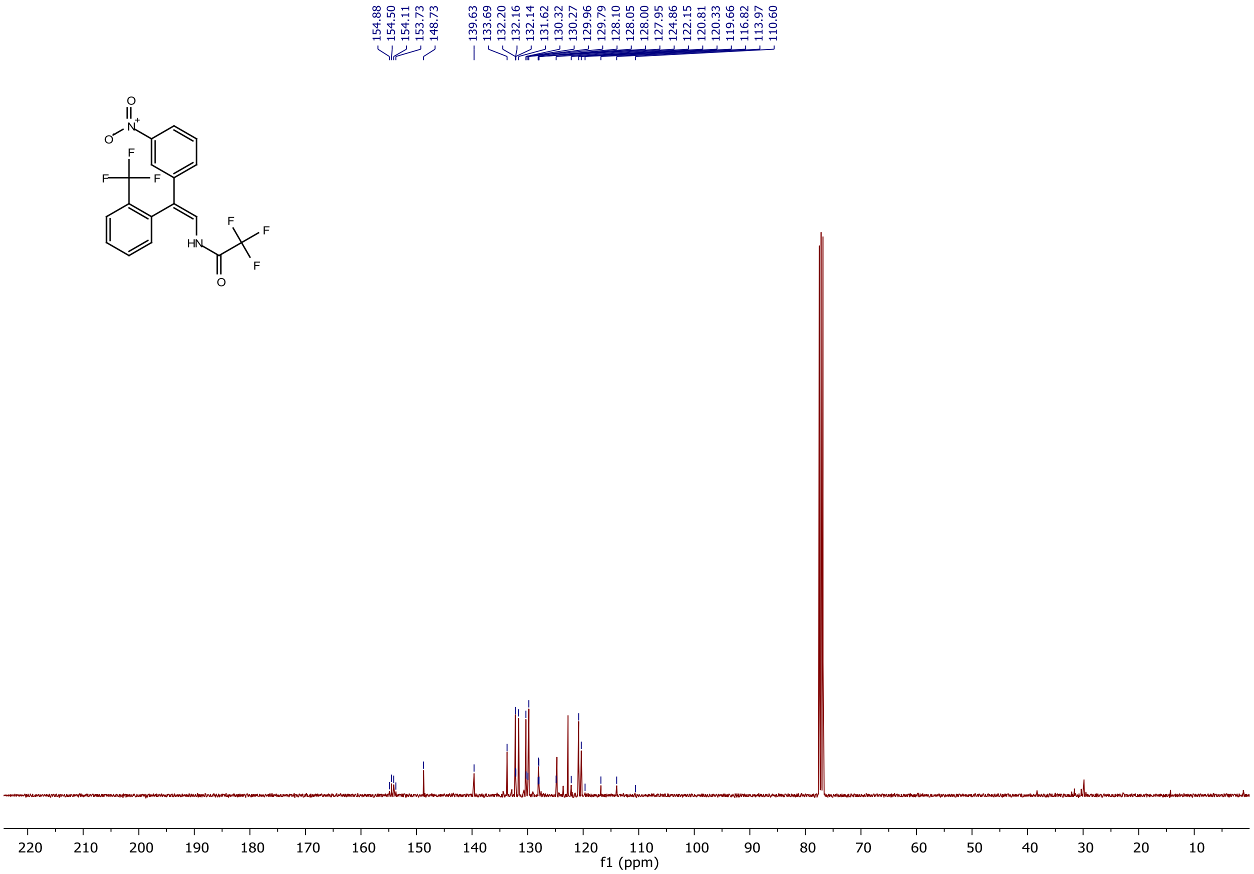

Figure S71. ^{13}C NMR spectrum of **3da** ($CDCl_3$, 101 MHz)

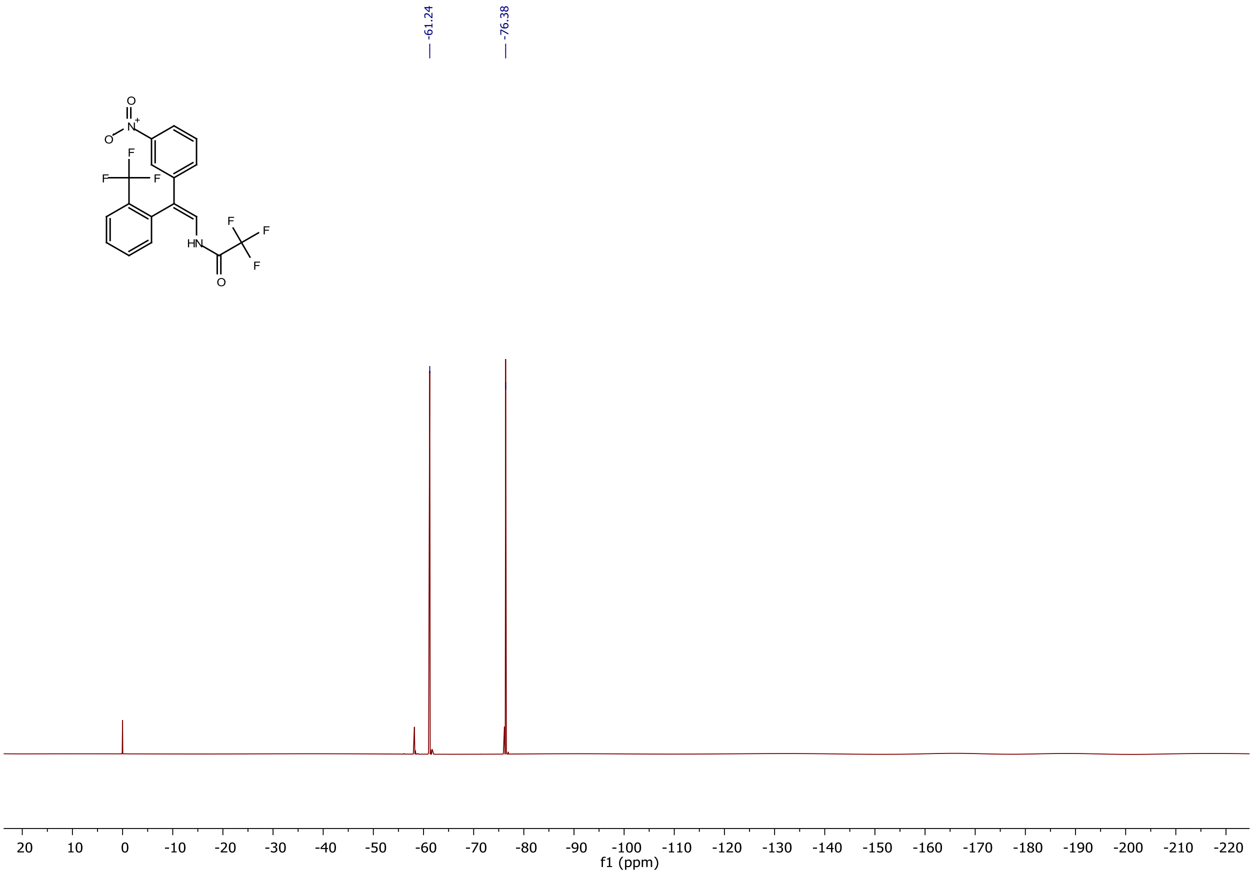

Figure S72. ^{19}F NMR spectrum of **3da** ($CDCl_3$, 377 MHz)

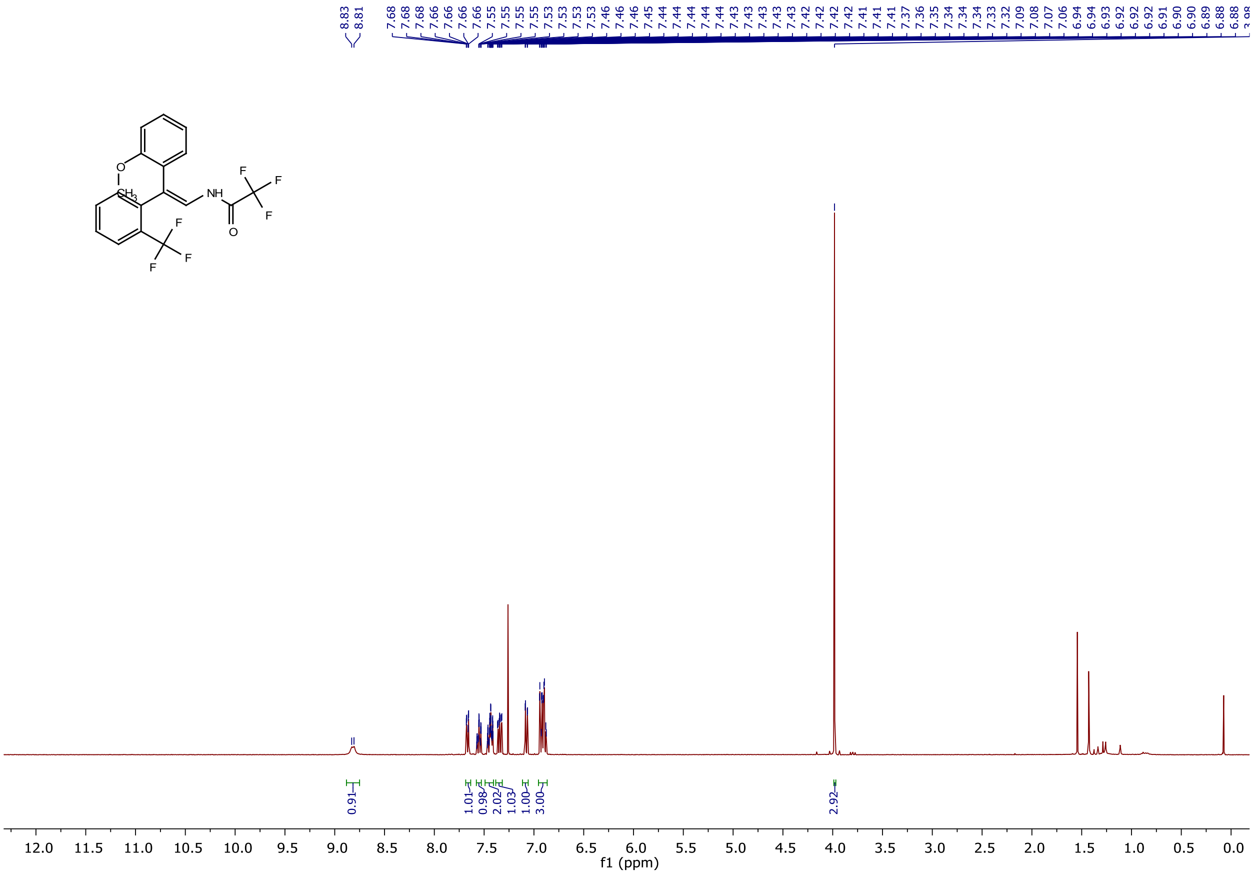

Figure S73. ^{1}H NMR spectrum of **2dd** ($CDCl_3$, 400 MHz)

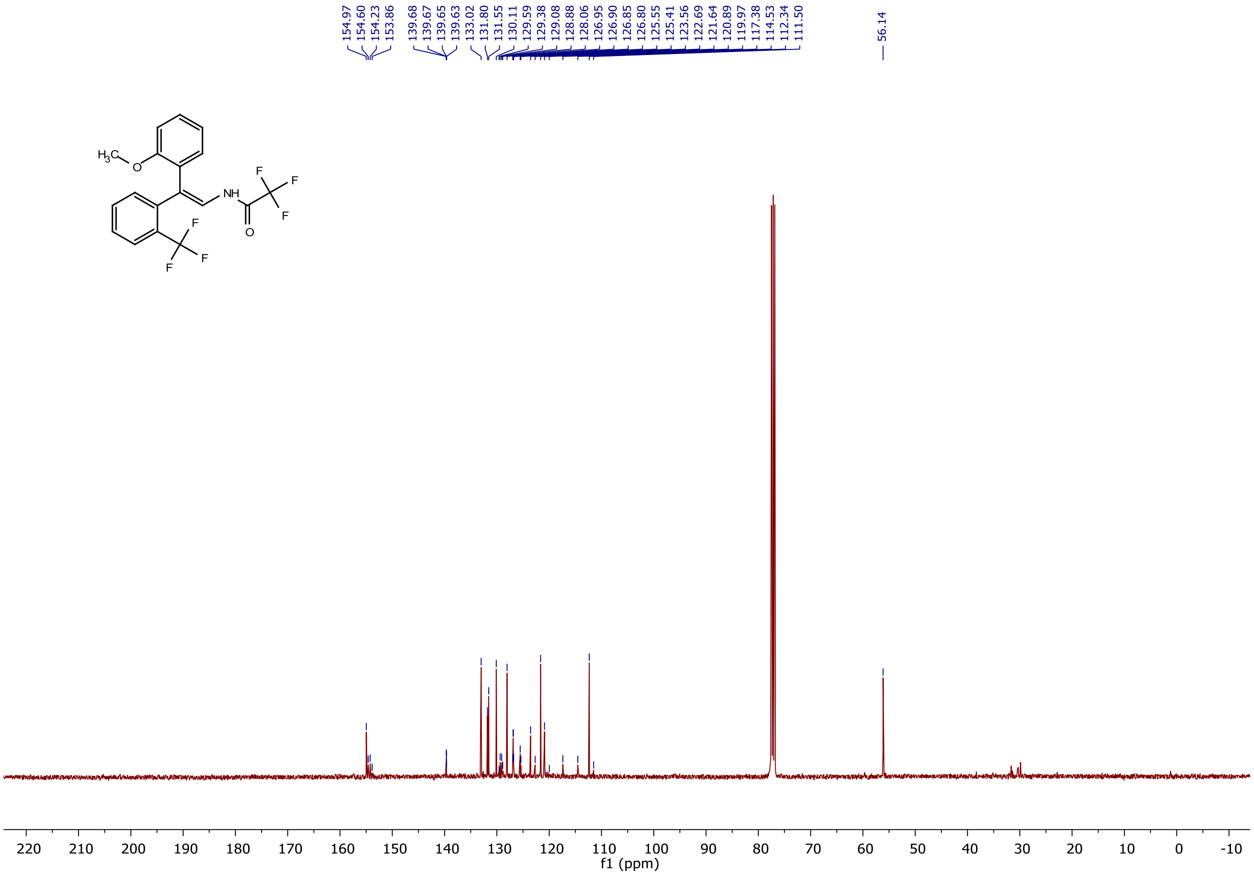

Figure S74. ^{13}C NMR spectrum of **2dd** ($CDCl_3$, 101 MHz)

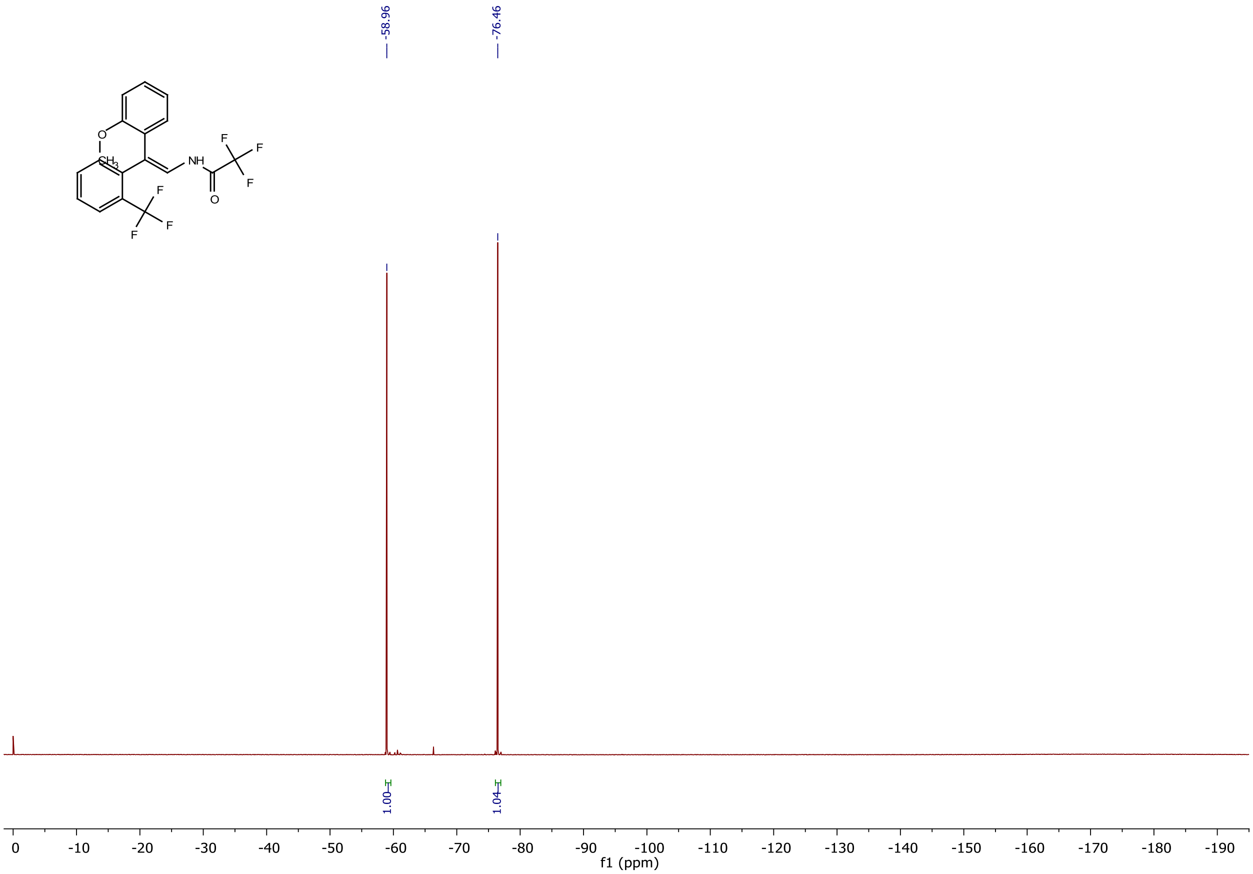

Figure S75. ^{19}F NMR spectrum of **2dd** ($CDCl_3$, 377 MHz)

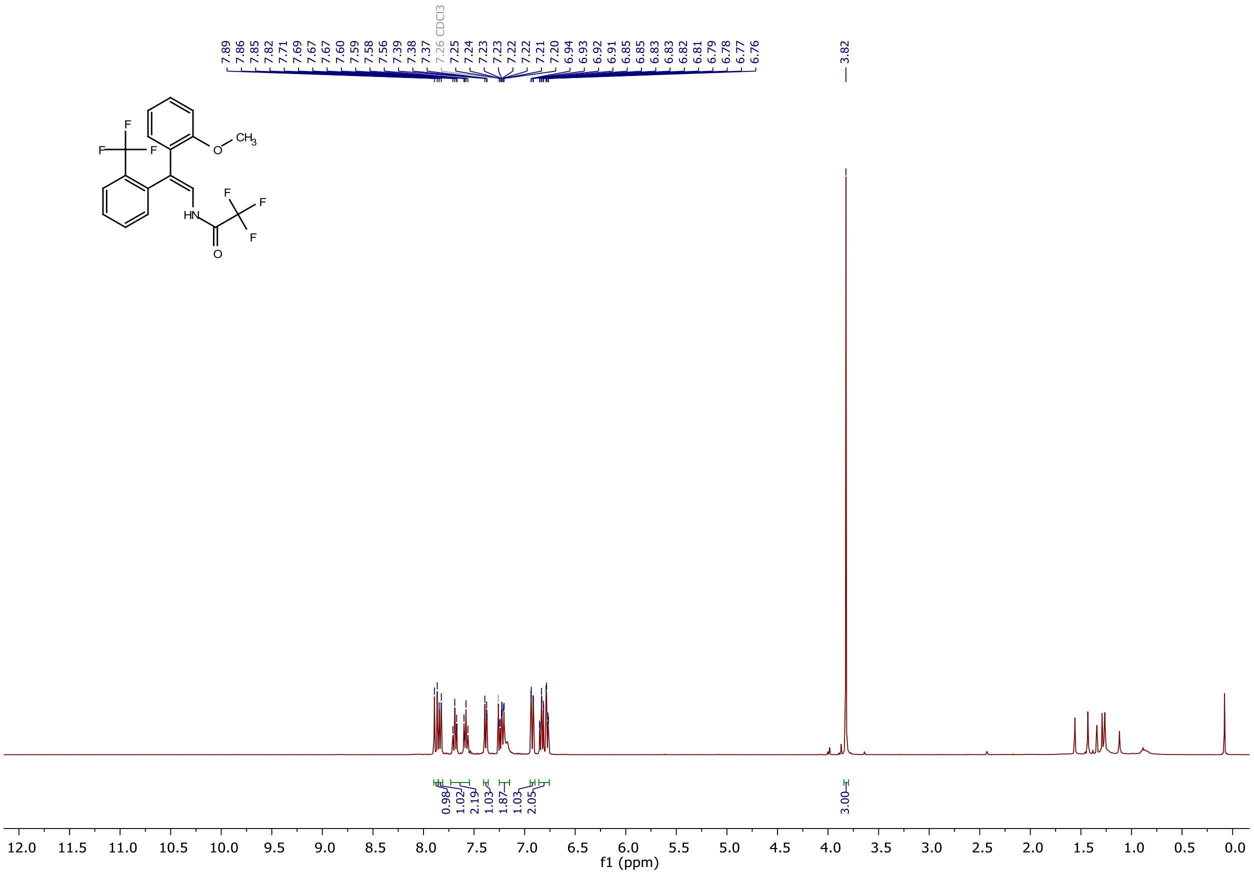

Figure S76. ^{1}H NMR spectrum of **3dd** ($CDCl_3$, 400 MHz)

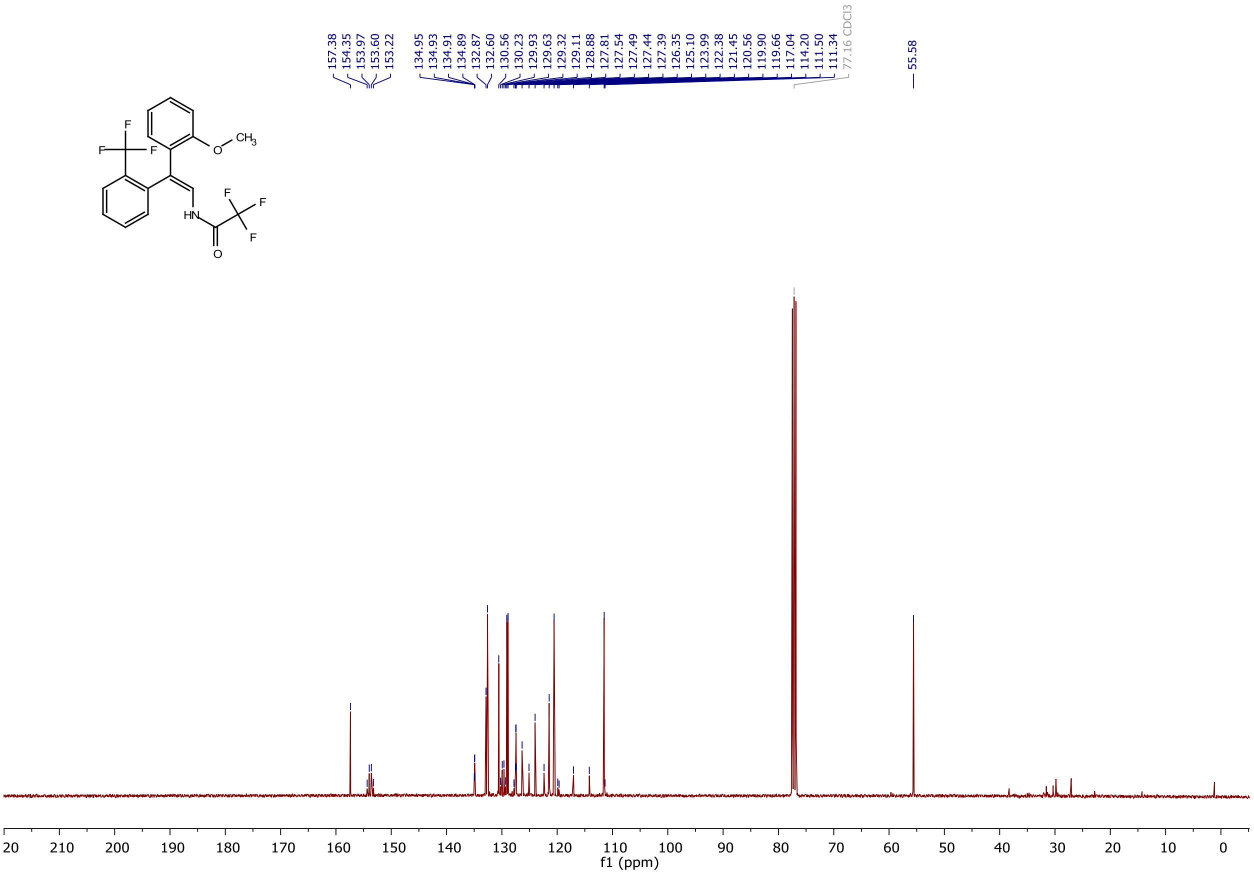

Figure S77. ^{13}C NMR spectrum of **3dd** ($CDCl_3$, 101 MHz)

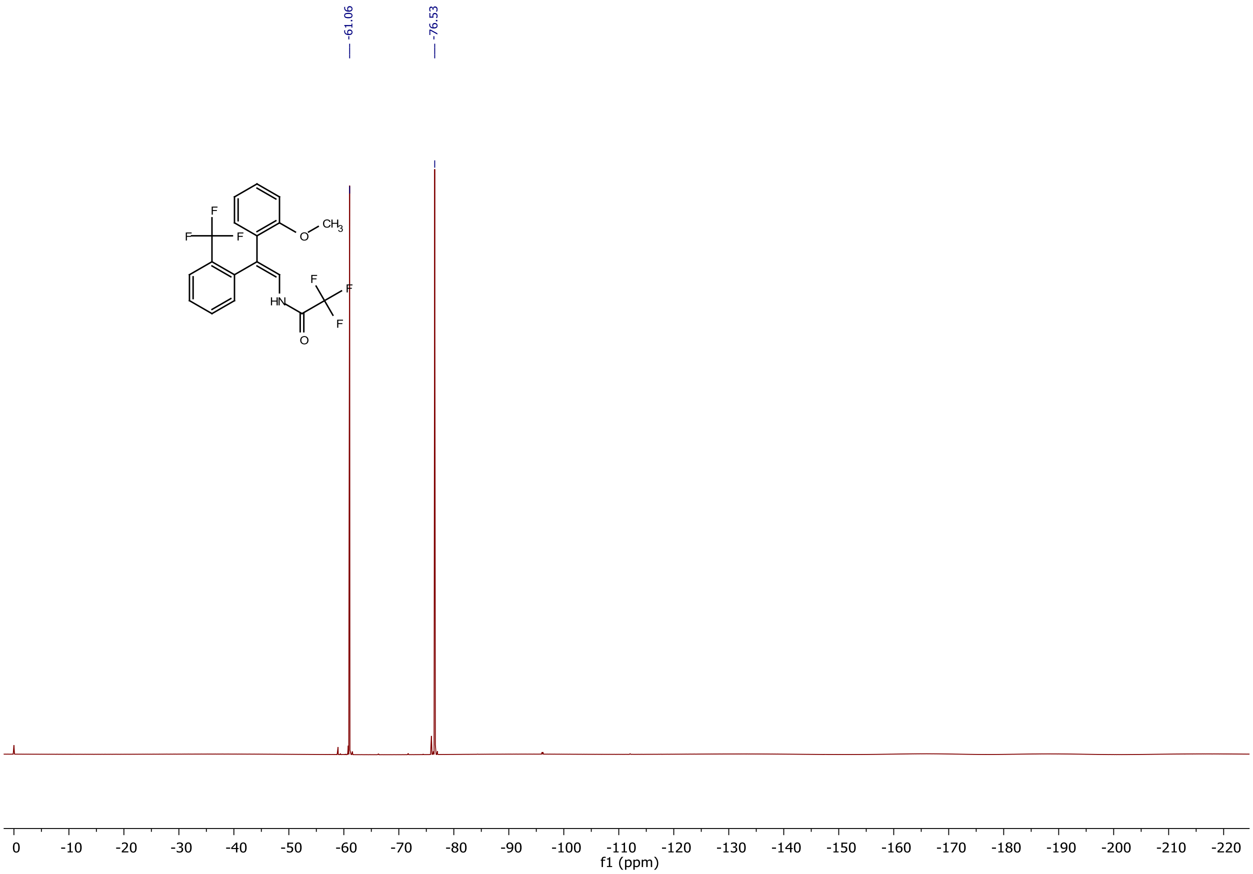

Figure S78. ^{19}F NMR spectrum of **3dd** ($CDCl_3$, 377 MHz)

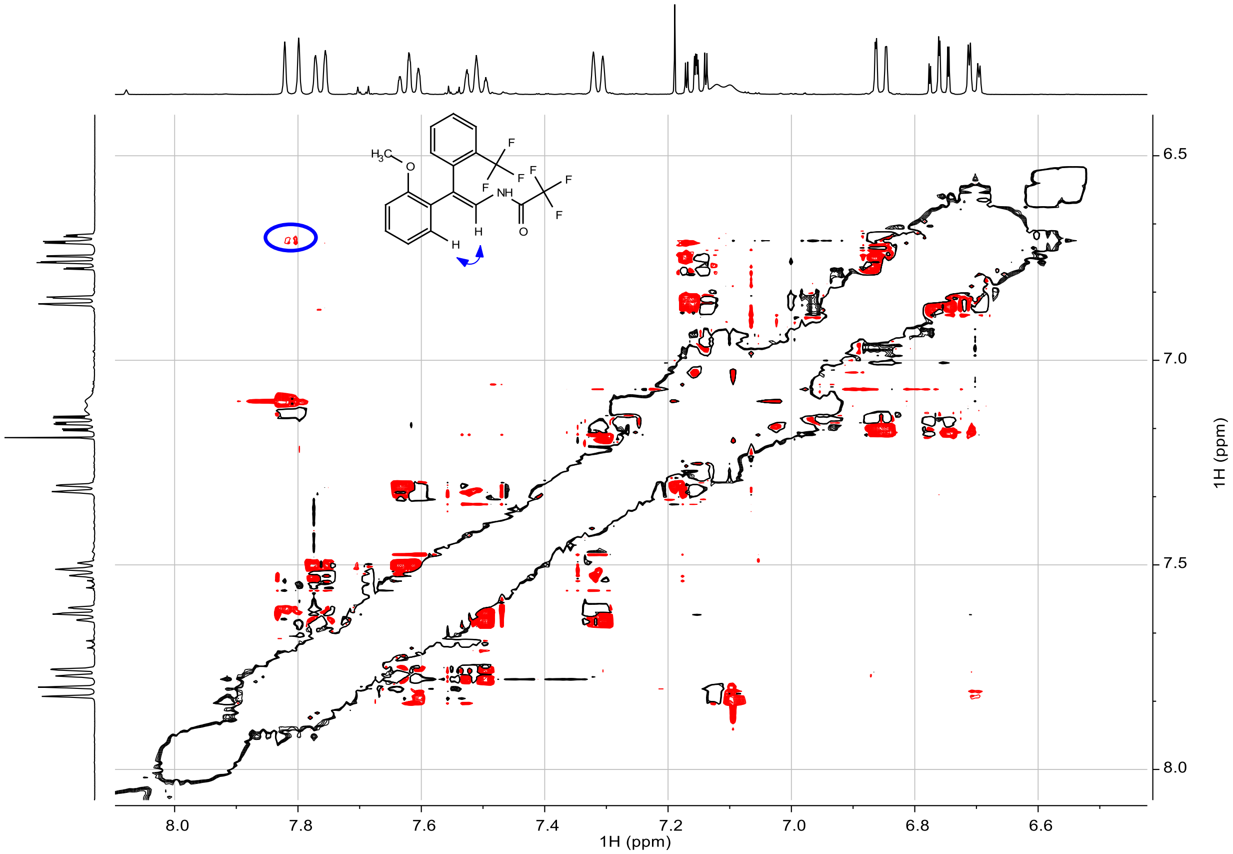

Figure S79. 2D ^{1}H-^{1}H ROESY NMR spectrum of **3dd** ($CDCl_3$, 500 MHz)
